# Preclinical Evaluation of Zn(II) Self‐Assemblies with Selective Cytotoxic Activity Against Cancer Cells In Vitro and *In Ovo*

**DOI:** 10.1002/chem.202302803

**Published:** 2023-11-21

**Authors:** Simon J. Allison, Gage P. Ashton, Hannah J. Lynch, Bethany R. Shire, Roger M. Phillips, Gareth M. B. Parkes, Emma Pinder, Craig R. Rice, Ana A. M. Teixeira, Tibo Volleman, Daisy A. Wordsworth

**Affiliations:** ^1^ School of Applied Sciences University of Huddersfield HD1 3DH Huddersfield UK; ^2^ Axion BioSystems Vrijstraat 9B 5611 AT Eindhoven The Netherlands

**Keywords:** cancer, cytotoxic, helicate, self-assembly, zinc

## Abstract

Dipodal pyridylthiazole amine ligands **L^1^** and **L^2^** both form different metallo‐supramolecular self‐assemblies with Zn^2+^ and Cu^2+^ and these are shown to be toxic and selective towards cancer cell lines in vitro. Furthermore, potency and selectivity are highly dependent upon the metal ions, ligand system and bound anion, with significant changes in chemosensitivity and selectivity dependent upon which species are employed. Importantly, significant anti‐tumor activity was observed *in ovo* at doses that are non‐toxic.

## Graphical abstract

Bis‐bidentate ligands **L^1^** and **L^2^** form dinuclear double helicates with Zn^2+^ and Cu^2+^ and these self‐assemblies can bind phosphate and 4‐nitrophenylphosphate anions. The complexes show selective cytotoxic and antitumor activity, but this is highly dependent upon which ligand, metal ion and anion are used. The lead compound [(**L^2^**)_2_Zn_2_]^2+^ displays *in ovo* efficacy with a 67 % reduction in tumor weight and 100 % embryo survival rate.

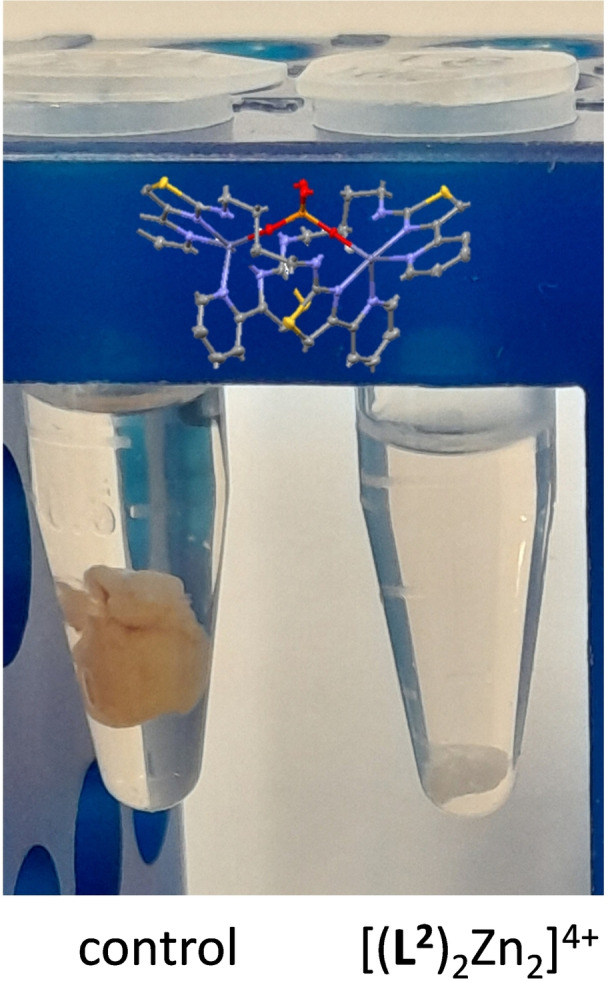

## Introduction

Metallosupramolecular chemistry is the use of preprogramed poly‐dentate ligand systems that upon coordination to metal ions self‐assemble into predictable structures by means of directional information encoded within both the metal ion (M^n+^) and ligand (L) precursors. This type of chemistry is attractive as a diverse range of supramolecular topologies are possible by the simple mixing of different M^n+^ and L components.^[1]^ As a consequence of this, the use of self‐assembled metallo‐supramolecular species for multiple applications including the development of novel cancer therapeutics is of considerable interest as it offers easy and rapid access to a library of novel molecularly complex architectures all of which may have varied biological activity generated via multiple mechanisms of action.^[2]^ Whilst notable examples of self‐assembled metallo‐supramolecular structures with cytotoxic activity include Hannon's Fe^2+^‐containing helicates,^[3]^ and Scott's “head‐to‐head‐to‐tail” helicates^[4]^ this field is yet to fully mature and remains in the nascent stages of the cancer drug discovery process.

Using the phenotypic approach to drug discovery, we have previously demonstrated that a tripodal ligand (**L**) self‐assembles with Cu^2+^, Zn^2+^ and Mn^2+^ metal (M) ions to form trinuclear species (e. g. [**L**_2_M_3_]^6+^) that have potent and highly selective activity against a range of cancer cell lines in vitro.^[5]^ Their mechanism of action involves the inhibition of multiple kinases that play a significant role in cancer biology via either binding to or hydrolysing phosphoregulatory sites on the kinase enzyme.^[5]^

In this study, we show that ligands **L^1^** and **L^2^** self‐assemble with Zn^2+^ to form dinuclear species and these can bind dihydrogen phosphate and *p*‐nitrophenyl phosphate anions, with the latter changing the self‐assembled species to a pentametallic assembly. Using a similar phenotypic approach to drug discovery, we show that these species are potent cytotoxins with selectivity for cancer cell lines in vitro and *in ovo*. Furthermore, the activity and selectivity are highly dependent upon the metal ions, ligand system and bound anion, with significant changes in chemosensitivity and selectivity dependent upon which species are assembled. This allows us to take full advantage of the metallosupramolecular self‐assembly process allowing construction of a large range of different species from easily synthesized components, all of which show different cytotoxic potencies and selectivity profiles.

## Results and Discussion

**Figure 1:**
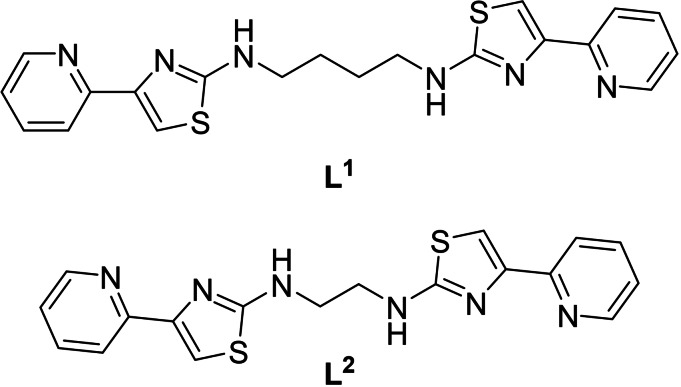
Ligands **L^1^** and **L^2^**.

**Figure 2:**
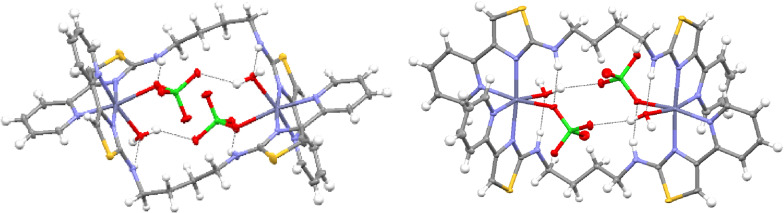
X‐ray structure of [(**L^1^**)_2_Zn_2_(H_2_O)_2_(ClO_4_)_2_]^2+^. Thermal ellipsoids shown at the 50 % probability level. Selected anions omitted for clarity. Color code: dark blue, Zn(II); red, O; blue, N; yellow, S; green Cl; grey, C.

Both ligands **L^1^** and **L^2^** contain two potentially bidentate pyridyl‐thiazole domains separated by a 1,4‐diaminobutane and a 1,2‐diaminoethane spacer group respectively (Figure 1). Reaction of **L^1^** with Zn(ClO_4_)_2_ ⋅ 6H_2_O in MeCN gave a colorless solution which deposited a small amount of crystalline material upon diffusion of dichloromethane. Solid‐state analysis showed the formation of a dinuclear species with two 6‐coordinate Zn^2+^ ions comprised of two bidentate pyridyl‐thiazole domains from two different ligands, a perchlorate counter ion and a single water molecule (Figure 2). This gives rise to a dinuclear assembly for example [(**L^1^**)_2_Zn_2_(H_2_O)_2_(ClO_4_)_2_]^2+^ but as there is no “twist” about the two binding domains this would be classed as a *mesocate* assembly. Also contained within the structure are assembly‐guest hydrogen bonding interactions, with a ‐NH⋅⋅⋅O hydrogen bond between one of the amine units and the coordinated oxygen atom of the perchlorate anion. The remaining amine forms a hydrogen bond to the coordinated water molecule.

Analysis of this material by ESI‐MS showed an ion at *m/z* 1245 which corresponds to {[(**L^1^**)_2_Zn_2_](ClO_4_)_3_}^+^ with ion at *m/z* 573 corresponding to {[(**L^1^**)_2_Zn_2_](ClO_4_)_2_}^2+^ and {[(**L^1^**)Zn](ClO_4_)}^+^.

**Figure 3:**
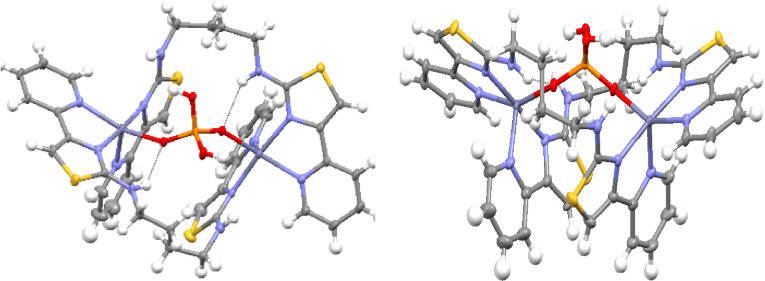
X‐ray structure of [(**L^1^**)_2_Zn_2_(H_2_PO_4_)_2_]^3+^. Thermal ellipsoids shown at the 50 % probability level. Selected anions omitted for clarity. Color code: dark blue, Zn(II); red, O; blue, N; yellow, S; grey, C; orange P.

Reaction of **L^1^** with Zn(ClO_4_)_2_ ⋅ 6H_2_O and half an equivalent of Bu_4_NH_2_PO_4_ gave a pale yellow solution from which crystals were deposited upon slow diffusion of dichloromethane (Figure 3). In the solid‐state the ligand again partitions into two separate binding domains with each metal center coordinated by two bidentate units from separate ligands. However, also contained within the assembly is a phosphate counter anion which coordinates via two oxygen atoms, bridging the two metal ions. Each of the metal ions adopt a five‐coordinate geometry, formed from the four *N*‐donor ligand units and one phosphate oxygen atom. Whilst the X‐ray data is not of sufficient quality to locate the hydrogen atoms the presence of three perchlorate counter ions in the crystal structure indicates the protonation state of the phosphate has not changed and two hydrogen atoms are still present (i. e. [(**L^1^**)_2_Zn_2_(H_2_PO_4_)]^3+^). Hydrogen bonding interactions supplement the Zn‐OPH_2_O_3_^−^ coordination with the ‐NH⋅⋅⋅O interactions from the ligand strand and the coordinated dihydrogen phosphate anion. The coordination and binding of phosphate induces a more twisted motif of the ligand strand, and the assembly could be considered more helical in nature. ESI‐MS analysis showed ions at *m/z* 1243 and 1143 corresponding to {[(**L^1^**)_2_Zn_2_(H_2_PO_4_)](ClO_4_)_2_}^+^ and {[(**L^1^**)_2_Zn_2_(HPO_4_)](ClO_4_)}^+^ respectively.

**Figure 4:**
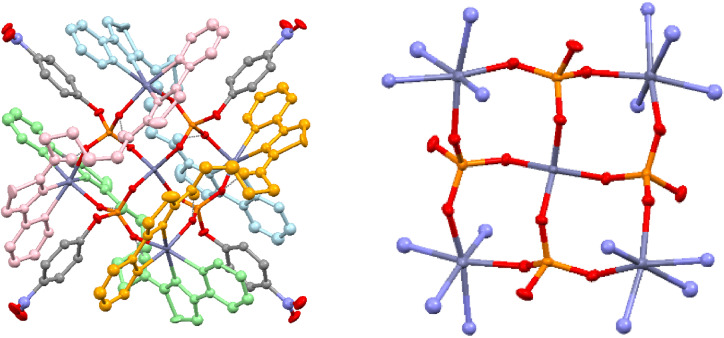
X‐ray structure of [(**L^1^**)_4_Zn_5_(NPP)_4_]^2+^. Thermal ellipsoids shown at the 50 % probability level. Selected anions omitted ligands colored for clarity. Color code: dark blue, Zn(II); red, O; blue, N; grey, C; orange P.

In a similar fashion **L^1^** was reacted with Zn^2+^ and half an equivalent of disodium 4‐nitrophenylphosphate (NPP^2−^) and crystals were deposited from an MeCN solution by slow diffusion of ethyl acetate. Analysis of the solid state by X‐ray diffraction showed a markedly different structure than previously observed with this species containing four ligands, four NPP dianions and five Zn^2+^ metal cations for example [(**L^1^**)_4_Zn_5_(NPP)_4_]^2+^ (Figure 4). In the crystal, each of the Zn^2+^ metal ions are coordinated by two bidentate domains for two separate ligands with the remaining bidentate *N*‐donor domains on each ligand coordinating different Zn^2+^cations. In addition to the four ligand *N*‐donor units each Zn^2+^ is also coordinated by two different *O*‐donor NPP^2−^ units giving a 6‐coordinate geometry. Each of the NPP^2−^ phosphate units bridge two metal ions coordinating each with a different oxygen atom from the phosphate unit. The remaining phosphate oxygen atom of the four NPP^2−^ units coordinates a central Zn^2+^ metal ion, with the resulting 4‐coordinate tetrahedral geometry of this ion differing from the 6‐coordinate peripheral metal cations. Coordination is also supplemented by hydrogen bonding interactions with both amine units on each ligand forming a −NH⋅⋅⋅O_3_PO− interaction. There is no real twist about the two ligand binding domains and the assembly can be considered a pentanuclear grid. It is interesting to note that it is perfectly feasible for the NPP^2−^ unit to coordinate in a similar manner to that observed with dihydrogen phosphate, giving a dinuclear structure for example [(**L^1^**)_2_Zn_2_(NPP)_2_]^2+^ and there are no steric or coordination constraints preventing this dinuclear structure from forming. Nor is it a consequence of the central tetrahedrally coordinated central Zn^2+^, as this motif could also be formed equally well with the phosphate anion. Rather we attribute this to π‐stacking between the phenyl unit of the *p*‐nitrophenylphosphate and the aromatic pyridyl‐thiazole binding domains. The sandwiching of the NPP unit in between the two aromatic binding domains is the likely driving force for the formation of the observed species.

Interestingly stoichiometry has little effect on the species produced as [(**L^1^**)_4_Zn_5_(NPP)_4_]^2+^ was obtained from reaction of 1 **L^1^**, 1Zn^2+^ and half an equivalent of NPP^2−^ and this ratio would favor the hypothetical dinuclear species [(**L^1^**)_2_Zn_2_(NPP)_2_]^2+^. Reaction of the correct stoichiometric ratio for example 4 **L^1^**, 5Zn^2+^ and 4NPP^2−^ results in an immediate precipitate and crystals can only be obtained when the incorrect stoichiometry is used (which results in slow deposition of a crystalline solid). Examination by ESI‐MS is problematic as samples produce a precipitate (presumably [(**L^1^**)_4_Zn_5_(NPP)_4_]^2+^) and analysis of the solution gave ions at *m/z* 582 and 1264 corresponding to {[(**L^1^**)_2_Zn_2_(NPP)]}^2+^ and {[(**L^1^**)_2_Zn_2_(NPP)](ClO_4_)}^+^ as these species are more soluble compared to the pentanuclear assembly.

**Figure 5:**
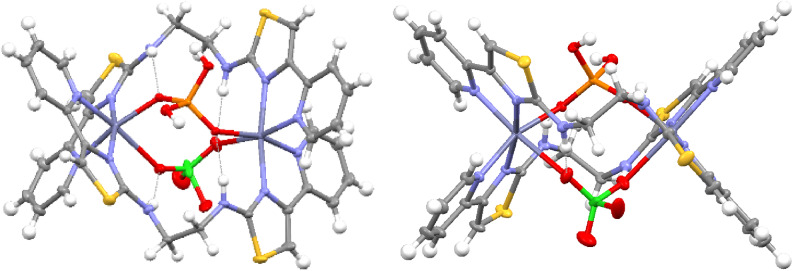
X‐ray structure of {[(**L^2^**)_2_Zn_2_(H_2_PO_4_)(ClO_4_)]^2+^. Thermal ellipsoids shown at the 50 % probability level. Selected anions omitted for clarity. Color code: dark blue, Zn(II); red, O; blue, N; green, Cl; yellow, S; grey, C; orange P.

The ligand **L^2^** is similar to **L^1^** but contains the shorter 1,2‐diaminoethane spacer unit. In a similar fashion to **L^1^**, **L^2^** reacts with Zn(ClO_4_)_2_ ⋅ 6H_2_O and whilst we were unable to obtain solid‐state data the ESI‐MS shows ions at *m/z* 1189 corresponding to {[(**L^2^**)_2_Zn_2_](ClO_4_)_3_}^+^ and a lower mass fragment at m/z 545 where the isotope pattern indicates the presence of both {[(**L^2^**)_2_Zn_2_](ClO_4_)_2_}^2+^ and {[(**L^2^**)Zn](ClO_4_)}^+^. Reaction of **L^2^**, Zn(ClO_4_)_2_ ⋅ 6H_2_O and half an equivalent of Bu_4_NH_2_PO_4_ in MeCN gave a pale yellow solution from which crystals were deposited upon slow diffusion of dichloromethane. Analysis of the X‐ray data showed a dinuclear mesocate structure with a dihydrogen phosphate anion bridging two metal ions in a similar fashion to the **L^1^**‐containing complex (Figure 5). However, each of the Zn^2+^ ions are 6‐coordinate formed by the four *N*‐donor ligand atoms and hydrogen phosphate and perchlorate anions as opposed to the 5‐coordinate metal centers seen in [(**L^1^**)_2_Zn_2_(H_2_PO_4_)]^3+^. Again, hydrogen bonding interactions are present with the ligand amine hydrogen bonding to both anions. Mass spectrometry data is consistent with ions observed at *m/z* 1187 and 1087 corresponding to {[(**L^2^**)_2_Zn_2_(H_2_PO_4_)(ClO_4_)_2_]}^+^ and {[(**L^2^**)_2_Zn_2_(HPO_4_)(ClO_4_)]}^+^ respectively.

**Figure 6:**
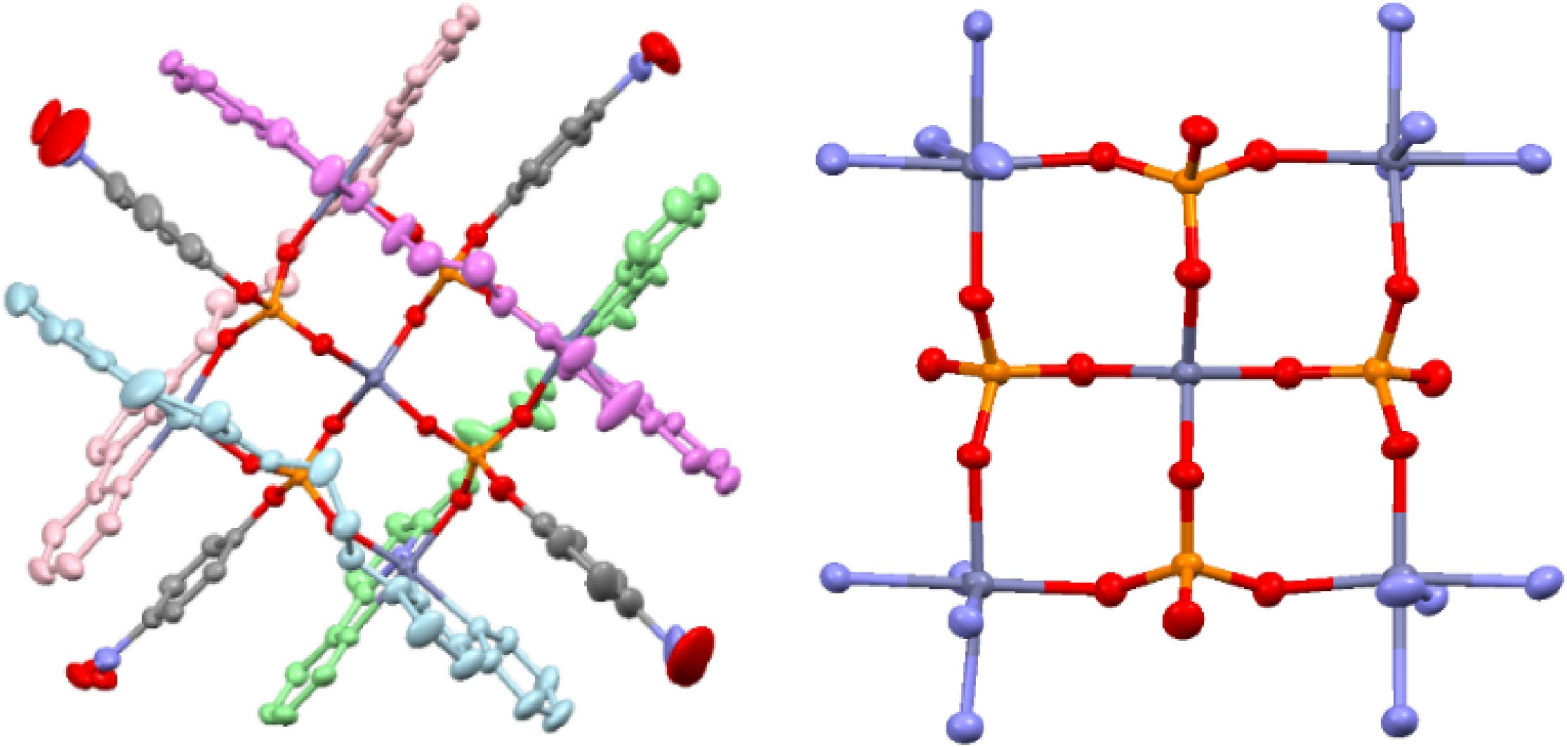
X‐ray structure of [(**L^1^**)_4_Zn_5_(NPP)_4_]^2+^. Thermal ellipsoids shown at the 50 % probability level. Selected anions omitted and ligands colored for clarity. Color code: dark blue, Zn(II); red, O; blue, N; grey, C; orange P.

Reaction of **L^2^**, Zn(ClO_4_)_2_ ⋅ 6H_2_O and half an equivalent of Na_2_NPP in water and MeCN gave a colorless solution from which a crystalline material was deposited upon the slow evaporation of the organic solvent. Analysis by X‐ray diffraction showed the formation of the pentanuclear species in an analogous fashion to **L^1^** for example [(**L^2^**)_4_Zn_5_(NPP)_4_]^2+^ (Figure 6). Again, the ligand coordinates different metal ions with the two bidentate *N*‐donor domains and the structure incorporates four NPP^2−^ anions and a central 4‐coordinate Zn^2+^ ion. The complex is more soluble than the pentanuclear **L^1^** derivative and this is observed in the ESI‐MS where an ion at *m/z* 1358 corresponding to {[(**L^2^**)_4_Zn_5_(NPP)_4_]}^2+^ is present, accompanied by ions at *m/z* 1208 and 554 corresponding to the lower molecular weight species {[(**L^2^**)_2_Zn_2_(NPP)](ClO_4_)}^+^ and {[(**L^2^**)_2_Zn_2_(NPP)]}^2+^ respectively.

Analysis of the complexes by ^1^H NMR (CD_3_CN/CD_3_OD) demonstrated the initial complexes [(**L^1/2^**)_2_Zn_2_]^4+^ show a number of species which we attribute to the racemic helicate and achiral mesocate species, but other species may involve coordination to different solvent molecules (e. g. [(**L^1^**)_2_Zn_2_(H_2_O)_2_(ClO_4_)_2_]^2+^) in solution. Addition of anions to these species does result in a change in the ^1^H NMR, indicative of anion binding. The NMR, solid‐state and ESI‐MS studies all show that ligands **L^1^** and **L^2^** both form dinuclear complexes (both with Zn^2+^ and Cu^2+^) and these can bind phosphate anions, and this can occur in competitive media such as MeOH and water.^[6]^

As the phenotypic approach to drug discovery requires the identification of ‘active’ compounds prior to target deconvolution, the complexes [(**L^1^**)_2_Zn_2_]^4+^, [(**L^2^**)_2_Zn_2_]^4+^, [(**L^1^**)_2_Cu_2_]^4+^ and [(**L^2^**)_2_Cu_2_]^4+^ plus their phosphate anion assemblies, were tested against cancer cells and non‐cancer cells in vitro.

**Figure 7:**
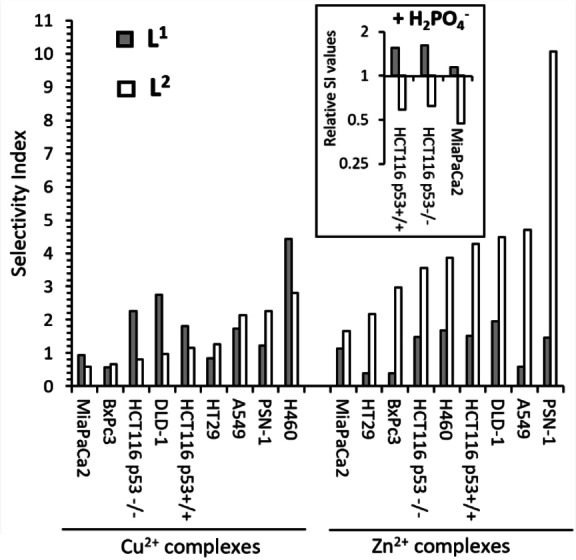
Selectivity indices (SI) for [(**L^1^**)_2_Cu_2_]^4+^, [(**L^2^**)_2_Cu_2_]^4+^, [(**L^1^**)_2_Zn_2_]^4+^ and [(**L^2^**)_2_Zn_2_]^4+^ against a panel of cancer cells. The Supporting Information is defined as the mean IC_50_ for non‐cancer ARPE‐19 cells divided by the mean IC_50_ for cancer cells (IC_50_±SD values are in Figure S2.1). The inset figure describes the impact of H_2_PO_4_^−^ on the Supporting Information of [(**L^1^**)_2_Zn_2_]^4+^ and of [(**L^2^**)_2_Zn_2_]^4+^, presented as the Supporting Information for the complex plus H_2_PO_4_^−^ relative to the complex alone; values above 1 demonstrating that the Supporting Information has increased in the presence of the anion and *vice versa*.

For [(**L^1^**)_2_Zn_2_]^4+^ and [(**L^1^**)_2_Cu_2_]^4+^, IC_50_ values ranged from 0.67 μM up to 6.12 μM across the cancer cell line panel (Figures S2.1 and S2.2). Whilst most of the cell lines showed similar chemosensitivity to both complexes, A549 cells were ~3x more sensitive to [(**L^1^**)_2_Cu_2_]^4+^ than [(**L^1^**)_2_Zn_2_]^4+^. Similar differences, but of a smaller magnitude, between the Cu^2+^ and Zn^2+^ complexes were observed in H460 and HT29 cells. The selectivity index (SI) values for [(**L^1^**)_2_Cu_2_]^4+^ and [(**L^1^**)_2_Zn_2_]^4+^ ranged from 4.42 to 0.39, with the Supporting Information for the Zn^2+^ **L^1^** complexes being lower for each cell line than for the Cu^2+^ **L^1^** complexes, except for MiaPaCa2 and PSN‐1 (Figure 7). The addition of H_2_PO_4_^−^ or PhOPO_3_^2−^ anions to [(**L^1^**)_2_Zn_2_]^4+^ prior to cell exposure decreased the activity of [(**L^1^**)_2_Zn_2_]^4+^ against ARPE‐19 non‐cancer cells whereas activity against cancer cell lines was less affected, resulting in an increase in the cancer selectivity (SI) of [(**L^1^**)_2_Zn_2_]^4+^ (Figure 7 inset; Figure S2.3).

[(**L^2^**)_2_Cu_2_]^4+^ was more active (lower IC_50_) against most of the cancer cell lines than [(**L^2^**)_2_Zn_2_]^4+^ (Figure S2.2). However, against ARPE‐19 non‐cancer cells, [(**L^2^**)_2_Zn_2_]^4+^ was less active (by ~6x) than [(**L^2^**)_2_Cu_2_]^4+^ resulting in substantially superior selectivity towards cancer cells (SI ranging from 10.08 against PSN‐1 to 1.65 against MiaPaCa2) (Figure 7). In contrast to [(**L^1^**)_2_Zn_2_]^4+^, incubation of [(**L^2^**)_2_Zn_2_]^4+^ with H_2_PO_4_^−^ failed to further improve the Supporting Information relative to [(**L^2^**)_2_Zn_2_]^4+^ alone (Figure 7, inset, Figure S2.3).

Mode of action studies in HCT116, PSN‐1 and ARPE‐19 cells re‐affirmed the chemosensitivity data showing that [(**L^1^**)_2_Zn_2_]^4+^ is more potent than [(**L^2^**)_2_Zn_2_]^4+^ but it is less cancer cell selective. Against HCT116 and PSN‐1 cancer cells, both [(**L^1^**)_2_Zn_2_]^4+^ and [(**L^2^**)_2_Zn_2_]^4+^ caused a decrease in cells in G1 phase of the cell cycle showing that cells were unable to adequately progress through the different cell cycle phases as part of cell proliferation and to re‐enter G1 (Figure S2.4, Table S2.1). This was concomitant with an increase in S phase cells suggesting partial S phase cell cycle arrest. In HCT116 cells, [(**L^2^**)_2_Zn_2_]^4+^ and [(**L^1^**)_2_Zn_2_]^4+^ increased the proportion of cells in G2/M ~2–3 fold, suggesting partial arrest at G2/M. [(**L^1^**)_2_Zn_2_]^4+^, and to a lesser extent [(**L^2^**)_2_Zn_2_]^4+^, also increased the subG1 population in HCT116 and PSN‐1 cells indicating cellular DNA fragmentation and cell death induction that was absent in control treated cells.

Against ARPE‐19 non‐cancer cells, [(**L^1^**)_2_Zn_2_]^4+^ similarly resulted in a decrease in G1 phase cells (from a mean of ~88 % to ~43 %, Table S2.1), with a concomitant ~5‐fold increase in the S phase subpopulation and a ~4‐fold increase in G2/M phase cells indicative of cell cycle arrest at S and G2/M. In contrast, the cell cycle profile for ARPE‐19 cells treated with [(**L^2^**)_2_Zn_2_]^4+^ was very similar to that of control treated ARPE‐19 cells, consistent with [(**L^2^**)_2_Zn_2_]^4+^ being more selective towards cancer cells than [(**L^1^**)_2_Zn_2_]^4+^_._

This was further confirmed by time lapse microscopy following the exposure of HCT116 and ARPE‐19 cells to [(**L^2^**)_2_Zn_2_]^4+^. Addition of the cell‐impermeable DNA binding dye propidium iodide (PI) enabled red fluorescent staining of any dead cells. In the HCT116 cancer cells, a clear increase in red‐fluorescing dead cells was evident with increased exposure time to [(**L^2^**)_2_Zn_2_]^4+^, consistent with the appearance of a sub‐G1 subpopulation in cell cycle analyses. In ARPE‐19 non‐cancer cells, however, there was no evidence of cell death induction in response to [(**L^2^**)_2_Zn_2_]^4+^ with brightfield images showing that cells were able to proliferate and reach near confluency by 48 h (Figure S2.5).

**Figure 8:**
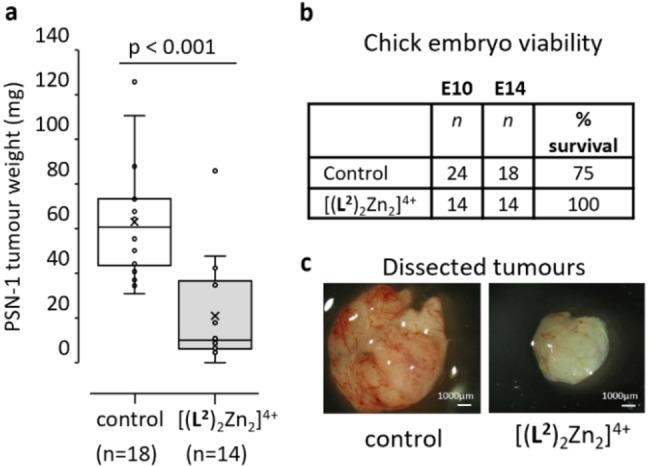
*In ovo* efficacy of [(**L^2^**)_2_Zn_2_]^4+^ towards pancreatic PSN‐1 tumors. a) Box plot of weights of PSN‐1 tumors that were formed *in ovo* and following 4 days treatment with 300 μM [(**L^2^**)_2_Zn_2_]^4+^ or solvent control. Mean (x); number of tumors per treatment group (n). Statistical significance of p<0.001, student's t‐test. b) Chick embryo survival from start of treatment with control or [(**L^2^**)_2_Zn_2_]^4+^ (embryonic day 10 [E10]) to the end of treatment (day 14, E14). c) Representative images indicating the typical relative size of PSN‐1 tumors dissected from underneath the chorioallantoic membrane of the developing chick embryo on day E14 for both control and [(**L^2^**)_2_Zn_2_]^4+^ treated tumors.

As the lead complex, [(**L^2^**)_2_Zn_2_]^4+^ was tested for in vivo efficacy against PSN‐1 human pancreatic tumors using the chick embryo model (*in ovo*).^[7]^ [(**L^2^**)_2_Zn_2_]^4+^ at 300 μM was well tolerated by the developing chick embryo with 100 % embryo survival at day 14. Importantly, [(**L^2^**)_2_Zn_2_]^4+^ induced a significant decrease in tumor weight (p<0.001, 67 % decrease in mean weight, Figure 8). Effects were corroborated by tumor volume measurements and histological analyses (Figure S2.6). The histology of the pancreatic tumors formed *in ovo* resembled that of pancreatic ductal adenocarcinomas of human patients with pancreatic ducts clearly visible (Figure S2.7). In contrast, tumors that had been treated with [(**L^2^**)_2_Zn_2_]^4+^ showed disruption of ducts and a more disorganized histological structure.

## Conclusions

The ligands **L^1^** and **L^2^** form self‐assemblies with Zn^2+^ and Cu^2+^ and can bind different anions. These show different selectivity profiles dependent upon which ligand, metal and anion is used. The results also provide evidence of *in ovo* efficacy at doses that are non‐toxic highlighting the therapeutic potential of [(**L^2^**)_2_Zn_2_]^4+^.

## Experimental Section

### Chemical Synthesis

**[(L^1^)_2_Zn_2_(ClO_4_)_2_](ClO_4_)_2_**_._ To a solution of Zn(ClO_4_)_2_ ⋅ 6H_2_O (9.1 mg, 0.025 mmol) in MeCN (1 mL) was added a suspension of ligand **L^1^** (10 mg, 0.025 mmol) in MeCN and the reaction gently warmed and sonicated until a clear solution had formed. Dichloromethane was slowly allowed to diffuse into the solution resulting in colorless block‐like crystals after several days. Filtration and washing with diethyl ether (1 mL) gave colorless crystals which lost solvent rapidly (yield=28 %).

**[(L^1^)_2_Zn_2_(H_2_PO_4_)](ClO_4_)_3_**. To a solution of Zn(ClO_4_)_2_ ⋅ 6H_2_O (9.1 mg, 0.025 mmol) in MeCN (1 mL) was added a suspension of ligand **L^1^** (10 mg, 0.025 mmol) in MeCN and the reaction gently warmed and sonicated until a clear solution had formed. To this was added a solution of Bu_4_NH_2_PO_4_ (3.9 mg, 0.012 mmol) in MeCN (0.5 mL) and dichloromethane was slowly allowed to diffuse into the solution resulting in the formation of colorless rod‐like crystals after several days. Filtration and washing diethyl ether (1 mL) gave lime green crystals which lost solvent rapidly (yield=39 %).

**[(L^1^)_4_Zn_5_(NPP)_5_](ClO_4_)_3_**. To a solution of Zn(ClO_4_)_2_ ⋅ 6H_2_O (9.1 mg, 0.025 mmol) in MeCN (1 mL) was added a suspension of ligand **L^1^** (10 mg, 0.025 mmol) in MeCN and the reaction gently warmed and sonicated until a clear solution had formed. To this was added a suspension of disodium 4‐nitrophenylphosphate (4.9 mg, 0.012 mmol) in MeCN (0.5 mL) and the rection warmed and sonicated until all the anion dissolved. Ethyl acetate was slowly allowed to diffuse into the solution resulting in the formation of pale‐yellow faceted prismatic crystals after several days. Filtration and washing diethyl ether (1 mL) gave pale‐yellow crystals which lost solvent rapidly (yield=42 %).

**[(L^2^)_2_Zn_2_(H_2_PO_4_)](ClO_4_)_3_**. To a solution of Zn(ClO_4_)_2_ ⋅ 6H_2_O (9.8 mg, 0.026 mmol) in MeCN (1 mL) was added a suspension of ligand **L^2^** (10 mg, 0.026 mmol) in MeCN and the reaction gently warmed and sonicated until a clear solution had formed. To this was added a solution of Bu_4_NH_2_PO_4_ (4.2 mg, 0.013 mmol) in MeCN (0.5 mL) and dichloromethane was slowly allowed to diffuse into the solution resulting in the formation of colorless plate‐like crystals after several days. Filtration and washing diethyl ether (1 mL) gave colorless crystals which lost solvent rapidly (yield=42 %).

**[(L^2^)_4_Zn_5_(NPP)_5_](ClO_4_)_3_**. To a solution of Zn(ClO_4_)_2_ ⋅ 6H_2_O (9.8 mg, 0.026 mmol) in MeCN (1 mL) was added a suspension of ligand **L^1^** (10 mg, 0.026 mmol) in MeCN and the reaction gently warmed and sonicated until a clear solution had formed. To this was added a solution of disodium 4‐nitrophenylphosphate (4.9 mg, 0.013 mmol) in H_2_O (1 mL) and the rection warmed and sonicated until all the anion dissolved. The solvent was then allowed to evaporate which deposited colorless plate‐like crystals after several days. Filtration and washing diethyl ether (1 mL) gave colorless crystals which lost solvent rapidly (yield=35 %).

**Crystallography**. Single crystal X‐ray diffraction data was collected at 150(2) K (100 K for [(**L^1^**)_4_Zn_5_(O_2_NC_6_H_4_PO_4_)_4_]^2+^) on a Bruker D8 Venture diffractometer equipped with a graphite monochromated Mo(K*α*) radiation source and a cold stream of N_2_ gas. Solutions were generated by conventional heavy atom Patterson or direct methods and refined by full‐matrix least squares on all *F* ^*2*^ data, using SHELXS‐97 and SHELXL software respectively.^[8]^ Absorption corrections were applied based on multiple and symmetry‐equivalent measurements using SADABS.^[9]^

[(**L^1^**)_2_Zn_2_(ClO_4_)_2_](ClO_4_)_2_. The structure contained positionally and rotationally disordered dichloromethane solvent molecules which were modelled over two positions using the *PART* instruction and one of the solvent carbon atoms was restrained using *ISOR*.

[(**L^1^**)_2_Zn_2_(H_2_PO_4_)_2_](ClO_4_)_3_. The structure contained positionally and substitutionally disordered dichloromethane molecules and these were modelled over two positions using the *PART* instruction. Two of the solvent molecules had their carbon chlorine bond lengths restrained using DFIX and some of the solvent molecules were restrained with *DELU*, *SIMU* and *ISOR*.

[(**L^1^**)_4_Zn_5_(O_2_NC_6_H_4_PO_4_)_4_]^2+^. Only a quarter of the molecule (comprising one ligand, one NPP dianion and one and one quarter Zn^2+^ atoms) was contained in the asymmetric cell and the remaining 3/4 of the assembly formed by symmetry operations. The structure contained whole molecule disorder with the ligand and NPP dianion modelled over two positions using the *PART* instruction. Due to the extensive disorder some bonds in the disordered ligand had to be constrained with *DFIX* instructions as well as *AFIX* 66 for the 6‐membered rings. Further to this *ISOR* was used globally for the carbon, nitrogen and oxygen atoms. The structure contained diffuse electron density, which despite attempts, could not be modelled and was removed using the solvent mask facility in Olex2.^[10]^ The solvent mask removed a total of 284 electrons per pentanuclear assembly which corresponds to 2 perchlorate anions 2 molecules of acetonitrile and 3 molecules of ethyl acetate. Despite removal of extensive number of solvent molecules with the use of the solvent mask function satisfactory data was obtained.

[(**L^2^**)_2_Zn_2_(H_2_PO_4_)_2_](ClO_4_)_3_. Only half of the helicate molecule was present in the asymmetric unit and the remaining half was formed by symmetry operations. The structure contained a rotationally disordered perchlorate molecule and the oxygen atoms were modelled in two positions using the *PART* instructions and constrained using *DELU*, *SIMU* and *ISOR*. These three restraints were also used on one of the dichloromethane solvent molecules.

[(**L^2^**)_4_Zn_5_(O_2_NC_6_H_4_PO_4_)_4_](ClO_4_)_2_. Unlike the **L^1^** derivative the whole molecule was present in the asymmetric unit cell. However, two of the ligands had disorder associated with the ethyl spacer unit and one of the thiazole rings and these were modelled in two positions using the *PART* instructions and constrained using *DELU*, *SIMU* and *ISOR*. Also present was solvent disorder that despite attempts could not be successfully modelled and as a result the diffuse electron density was removed using the solvent mask facility in Olex2, resulting in voids in the crystal structure.^[10]^ The solvent mask removed a total of 293 electrons in the asymmetric unit which corresponds to one perchlorate anion and eleven molecules of acetonitrile.

Deposition Number(s) 2287553 ([(**L** ^**1**^)_2_Zn_2_(ClO_4_)_2_](ClO_4_)_2_), 2287554 ([(**L** ^**1**^)_2_Zn_2_(H_2_PO_4_)_2_](ClO_4_)_3_), 2287563 ([(**L** ^**1**^)_4_Zn_5_ (O_2_NC_6_H_4_PO_4_)_4_]^2+^), 2287565 ([(**L** ^**2**^)_2_Zn_2_(H_2_PO_4_)_2_](ClO_4_)_3_), 2287566([(**L** ^**2**^)_4_Zn_5_(O_2_NC_6_H_4_PO_4_)_4_](ClO_4_)_2_) contains the supplementary crystallographic data for this paper. These data are provided free of charge by the joint Cambridge Crystallographic Data Centre and Fachinformationszentrum Karlsruhe Access Structures service.

**Cell lines and culture**. Cell lines were purchased from American Type Culture Collection (ATCC) except for p53^+/+^ and p53^−/−^ isogenic clones of HCT116 human colorectal adenocarcinoma cells which were a kind gift from Professor Bert Vogelstein.^[11]^ All cell lines were maintained at low passage and in antibiotic free media. HT29, DLD‐1, HCT116 p53^+/+^ and HCT116 p53^−/−^ are human colorectal carcinoma cell lines; PSN‐1, BxPC‐3 and MiaPaCa2 are human pancreatic cancer cell lines and A549 and H460 are human non‐small cell lung cancer cell lines. Cancer cell lines were cultured in the appropriate media as recommended by ATCC. The ARPE‐19 human retinal epithelial non‐cancer cell line was cultured in DMEM/F12 media (Gibco), 2 mM L‐glutamine, 1 mM sodium pyruvate and 10 % fetal bovine serum.

**Chemosensitivity studies**. The response of cells following a continuous 96 h exposure to test compounds was determined using the MTT assay. [(**L^1^**)_2_M_2_]^4+^ and [(**L^2^**)_2_M_2_]^4+^ complexes (M=metal ion, Zn^2+^ or Cu^2+^) were freshly formed by adding DMSO to pre‐weighed individual components and mixing by pipetting. The compounds were then further diluted in cell culture media with the final DMSO concentration that cells were exposed to being 0.1 % (vehicle control). All cell lines were seeded in 96 well plates at 2×10^3^ cells per well and incubated overnight at 37 °C. Following incubation, media was removed and replaced with fresh media containing test compounds. Cells were further incubated for 96 h with media then removed and replaced with fresh media (200 μl/well) containing MTT solution at a final concentration of 0.5 mg/mL. Cells were then incubated for a further 4 h. Media and MTT were then removed, and formazan crystals that had formed were dissolved in 150 μL of DMSO and well absorbance measured at 540 nm. The concentration of test compound required to reduce cell growth by 50 % (IC_50_) was determined from dose response curves. Potency was recorded as the IC_50_±standard deviation for three independent experiments. The selectivity index (SI) was defined as the ratio of the mean IC_50_ values for non‐cancer to cancer cell lines with values >1 representing test compound selectivity against cancer cells.

**Cell cycle studies**. For cell cycle studies, HCT116 and PSN‐1 cancer cells and ARPE‐19 non‐cancer cells were seeded in 25 cm^2^ cell culture flasks and 24 h post seeding, media was replaced with fresh complete media containing 0.015 % DMSO (solvent control), 15 μM [(**L^1^**)_2_Zn_2_]^4+^ or 15 μM [(**L^2^**)_2_Zn_2_]^4+^. At 72 h, cells were collected from treated flasks, gently permeabilized (solution 10, Chemometec), and incubated with 10μg/ml DAPI for 5 min at 37 ^°C^ before analyzing cell DNA content (emission detection at 450 nm for double stranded DNA) by image cytometry (NC3000 image cytometer, Chemometec).

**Time lapse microscopy**. Real time monitoring and microscopic imaging of cells was performed using Axion BioSystems Lux FL microscopes placed directly inside a cell culture incubator. This enabled the effects of [(**L^2^**)_2_Zn_2_]^4+^ on the growth and survival of HCT116 cancer cells and ARPE‐19 non‐cancer cells to be directly monitored over time within the same magnification field of view. Cells were seeded in 25 cm^2^ cell culture flasks and 24 h post seeding, fresh complete media containing solvent control or 15 μM [(**L^2^**)_2_Zn_2_]^4+^ was added. 3 h following the addition of 15 μM [(**L^2^**)_2_Zn_2_]^4+^ or DMSO solvent control, cell impermeable DNA binding dye propidium iodide (PI) was added to the media at a final concentration of 4 μg/mL for the positive staining of dead or dying cells. Lux FL microscopes were focused on a particular field of view and programmed to acquire images every 30 min for 48 h in brightfield (to monitor cell proliferation and changes in cell confluency) and in the red fluorescent channel (to detect PI positive staining and monitor cell death induction).

***In ovo*** **efficacy studies**. *In ovo* experiments using the chick embryo model were performed in accordance with UK legislation and ethical guidelines with all embryos being humanely terminated on day 14 of chick embryonic development (E14) as stipulated by the UK Animals Scientific Procedures Act 1986 (amended 2012). E14 represents two‐thirds of the chick embryo gestation period and up to, and including, day E14 the chick embryo is classified as a non‐protected, 3Rs compliant model with no animal license or home office approval being required.

Fertilised Shaver Brown hen eggs were purchased from Medeggs Ltd. To commence embryonic development (day E0), eggs were incubated on their side in egg trays at 37.8 °C and 45 % humidity in a specialized poultry egg incubator (Brinsea OvaEasy 380) for 3 days (with the upwards side of the egg labelled in pencil to orientate for E3 windowing). Incubator shelves were set to alternately tilt 45° from a horizontal position every 45 min. On day 3 (E3), the wide base of each egg was gently pierced and 7 mL of albumen were removed to lower the chorioallantoic membrane (CAM) from the eggshell before windowing. Eggs were windowed by piercing a small hole on the labelled side of the egg, applying a 3 cm piece of invisible tape over the eggshell area to be windowed, and cutting using fine scissors a small three sided window (~2 cm by ~1 cm by ~2 cm) in the eggshell.^[12]^ Windowed eggs (E3) were further incubated without tilting at 37.8 °C and 45 % humidity until day E7.

On day E7, eggs containing viable embryos were implanted with tumor cells. PSN‐1 pancreatic cancer cells growing as monolayers and in logarithmic phase growth were harvested from cell culture flasks by standard trypsinization, counted, pelleted at 200 g for 5 min and resuspended in a small volume of sterile PBS to generate a concentrated cell slurry. For efficient engraftment of tumor cells, a small area of the highly vascularized chorioallantoic membrane (CAM) was dried with sterile gauze onto which a 4 mm silicone ring was carefully placed. 4 x10^6^ PSN‐1 cells were pipetted inside the ring and onto the CAM and eggs were further incubated at 37.8^0^C and 45 % humidity for 3 days. On day E10, viable eggs were treated with 300 μM [(**L^1^**)_2_Zn_2_]^4+^ or 0.3 % DMSO solvent control by carefully pipetting 25 uL into the silicone ring and eggs were then returned to 37.8 °C and 45 % humidity until embryo termination on day E14.

On day E14, treated embryos were scored as alive or dead and tumors were excised, with PSN‐1 tumors typically developing underneath the CAM. Excised tumors were placed in a petri dish in sterile PBS and imaged using a Zeiss SteREO Discovery V12 stereomicroscope equipped with an Axiocam 305 camera (Zeiss). Any excess CAM tissue attached to the excised tumor was cut away with dissection scissors and tweezers. Excess PBS was removed and tumors were weighed using a precision balance on small cut pieces of parafilm as described.^[13]^ Tumor volumes, as an additional endpoint measurement to tumor weight determinations, were calculated from microscopy images of excised tumors. Tumor volumes were estimated assuming an ellipsoidal shape using the formula, volume=4/3π×length/2×width/2×height/2, where height is estimated as 2/3×length, as previously described,[^14^, ^15^, ^16^] with measurements determined from acquired images using Image J.

**Histological analysis**. Weighed tumors were fixed in 10 % neutral buffered formalin for 48 h at room temperature. Samples were tissue processed using an automated Leica ASP200S tissue processor, embedded in paraffin wax (Leica 11150 wax embedding station) and sectioned at 5 μm using a rotary microtome (Leica RM2255). Microscope slides of mounted sections were deparaffinized using xylene (2×5 min), rehydrated through graded ethanol incubations before a 2 minute wash in water and staining with haematoxylin (Thermo Scientific 6765009) for 3 min. After differentiation with 10 % glacial acetic acid and bluing with tap water by standard histological procedures, sections were then stained with eosin (0.5 % solution in water, Carl Roth). Haematoxylin and eosin‐stained sections were then viewed using a Keyence VHX‐6000 digital microscope.

## Supporting Information

The Supporting Information contains extensive further experimental, spectroscopic and biological details.

## Conflict of interest

The authors declare no conflict of interest.

1

## Supporting information

As a service to our authors and readers, this journal provides supporting information supplied by the authors. Such materials are peer reviewed and may be re‐organized for online delivery, but are not copy‐edited or typeset. Technical support issues arising from supporting information (other than missing files) should be addressed to the authors.

Supporting Information

## Data Availability

The data that support the findings of this study are available from the corresponding author upon reasonable request.
