# Supplementary material for: Preclinical Evaluation of Zn(II) Self‐Assemblies with Selective Cytotoxic Activity Against Cancer Cells In Vitro and In Ovo
Source: Chemistry. 2023 Nov 21;30(3):e202302803. doi: 10.1002/chem.202302803 (PMC10952438; doi:10.1002/chem.202302803)
Supplement: Supplementary file 1 — Supporting Information [file CHEM-30-0-s001.pdf]

Chemistry–A European Journal

Supporting Information

Preclinical Evaluation of Zn(II) Self-Assemblies with Selective Cytotoxic Activity Against Cancer Cells In Vitro and *In Ovo*

Simon J. Allison, Gage P. Ashton, Hannah J. Lynch, Bethany R. Shire, Roger M. Phillips, Gareth M. B. Parkes, Emma Pinder, Craig R. Rice,* Ana A. M. Teixeira, Tibo Volleman, and Daisy A. Wordsworth

A. Chemistry Studies

General Information

Chemicals were purchased and used without further purification. ^{1}H and ^{13}C NMR spectra were recorded on a 400MHz Bruker Avance DP X400. Mass spectra were obtained on an Agilent 6210 TOF MS for the organic species with the metal complexes run on a Bruker MicroQTOF LC. CAUTION: perchlorate salts are potentially explosive and should be treated with due care. Those complexes described below which were isolated as perchlorates were only prepared in small amounts (5 – 10 mg) and we had no problems with them.

Ligand Synthesis.[1]

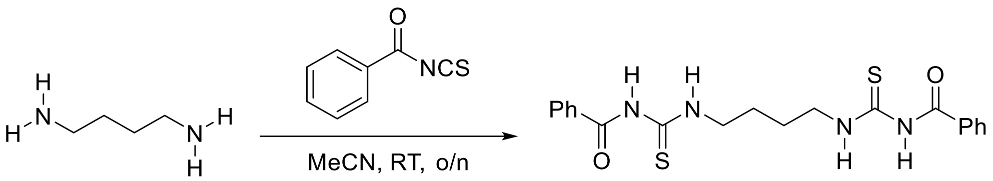

To a 100 mL RBF charged with putrescine (0.35 g, 3.9 mmol) and MeCN (25 mL) was added a solution of benzoyl isothiocyanate (1.3 mL, 1.59 g, 9.8 mmol) in MeCN (25 mL) slowly over 30 min with constant stirring. The reaction mixture was then stirred at RT for a further 12 h during this time a heavy white precipitate was formed. The resulting mixture was the added dropwise, whilst stirring, to deionised water (100 mL) to yield a cream precipitate which was then isolated via vacuum filtration. The solid was then suspended in MeOH (20 mL) in a 50 mL conical flask and sonicated (5 min). The cream suspension was then once again filtered under vacuum to yield a fine white solid which was washed with further portions of MeOH (3 × 5 mL) to give the dibenzoylated thiourea as a fine white powder (0.90 g, 55%). ^{1}H NMR (400 MHz, $(CD_3)_2SO$) δ (ppm) 11.30 (s, 2H, PhCON*H*), 10.92 (t, *J* = 5.4, 2H, $-CH_2NH-$), 7.92 (d, *J* = 7.2, 4H, H-2 Ph), 7.64 (t, *J* = 7.4, 2H, H-4 Ph), 7.51 (t, *J* = 7.8, 4H, H-3 Ph), 3.68 (d, *J* = 5.4 Hz, 4H, $-CH_2NH-$), 1.72 (brs, 4H, $-CH_2CH_2NH-$). ^{13}C NMR [400 MHz, $(CD_3)_2SO$]: δ_C = 183.5 (C=S), 168.4 (C=O), 133.4 (CH), 132.7 (Q), 129.0 (CH), 128.7 (CH), 44.8 (CH_2), 25.6 (CH_2). ESI-MS *m/z* 415 ($M + H^+$), HR ESI-MS found 415.1257 $C_{20}H_{23}N_4S_2O_2$ requires 415.1268 (error 2.65 ppm).Whilst the ESI-MS analysis did give an accurate ion the compound did decompose rapidly in the ESI MS process and only a small M^+ ion was observed. This behaviour is common to all of the aliphatic bridged dithioamines and their derivatives.

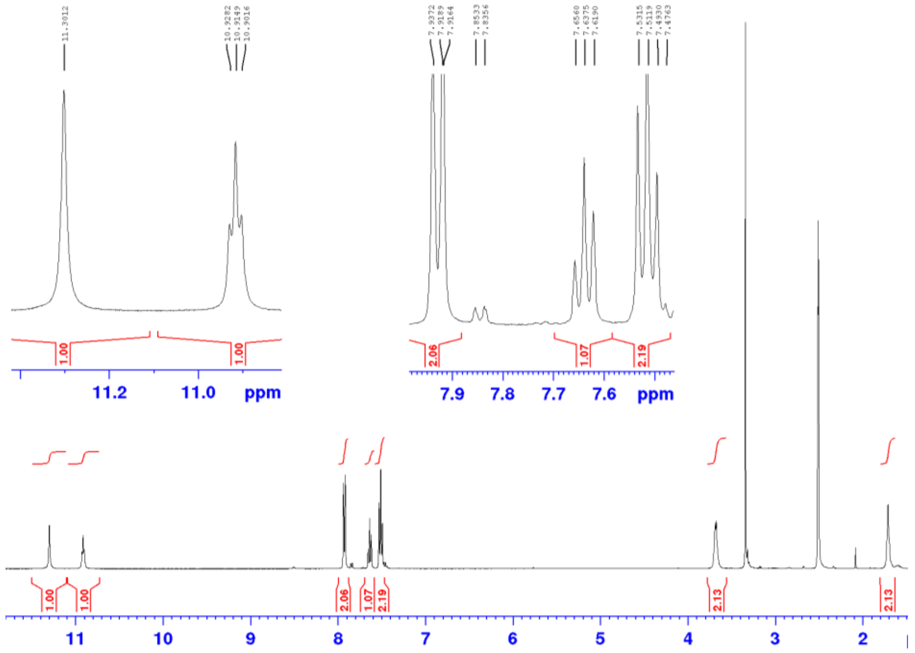

Figure S1.1. ^{1}H NMR ($(CD_3)_2SO$) of the dibenzoylated dithiourea derivative.

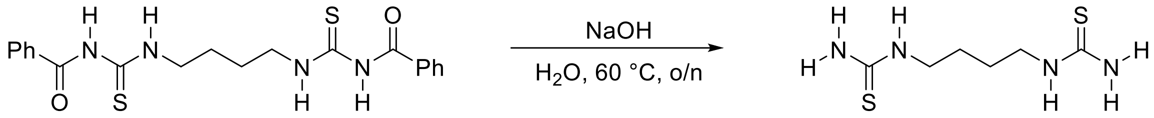

In a 50 mL RBF was combined the dibenzoylated dithiourea (510 mg, 1.2 mmol), deionised water (10 mL) and a magnetic stir bar. A solution of NaOH (290 mg, 7.4 mmol) in H_2O (10 mL) was then added to the reaction mixture whilst stirring at 60 °C. After 12 h the resulting colourless solution was then cooled to RT during which time a fine white precipitate was formed. The precipitate was then collected via vacuum filtration and then washed with deionised water (3 × 3 mL). To give the dithiourea as a fine white powder (210 mg, 83%). The ^{1}H NMR gives three broad signals in the aromatic region and two broad signals at 3.0 and 1.4 ppm. As has been mentioned previously is suspected that intra-molecular hydrogen bonding is inducing broad peaks in the ^{1}H NMR. However, the ^{13}C and ESI-MS is exactly as expected. ^{13}C NMR [400 MHz, DMSO-*d*^{6}]: δ_C = 183.5 (C=S), 44.1 (CH_2), 26.9 (CH_2). ESI-MS *m/z* 207 (M + H^+). HR ESI-MS found 207.0735 $C_6H_{15}N_4S_2$ requires 207.0733 (error 1.48 ppm).

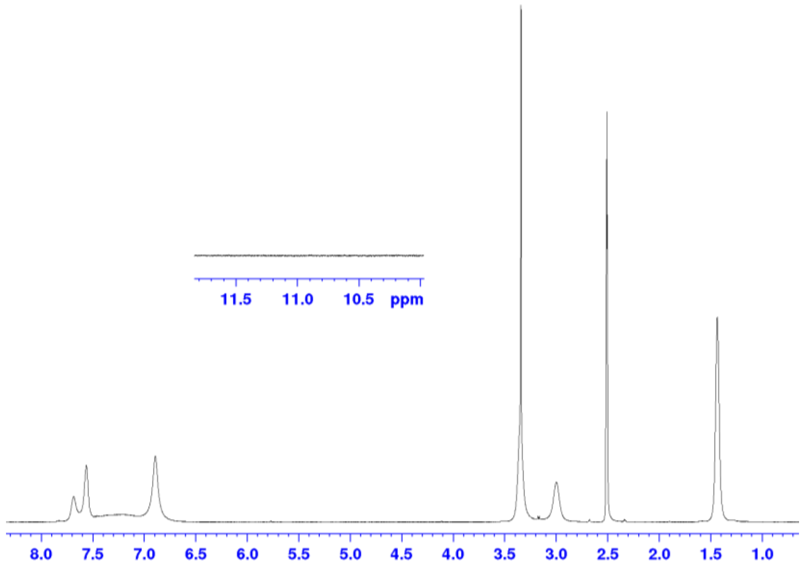

Figure S1.2. ^{1}H NMR ($(CD_3)_2SO$) of the dithiourea derivative.

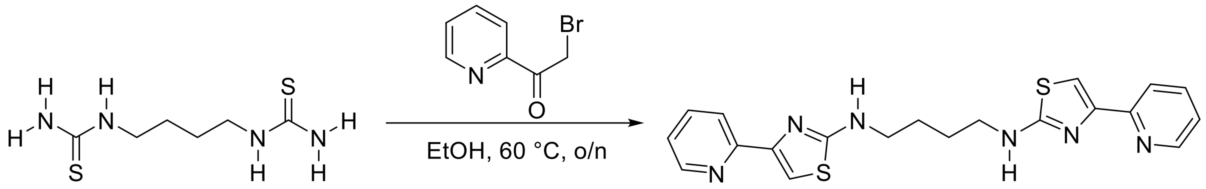

Synthesis of L[1]. A 25 mL RBF was charged with the dithiourea derivative (150 mg, 0.73 mmol), EtOH (10 mL) and a magnetic stir bar and the mixture was then heated to 60 °C and to this added α-bromoacetyl pyridine (0.30 mg, 1.5 mmol) in a solution in EtOH (2 mL) whilst stirring. Heating was continued for a further 8 h during which time a heavy yellow precipitate was formed. This was then isolated via vacuum filtration and the filtrand was then washed with portions of EtOH (3 × 2 mL) to give the protonated product. The free-base ligand was then isolated by suspending in concentrated ammonia (15 mL) for 24 h. The resulting colourless suspension was then sonicated (5 min) and was filtered under vacuum and the filtrand washed with portions of deionised water (5 × 2 mL) and EtOH (2 × 2 mL), giving the product as a fine off-white powder (180 mg 60%). ^{1}H NMR (400 MHz, $(CD_3)_2SO$) δ (ppm) 8.53 (d, *J* = 4, 2H, H-6 py), 7.88 (d, *J* = 7.8, 2H, H-3 py), 7.79 (dt, 7.6, *J* = 1.7, 2H, H-4 py), 7.75 (t, *J* = 5.4, 2H, -N*H*), 7.28 (s, 2H, tz), 7.25 (dd, *J* = 5.7 Hz, 2H, H-5 py), 3.33 (brs, 4H, -CH_2NH-, overlapping with H_2O), 1.71 (brs, 4H, -CH_2CH_2NH-). ^{13}C NMR (400 MHz $(CD_3)_2SO$) δ 169.2, 153.0, 150.7, 149.7, 137.5, 122.8, 120.7, 104.9, 44.6 and 26.7 ppm. ESI-MS *m/z* 409 (M + H^+), HR ESI-MS found 409.1261 $C_{20}H_{21}N_6S_2$ requires 409.1264 (error 0.97 ppm). IR (ATR) υ/cm^{-1} 3216 (m, -NH), 2870 (w, CH), 1574 (s, N=CH) and 1516 (w, CH).

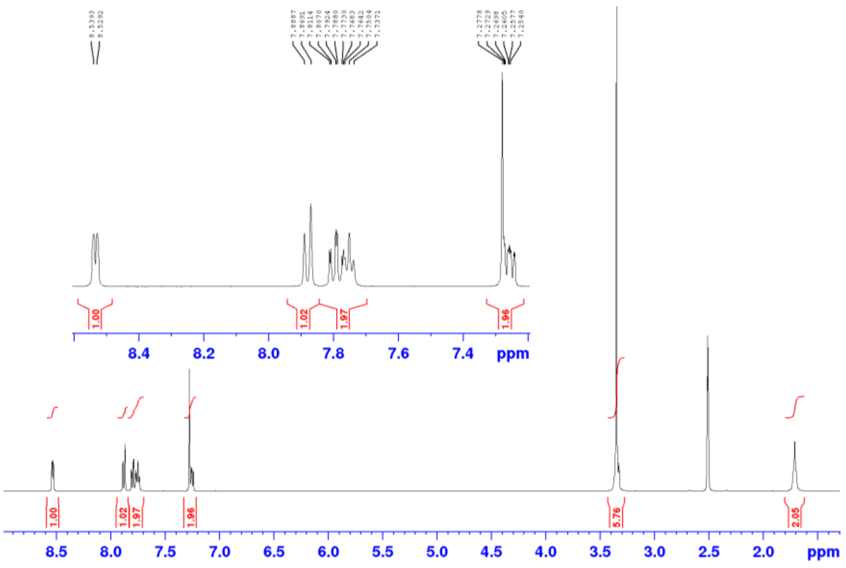

Figure S1.3. ^{1}H NMR ($(CD_3)_2SO$) of ligand **L^1**.

Synthesis of L^2

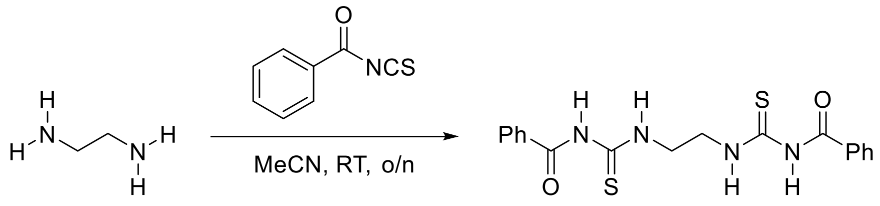

To a 250 mL RBF was charged with ethylenediamine (1 mL, 0.9 g, 15 mmol) and MeCN (50 mL) was added a solution of benzoyl isothiocyanate (5 mL, 6.1 g, 37 mmol) in MeCN (50 mL) slowly over 30 min with constant stirring. The reaction mixture was then stirred at RT for a further 12 h during this time a heavy white precipitate was formed. The resulting mixture was the added dropwise whilst stirring to deionised water (300 mL) to yield a white/yellow precipitate which was then isolated via vacuum filtration. The solid was then suspended in MeOH (50 mL) in a 100 mL conical flask and sonicated (5 min). The white/yellow suspension was then once again filtered under vacuum to yield a fine white solid which was washed with further portions of MeOH (3 × 10 mL) to give the dibenzoylated dithiourea as a fine white powder (3.5 g, 60%). ^{1}H NMR (400 MHz, $(CD_3)_2SO$) δ (ppm) 11.39 (s, 2H, PhCON*H*), 10.97 (s, 2H, -CH_2CON*H*), 7.95 (d, *J* = 7.5, 4H, H-2 Ph), 7.63 (t, *J* = 7.5, 2H, H-4 Ph), 7.51 (t, *J* = 7.5 Hz, 4H, H-3 Ph), 4.04 (s, 4H, -CH_2-). ^{13}C NMR [400 MHz, $(CD_3)_2SO$]: δ_C = 183.5 (C=S), 168.4 (C=O), 133.4 (CH), 132.7 (Q), 129.0 (CH), 128.7 (CH), 44.8 (CH_2). ESI-MS *m/z* 387 (M + H^+), HR ESI-MS found 387.0927 $C_{18}H_{19}N_4S_2O_2$ requires 387.0944 (error 4.5 ppm). Whilst the ESI-MS analysis did give an accurate ion the compound did decompose rapidly in the ESI MS process and only a small M^+ ion was observed. This behaviour is common to all the aliphatic bridged dithioamines and their derivatives.

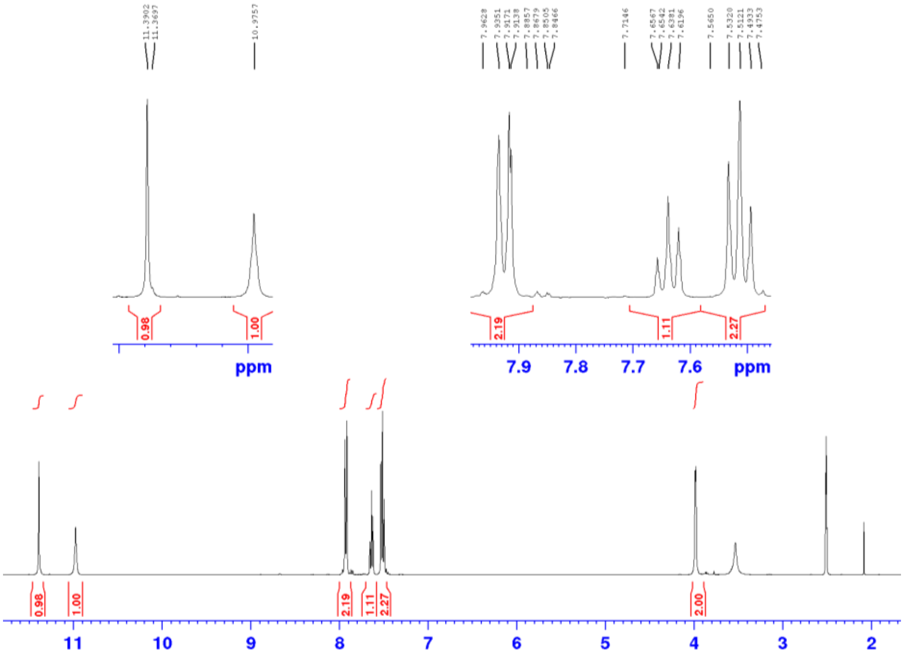

Figure S1.4. ^1H NMR ($(CD_3)_2SO$) of dibenzoylated dithiourea derivative.

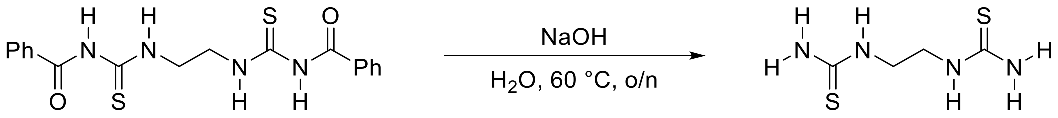

In a 100 mL RBF was combined the benzoylated dithiourea (1 g, 2.6 mmol), deionised water (30 mL) and a magnetic stir bar. A solution of NaOH (0.63 g, 15.8 mmol) in H_2O (15 mL) was then added to the reaction mixture whilst stirring at 60 °C. After 12 h the resulting colourless solution was then cooled to RT during which time a fine white precipitate was formed. The precipitate was then collected via vacuum filtration and then washed with deionised water (3 × 5 mL) giving the thiourea as a fine white powder (0.43 g, 93%). The ^1H NMR gives four broad signals in the aromatic region and two broad signals at ~ 3.5 ppm. It is suspected that intra-molecular hydrogen bonding is inducing broad peaks in the ^1H NMR. However, both the ^{13}C and ESI-MS are exactly as expected. ^{13}C NMR [400 MHz, $(CD_3)_2SO$]: δ_C = 183.9 (C=S), 43.7 (CH_2). ESI-MS *m/z* 179 (M + H^+). HR ESI-MS found 179.0415 $C_4H_{11}N_4S_2$ requires 179.042 (error 2.61 ppm).

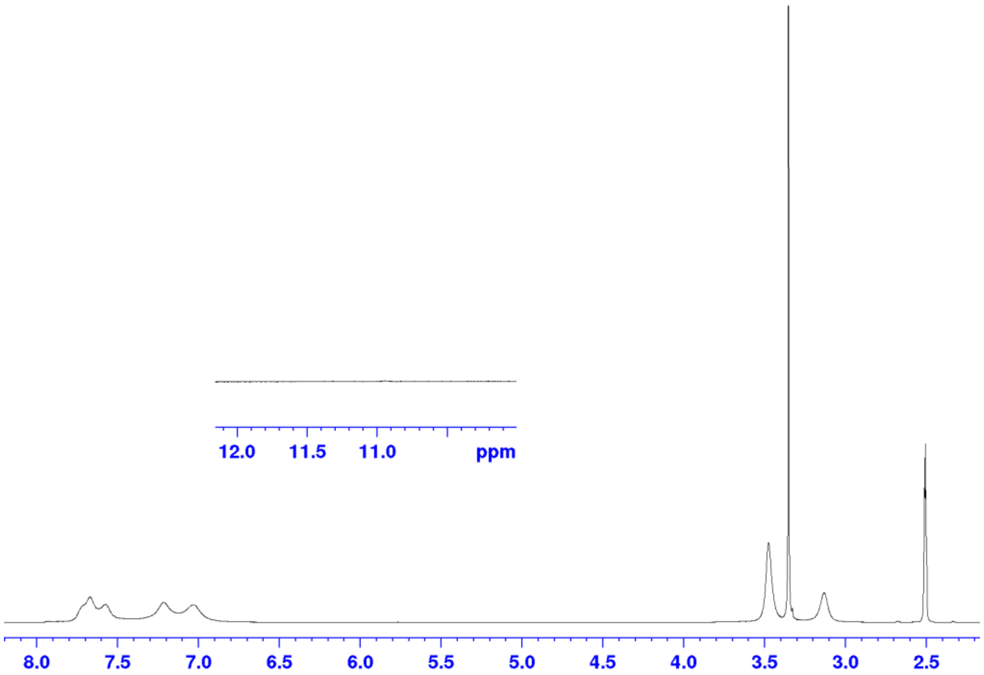

Figure S1.5. ^{1}H NMR ($(CD_3)_2SO$) of the dithiourea derivative.

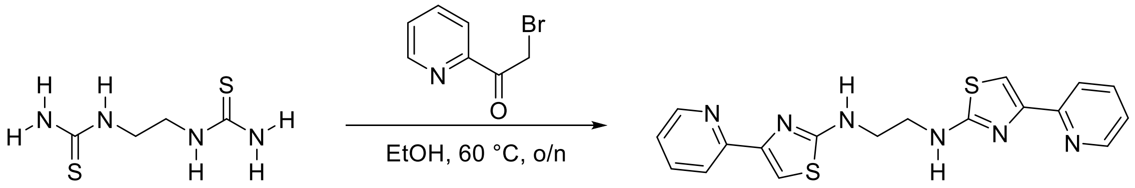

Synthesis of L^2. A 25 mL RBF was charged with dithiourea derivative (228 mg, 1.3 mmol), EtOH (10 mL) and magnetic stir bar and the mixture was then heated to 60 °C and to this added α-bromoacetyl pyridine (540 mg, 2.7 mmol) in a solution in EtOH (2 mL) solution whilst stirring. Heating was continued for a further 8 h during which time a heavy yellow precipitate was formed. This was then isolated via vacuum filtration and the filtrand was then washed with portions of EtOH (3 × 2 mL) to give the protonated product. The free-base ligand was then isolated by suspending in concentrated ammonia (15 mL) for 24 h. The resulting colourless suspension was then sonicated (5 min) and was filtered under vacco and the filtrand washed with portions of deionised water (5 × 2 mL) and EtOH (2 × 2 mL) giving the product as a fine off-white powder (402 mg 81%). ^{1}H NMR (400 MHz, $(CD_3)_2SO$) δ (ppm) 8.55 (d, *J* = 4, 2H, H-6 py), 7.91- 7.89 (m, overlapping, 4H, H-3 py and -N*H*), 7.79 (dt, *J* = 7.6, 1.6, 2H, H-4 py), 7.31 (s, 2H, tz), 7.26 (ddd, *J* = 7.4, 2.6, 1.2, 2H, H-5 py), 3.60 (d, *J* = 2.5 Hz, 4H, -C*H*$_2$-). ^{13}C NMR (100 MHz, $(CD_3)_2SO$) δ 169.0, 152.9, 150.7, 149.7, 137.4, 122.8, 120.8, 105.4 and 44.1 ppm. ESI-MS *m/z* 381 (M + H$^+$). HR ESI-MS found 381.0934 $C_{18}H_{17}N_6S_2$ requires 381.0951 (error 4.79 ppm). IR (ATR) υ/cm^{-1} 3206 (s, -NH), 3001 (s, CH), 1590 (s, N=CH) and 1531 (s, CH).

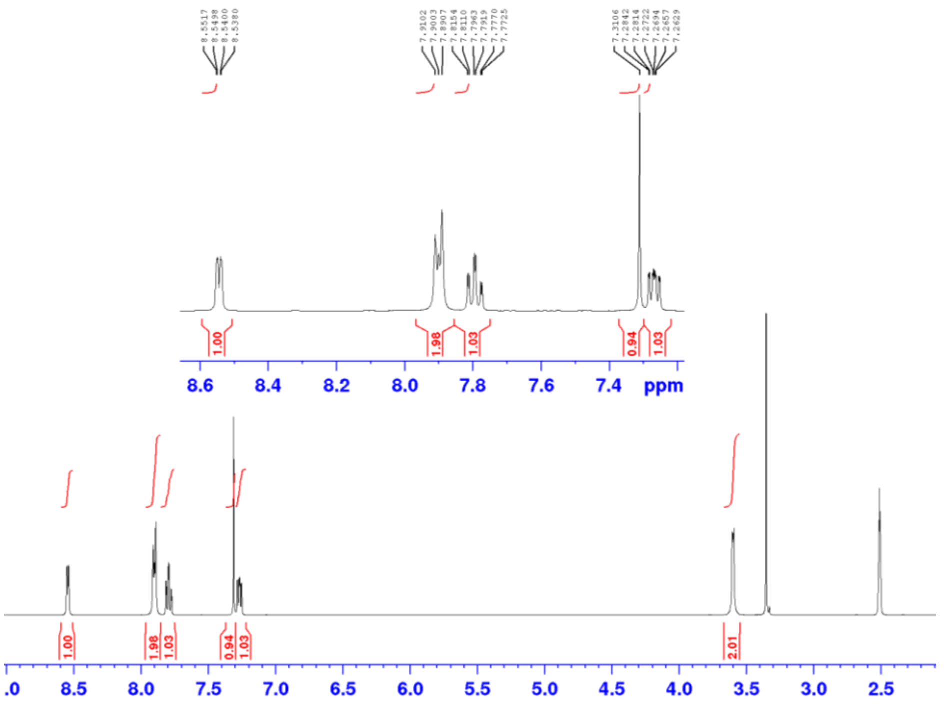

Figure S1.6. ^{1}H NMR ($(CD_3)_2SO$) of ligand **L²**.

Complex Synthesis.

Synthesis of $[(L^1)_2Zn_2(ClO_4)_2](ClO_4)_2$. To a solution of $Zn(ClO_4)_2{\cdot}6H_2O$ (9.1 mg, 0.025 mmol) in MeCN (1 ml) was added a suspension of ligand **L¹** (10 mg, 0.025 mmol) in MeCN and the reaction gently warmed and sonicated until a clear solution had formed. Dichloromethane was slowly allowed to diffuse into the solution resulting in colourless block-like crystals after several days. Filtration and washing with Et_2O (1 ml) and diethyl ether (1 ml) gave colourless crystals which lost solvent rapidly (yield = 28%).

Synthesis of $[(L^1)_2Zn_2(H_2PO_4)](ClO_4)_3$. To a solution of $Zn(ClO_4)_2{\cdot}6H_2O$ (9.1 mg, 0.025 mmol) in MeCN (1 ml) was added a suspension of ligand **L¹** (10 mg, 0.025 mmol) in MeCN and the reaction gently warmed and sonicated until a clear solution had formed. To this was added a solution of $Bu_4NH_2PO_4$ (3.9 mg, 0.012 mmol) in MeCN (0.5 ml) and dichloromethane was slowly allowed to diffuse into the solution resulting in the formation of colourless rod-like crystals after several days. Filtration and washing diethyl ether (1 ml) gave lime green crystals which lost solvent rapidly (yield = 39%).

Synthesis of $[(L^1)_4Zn_5(NPP)_5](ClO_4)_3$. To a solution of $Zn(ClO_4)_2{\cdot}6H_2O$ (9.1 mg, 0.025 mmol) in MeCN (1 ml) was added a suspension of ligand **L¹** (10 mg, 0.025 mmol) in MeCN and the reaction gently warmed and sonicated until a clear solution had formed. To this was added a suspension of disodium 4-nitrophenylphosphate (4.9 mg, 0.012 mmol) in MeCN (0.5 ml) and the rection warmed and sonicated until all the anion dissolved. Ethyl acetate was slowly allowed to diffuse into the solution

resulting in the formation of pale-yellow faceted prismatic crystals after several days. Filtration and washing diethyl ether (1 ml) gave pale-yellow crystals which lost solvent rapidly (yield = 42%).

Synthesis of $[(L^2)_2Zn_2(H_2PO_4)](ClO_4)_3$. To a solution of $Zn(ClO_4)_2{\cdot}6H_2O$ (9.8 mg, 0.026 mmol) in MeCN (1 ml) was added a suspension of ligand $\mathbf{L^2}$ (10 mg, 0.026 mmol) in MeCN and the reaction gently warmed and sonicated until a clear solution had formed. To this was added a solution of $Bu_4NH_2PO_4$ (4.2 mg, 0.013 mmol) in MeCN (0.5 ml) and dichloromethane was slowly allowed to diffuse into the solution resulting in the formation of colourless plate-like crystals after several days. Filtration and washing diethyl ether (1 ml) gave colourless crystals which lost solvent rapidly (yield = 42%).

Synthesis of $[(L^2)_4Zn_5(NPP)_5](ClO_4)_3$. To a solution of $Zn(ClO_4)_2{\cdot}6H_2O$ (9.8 mg, 0.026 mmol) in MeCN (1 ml) was added a suspension of ligand $\mathbf{L^1}$ (10 mg, 0.026 mmol) in MeCN and the reaction gently warmed and sonicated until a clear solution had formed. To this was added a solution of disodium 4-nitrophenylphosphate (4.9 mg, 0.013 mmol) in H_2O (1 ml) and the rection warmed and sonicated until all the anion dissolved. The solvent was then allowed to evaporate which deposited colourless plate-like crystals after several days. Filtration and washing diethyl ether (1 ml) gave colourless crystals which lost solvent rapidly (yield = 35%).

Crystallography

Single crystal X-ray diffraction data was collected at 150(2) K (100 K for $[(L^1)_4Zn_5(O_2NC_6H_4PO_4)_4]^{2+}$) on a Bruker D8 Venture diffractometer equipped with a graphite monochromated Mo(Kα) radiation source and a cold stream of N_2 gas. Solutions were generated by conventional heavy atom Patterson or direct methods and refined by full-matrix least squares on all F^2 data, using SHELXS-97 and SHELXL software respectively.[2] Absorption corrections were applied based on multiple and symmetry-equivalent measurements using SADABS.[3]

$[(\mathbf{L^1})_2Zn_2(ClO_4)_2](ClO_4)_2$. The structure contained positionally and rotationally disordered dichloromethane solvent molecules which were modelled over two positions using the *PART* instruction and one of the solvent carbon atoms was restrained using *ISOR*.

$[(\mathbf{L^1})_2Zn_2(H_2PO_4)_2](ClO_4)_3$. The structure contained positionally and substitutionally disordered dichloromethane molecules and these were modelled over two positions using the *PART* instruction. Two of the solvent molecules had their carbon chlorine bond lengths restrained using DFIX and some of the solvent molecules were restrained with *DELU*, *SIMU* and *ISOR*.

$[(\mathbf{L^1})_4Zn_5(O_2NC_6H_4PO_4)_4]^{2+}$. Only a quarter of the molecule (comprising one ligand, one NPP dianion and one and one quarter Zn^{2+} atoms) was contained in the asymmetric cell and the remaining 3/4 of the assembly formed by symmetry operations. The structure contained whole molecule disorder with the ligand and NPP dianion modelled over two positions using the *PART* instruction. Due to the extensive disorder some bonds in the disordered ligand had to be constrained with *DFIX* instructions as well as *AFIX* 66 for the 6-membered rings. Further to this *ISOR* was used globally for the carbon,

nitrogen and oxygen atoms. The structure contained diffuse electron density, which despite attempts, could not be modelled and was removed using the solvent mask facility in Olex2.[4] The solvent mask removed a total of 284 electrons per pentanuclear assembly which corresponds to 2 perchlorate anions 2 molecules of acetonitrile and 3 molecules of ethyl acetate. Despite removal of extensive number of solvent molecules with the use of the solvent mask function satisfactory data was obtained.

$[(\mathbf{L^2})_2Zn_2(H_2PO_4)_2](ClO_4)_3$. Only a half of the helicate molecule was present in the asymmetric unit and the remaining half was formed by symmetry operations. The structure contained a rotationally disorder perchlorate molecule and the oxygen atoms were modelled in two positions using the *PART* instructions and constrained using *DELU*, *SIMU* and *ISOR*. These three restraints were also used on one of the dichloromethane solvent molecules.

$[(\mathbf{L^2})_4Zn_5(O_2NC_6H_4PO_4)_4](ClO_4)_2$. Unlike the **L**[1] derivative the whole molecule was present in the asymmetric unit cell. However, two of the ligands had disorder associated with the ethyl spacer unit and one of the thiazole rings and these were modelled in two positions using the *PART* instructions and constrained using *DELU*, *SIMU* and *ISOR*. Also present was solvent disorder that despite attempts could not be successfully modelled and as a result the diffuse electron density was removed using the solvent mask facility in Olex2, resulting in voids in the crystal structure.[4] The solvent mask removed a total of 293 electrons in the asymmetric unit which corresponds to one perchlorate anion and eleven molecules of acetonitrile.

Compound	$[(\mathbf{L^1})_2Zn_2(ClO_4)_2](ClO_4)_2{\cdot}4CH_2Cl_2$	$[(\mathbf{L^1})_2Zn_2(H_2PO_4)_2](ClO_4)_3 \cdot 4.3\ CH_2Cl_2 \cdot 0.65\ CH_3OH \cdot H_2O$	$[(\mathbf{L^1})_4Zn_5(O_2NC_6H_4PO_4)_4]^{2+}$	$[(\mathbf{L^2})_2Zn_2(H_2PO_4)_2](ClO_4)_3{\cdot}2CH_2Cl_2$	$[(\mathbf{L^2})_4Zn_5(O_2NC_6H_4PO_4)_4](ClO_4)_2{\cdot}3H_2O{\cdot}1.5MeCN$
Formula	$C_{44}H_{44}Cl_{12}N_{12}O_{18}S_4Zn_2$	$C_{44.961}H_{42}Cl_{11.615}N_{12}O_{17.654}\ PS_4Zn_2$	$C_{104}H_{96}N_{28}O_{24}P_4S_8Zn_5$	$C_{20}H_{21}Cl_{5.5}N_6O_8P_{0.5}S_2Zn$	$C_{99.01}H_{81}ClN_{29.505}O_{31}P_4S_8\ Zn_5$
M	1713.40	1734.71	2829.50	1626.858	2922.98
Crystal system	Triclinic	Triclinic	Tetragonal	Monoclinic	Triclinic
Space group	P-1	P-1	P42/n	C2/c	P-1
a (Å)	10.7084(4)	13.360(6)	20.5102(2)	21.3603(11)	17.873(6)
b (Å)	12.0876(4)	15.901(6)	20.5102(2)	17.9531(10)	18.066(5)
c (Å)	13.9932(5)	19.103(9)	16.9068(3)	17.0152(8)	23.578(7)
α (˚)	69.9240(10)	97.16(2)	90	90	109.971(7)
β (˚)	86.5010(10)	97.852(19)	90	104.627(2)	92.993(14)
γ (˚)	80.1050(10)	99.846(15)	90	90	90.039(12)
V (Å^3)	1675.90(10)	3915(3)	7112.15(16)	6313.6(6)	7144(4)

Z	1	2	2	4	2
ρ_{calc} (Mg cm^{-1})	1.6976	1.4714	1.3211	1.712	1.3587
F(000)	867.2262	1753.4510	2892.0006	3293.377	2976.6115
Crystal dimensions (mm)	0.25, 0.23, 0.18	0.18, 0.05, 0.05	0.3, 0.26, 0.25	0.19, 0.11, 0.09	0.15, 0.12, 0.08
Reflections measured	57931	100727	38209	35197	198280
Range (˚)	33.36 ≤ θ ≥ 2.47	28.36 ≤ θ ≥ 2.11	74.61 ≤ θ ≥ 3.05	30.53 ≤ θ ≥ 1.97	28.23 ≤ θ ≥ 1.64
hkl range indices	16 ≤ h ≥ -16, 18 ≤ k ≥ -17, 21 ≤ l ≥ 0	17 ≤ h ≥ -17, 20 ≤ k ≥ -21, 25 ≤ l ≥ 0	25 ≤ h ≥ 0, 25 ≤ k ≥ 0, 21 ≤ l ≥ 0	29 ≤ h ≥ -30, 25 ≤ k ≥ 0, 24 ≤ l ≥ 0	23 ≤ h ≥ -23, 22 ≤ k ≥ -24, 31 ≤ l ≥ 0
Nº independent reflections	12868	19253	7173	9580	35094
Reflections with *I* > 2σ(*I*)	10926	12817	5910	7378	23731
R_{int}	0.0308	0.0852	0.0323	0.0693	0.0799
Final R_1 values	0.0408	0.0880	0.0681	0.0637	0.0666
Final $wR(F^2)$ values	0.1113	0.2551	0.1710	0.1709	0.1810
Final R_1 values (all data)	0.0502	0.1312	0.0809	0.0835	0.1002
Final $wR(F^2)$ values (all data)	0.1200	0.2250	0.1895	0.1903	0.2116
GOF	1.0356	1.0624	1.0813	1.0498	1.0702
Refined parameters	462	899	701	400	1771
Restraints	40	79	92	42	182
Largest peak and hole (e Å^{-3})	0.9528, -1.2422	2.0026, -1.3206	1.4891, -0.5472	2.6931, -1.5080	1.6108, -1.4856
CCDC Number	2287553	2287554	2287563	2287565	2287566

Mass Spectrometry Studies.

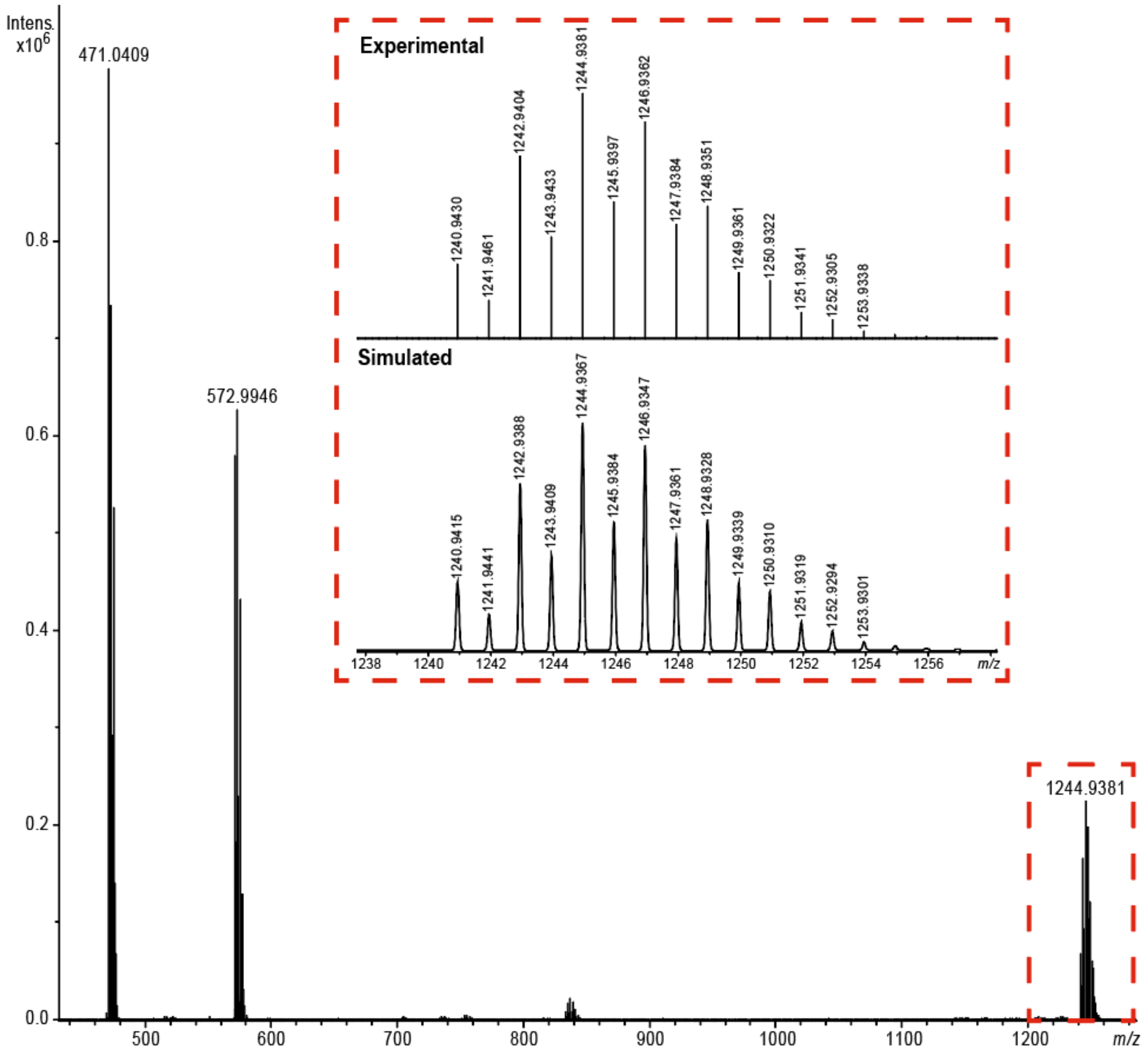

Figure S1.7. Electrospray ionisation mass spectrum of [(**L**[1])$_2$Zn$_2$](ClO$_4$)$_4$. Expanded region (red dashed) showing the experimental (top) and the simulated (bottom) isotope pattern relating to the species at 1244.9381 m/z - [$C_{40}H_{40}N_{12}S_4Zn_2Cl_3O_{12}$]$^+$ e.g. {[(**L**[1])$_2$Zn$_2$](ClO$_4$)$_3$}$^+$.

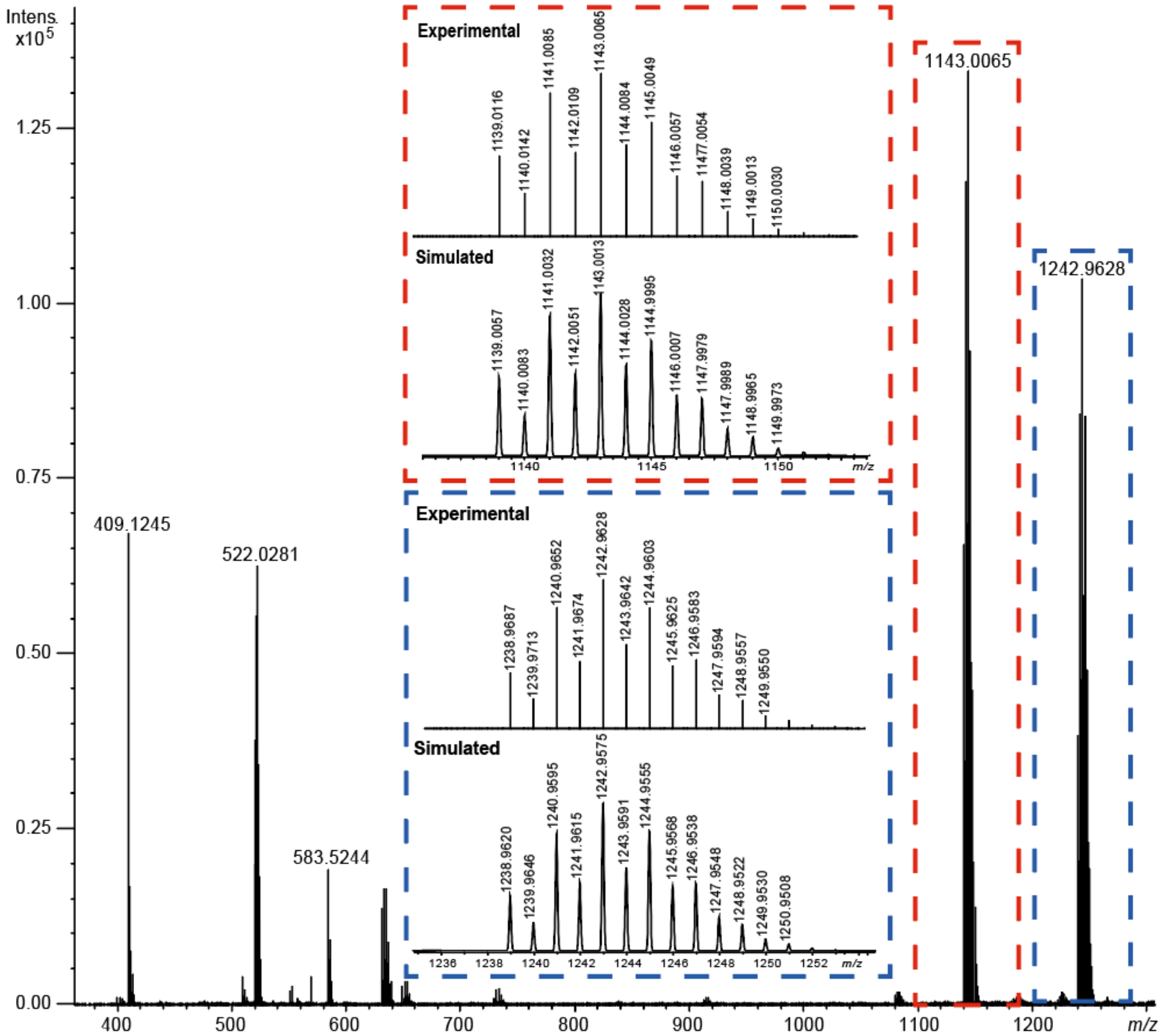

Figure S1.8. Electrospray ionisation mass spectrum of [(**L**[1])$_2$Zn$_2$(H$_2$PO$_4$)](ClO$_4$)$_3$. Expanded region (red dashed) showing the experimental (top) and the simulated (bottom) isotope pattern relating to the species at 1143.0075 m/z - [$C_{40}H_{41}N_{12}S_4Zn_2ClO_8P$]$^+$ e.g. {[(**L**[1])$_2$Zn$_2$(HPO$_4$)](ClO$_4$)}$^+$. Expanded region (blue dashed) showing the experimental (top) and the simulated (bottom) isotope pattern relating to the species at 1242.9638 m/z - [$C_{40}H_{42}N_{12}S_4Zn_2Cl_2O_{12}P$]$^+$ e.g. {[(**L**[1])$_2$Zn$_2$(H$_2$PO$_4$)](ClO$_4$)}$^+$.

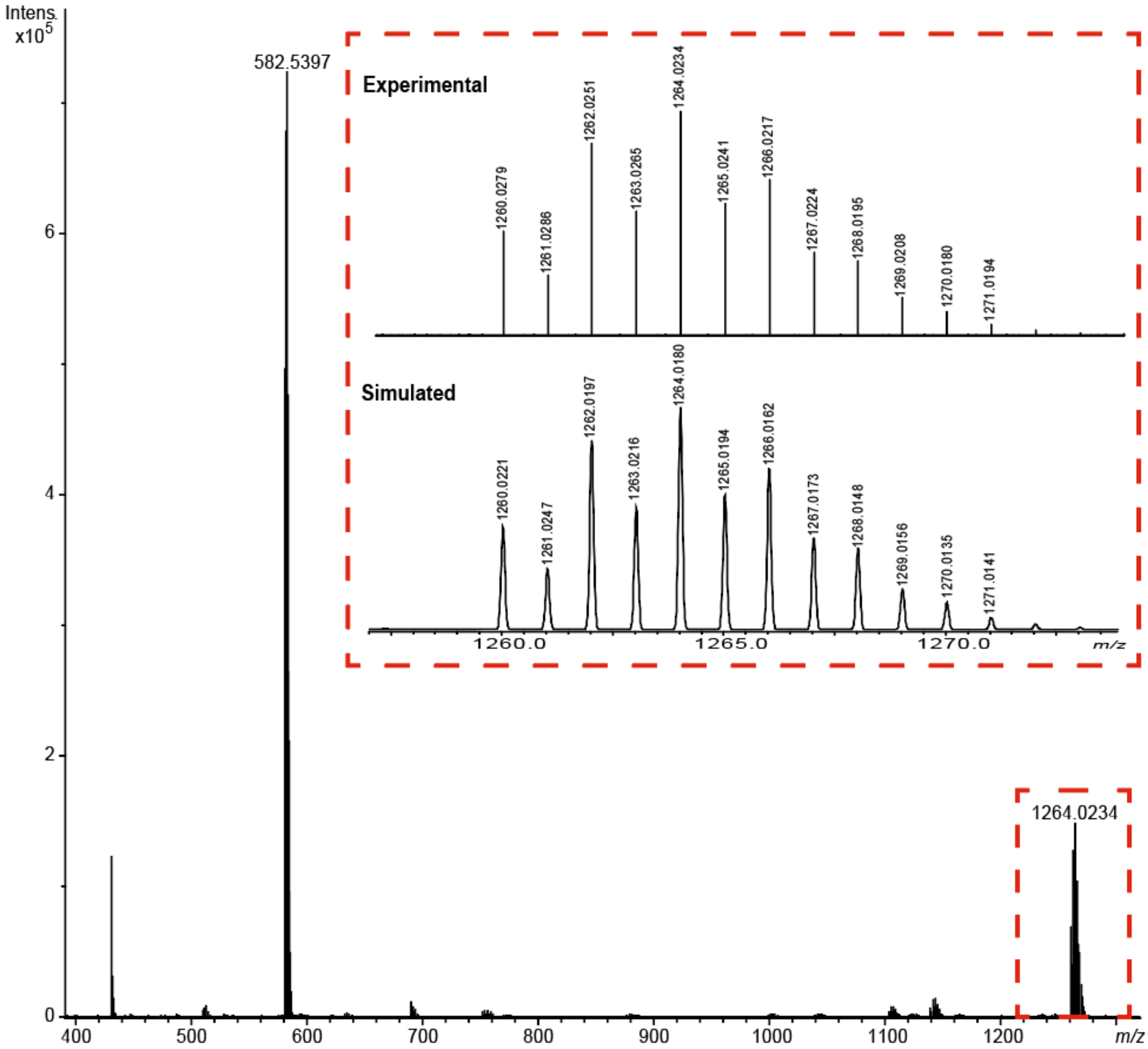

Figure S1.9. Electrospray ionisation mass spectrum of [(**L**[1])$_4$Zn$_5$(NPP)$_4$](ClO$_4$)$_2$. Expanded region (red dashed) showing the experimental (top) and the simulated (bottom) isotope pattern relating to the species at 1264.0234 m/z - [$C_{46}H_{44}N_{13}S_4Zn_2ClO_{10}P$]$^+$ e.g. {[(**L**[1])$_2$Zn$_2$(NPP)](ClO$_4$)}$^+$.

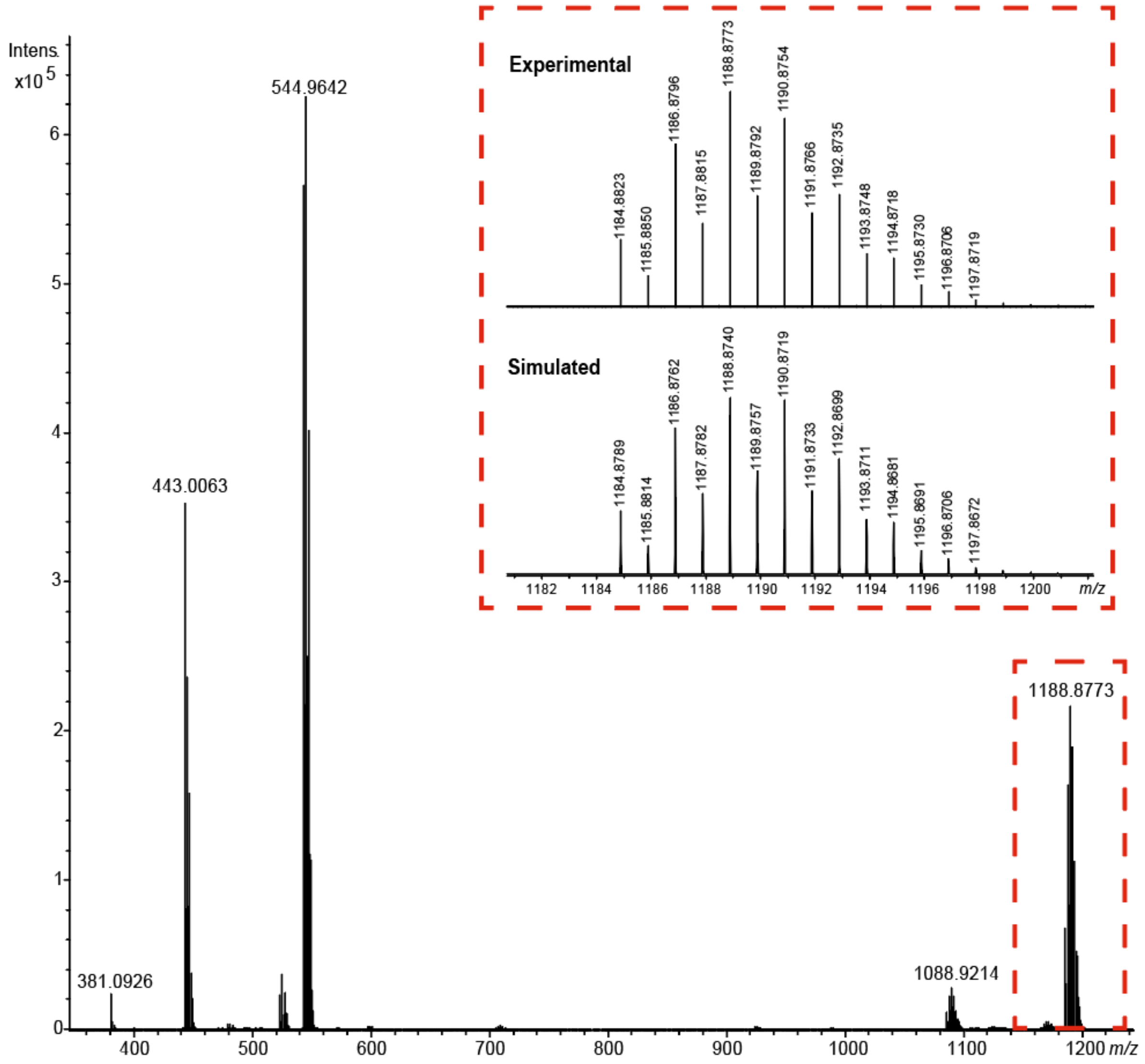

Figure S1.10. Electrospray ionisation mass spectrum of $[(\mathbf{L^2})_2Zn_2](ClO_4)_4$. Expanded region (red dashed) showing the experimental (top) and the simulated (bottom) isotope pattern relating to the species at 1188.8773 m/z - $[C_{36}H_{32}N_{12}S_4Zn_2Cl_3O_{12}]^+$ e.g. $\{[(\mathbf{L^2})_2Zn_2](ClO_4)_3\}^+$.

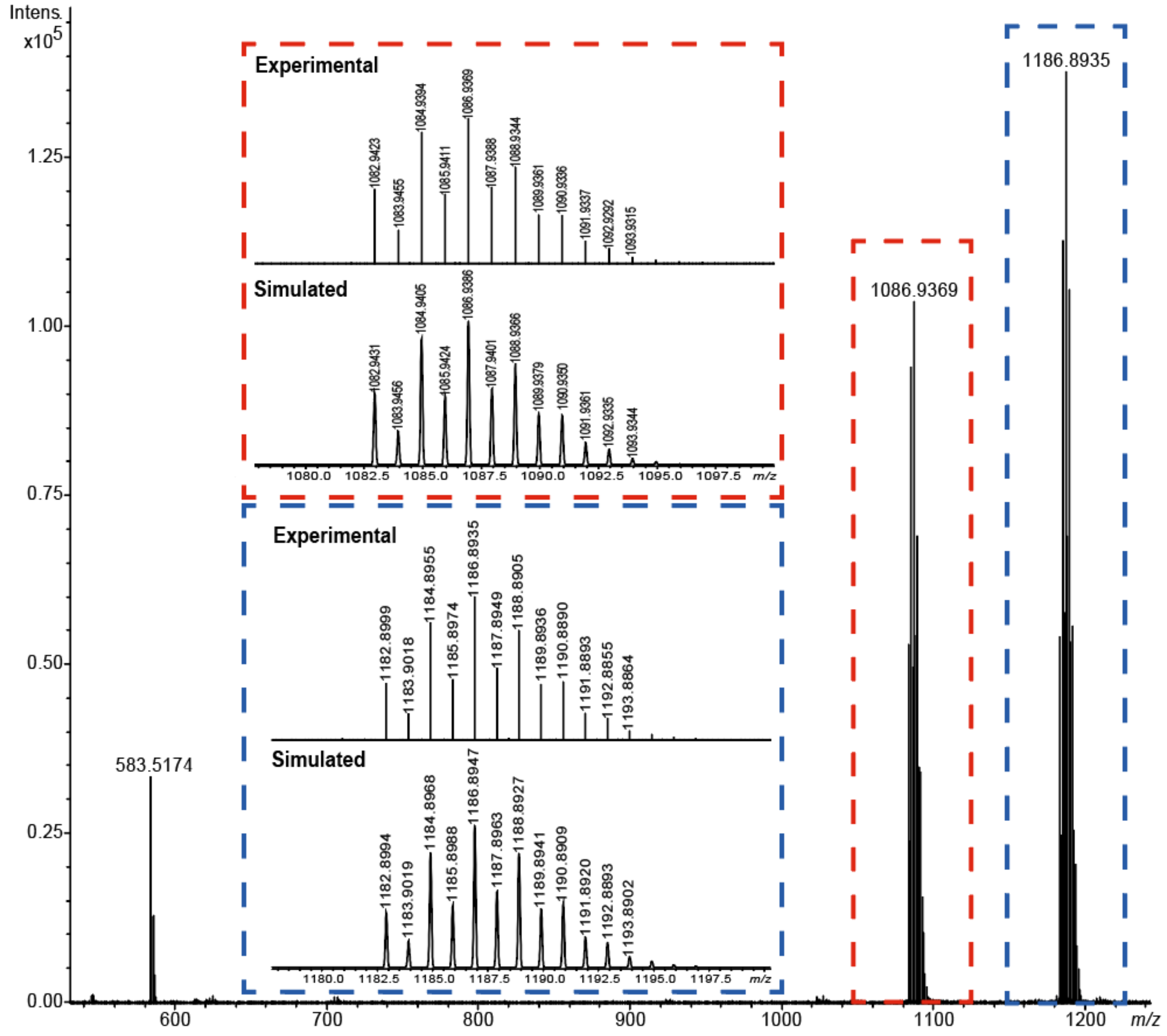

Figure S1.11. Electrospray ionisation mass spectrum of [(**L**[2])$_2$Zn$_2$(H$_2$PO$_4$)](ClO$_4$)$_3$. Expanded region (red dashed) showing the experimental (top) and the simulated (bottom) isotope pattern relating to the species at 1086.9378 m/z - [C$_{36}$H$_{33}$N$_{12}$S$_4$Zn$_2$ClO$_8$P]$^+$ e.g. {[(**L**[2])$_2$Zn$_2$(HPO$_4$)(ClO$_4$)]}$^+$. Expanded region (blue dashed) showing the experimental (top) and the simulated (bottom) isotope pattern relating to the species at 1186.8943 m/z - [C$_{36}$H$_{34}$N$_{12}$S$_4$Zn$_2$Cl$_2$O$_{12}$P]$^+$ e.g. {[(**L**[2])$_2$Zn$_2$(H$_2$PO$_4$)(ClO$_4$)$_2$]}$^+$.

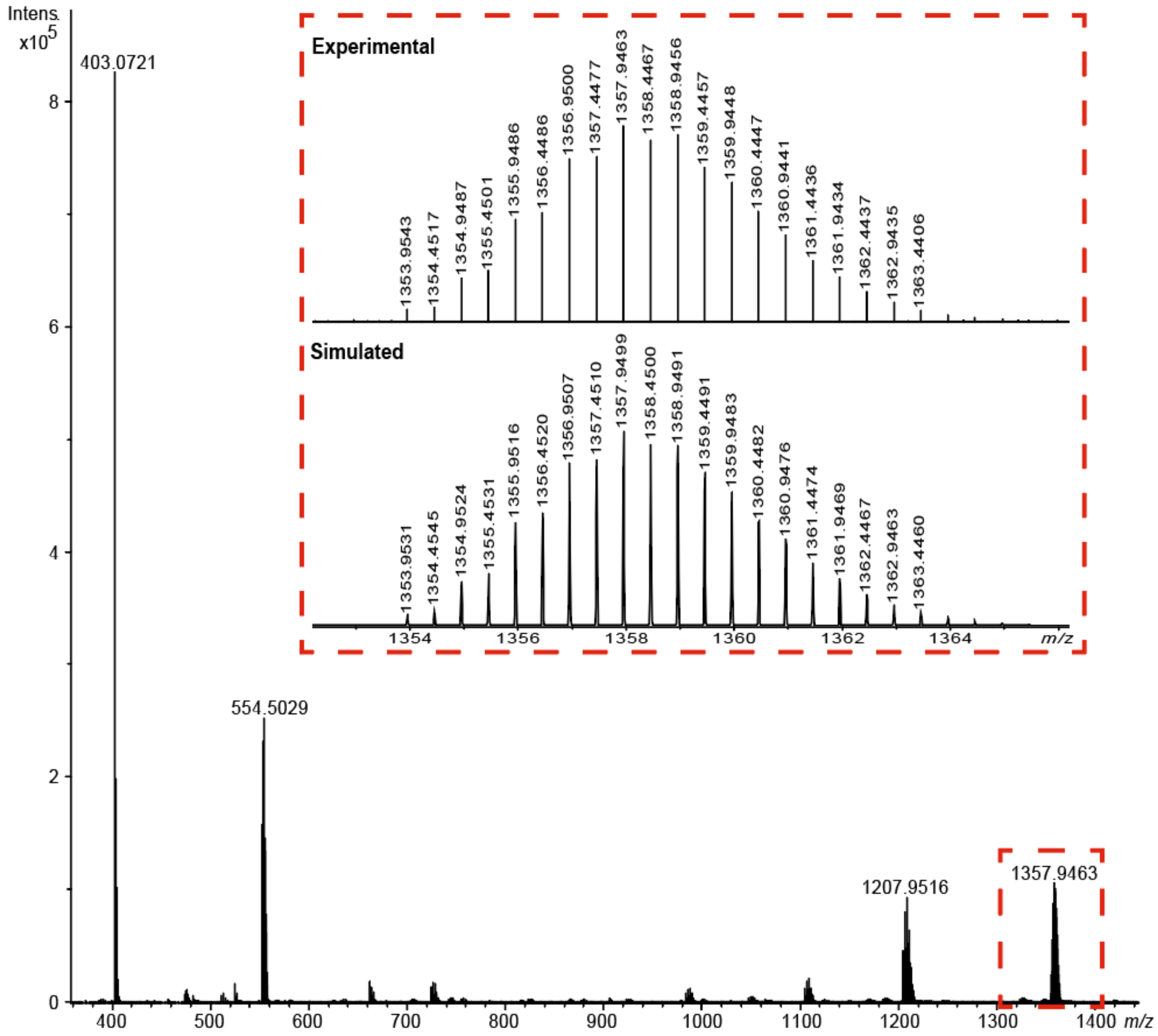

Figure S1.12. Electrospray ionisation mass spectrum of $[(\mathbf{L^2})_4Zn_5(NPP)_4](ClO_4)_2$. Expanded region (red dashed) showing the experimental (top) and the simulated (bottom) isotope pattern relating to the species at 1357.9463 m/z - $[C_{96}H_{80}N_{28}S_8Zn_5O_{24}P_4]^{2+}$ e.g. $\{[(\mathbf{L^2})_4Zn_5(NPP)_4]\}^{2+}$.

NMR analysis of the Zn^{2+} complexes of L^1 and L^2.

Analysis of the complexes by ^1H NMR was carried out in CD_3CN/CD_3OD (2:1) as this solvent system dissolved all the complexes although precipitates did form in the Na_2NPP system. The ^1H NMR of $[(\mathbf{L^1})_2Zn_2(H_2O)_2(ClO_4)_2]^{2+}$ gave a set of broad complex peaks in the aromatic region and from the ^1H and HSQC this would appear to be three separate species with EXSY/NOESY NMR showing clear exchange between these signals. Addition of 1 equivalent of $H_2PO_4^-$ significantly changes the ^1H NMR with 5 aromatic signals present attributable to one species e.g. $[(\mathbf{L^1})_2Zn_2(H_2PO_4)]^{3+.}$ Addition of further dihydrogen phosphate only resulted in a slight change in chemical shift (attributable to the change in ionic strength and decrease in *p*H) indicating that the complex only binds one phosphate anion. The ^1H NMR of $[(\mathbf{L^2})_2Zn_2]^{4+}$ gave signals attributable to two species which were again interconverting. Addition of 1 equivalent of $H_2PO_4^-$ changes the ^1H NMR significantly resulting in a

complex spectrum with signals corresponding to at least three species and again exchange between these species was observed. However, addition of a further equivalent of $H_2PO_4^-$ a change in the ^1H NMR is observed and now a single complex is the major species. We attribute this behaviour to the ability of $[(\mathbf{L^2})_2Zn_2]^{4+}$ to bind two phosphate anions and reaction of $[(\mathbf{L^2})_2Zn_2]^{4+}$ with two equivalents of $H_2PO_4^-$ gives $[(\mathbf{L^2})_2Zn_2(H_2PO_4)_2]^{2+}$. Indeed, further examination of the ^1H NMR where one equivalent of dihydrogen phosphate is added shows that signals corresponding to $[(\mathbf{L^2})_2Zn_2]^{4+}$ and $[(\mathbf{L^2})_2Zn_2(H_2PO_4)_2]^{2+}$ are present with the remaining signals attributable to $[(\mathbf{L^2})_2Zn_2(H_2PO_4)]^{3+}$. This is supported by the solid-state studies that shows the two Zn^{2+} are coordinated by both bridging $H_2PO_4^-$ and ClO_4^- anions and it is perfectly reasonable that the perchlorate anion could be displaced by a dihydrogen phosphate anion giving $[(\mathbf{L^2})_2Zn_2(H_2PO_4)_2]^{2+}$. The complex ^1H NMR behaviour is a consequence of the 1,4-diaminobutane and a 1,2-diaminoethane spacer groups which are sufficiently flexible enough to allow a number of potential conformers to be present in solution. Some of the signals may be attributable to the racemic helicate and achiral mesocate species but other species may involve coordination to different solvent molecules (e.g. $[(\mathbf{L^1})_2Zn_2(H_2O)_2(ClO_4)_2]^{2+}$) and slow exchange in solution. Regardless, it is clear that both ligands coordinate Zn^{2+} giving dinuclear species and these complexes can bind dihydrogen phosphate anions.

The ^1H NMR of $[(\mathbf{L^2})_4Zn_5(NPP)_4]^{2+}$ shows one major species with signals corresponding to the ligand strand and *p*-nitrophenyl phosphate but the solubility of $[(\mathbf{L^1})_4Zn_5(NPP)_4]^{2+}$ limits analysis at the correct stochiometric proportions to form the pentanuclear species.

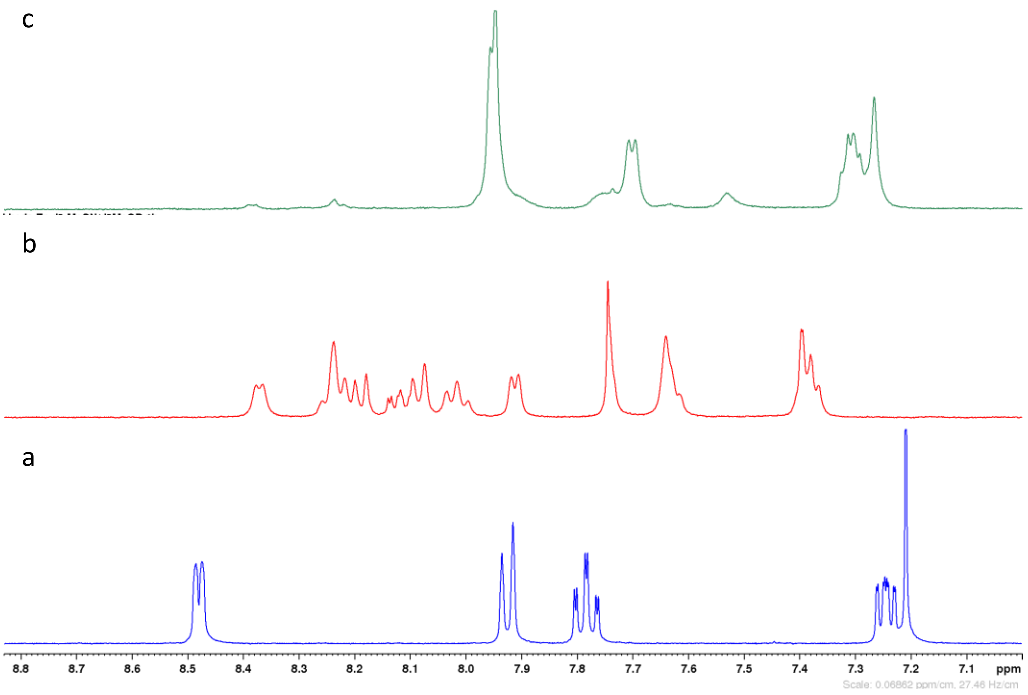

Figure S1.13. Aromatic regions in the ^{1}H NMR spectra (CD_3OD/CD_3CN) of (a) **L^1** and (b) **L^1** plus $Zn(OTf)_2$ and (c) **L^1** plus $Zn(OTf)_2$ and 0.5 equivalents of $Bu_4NH_2PO_4$.

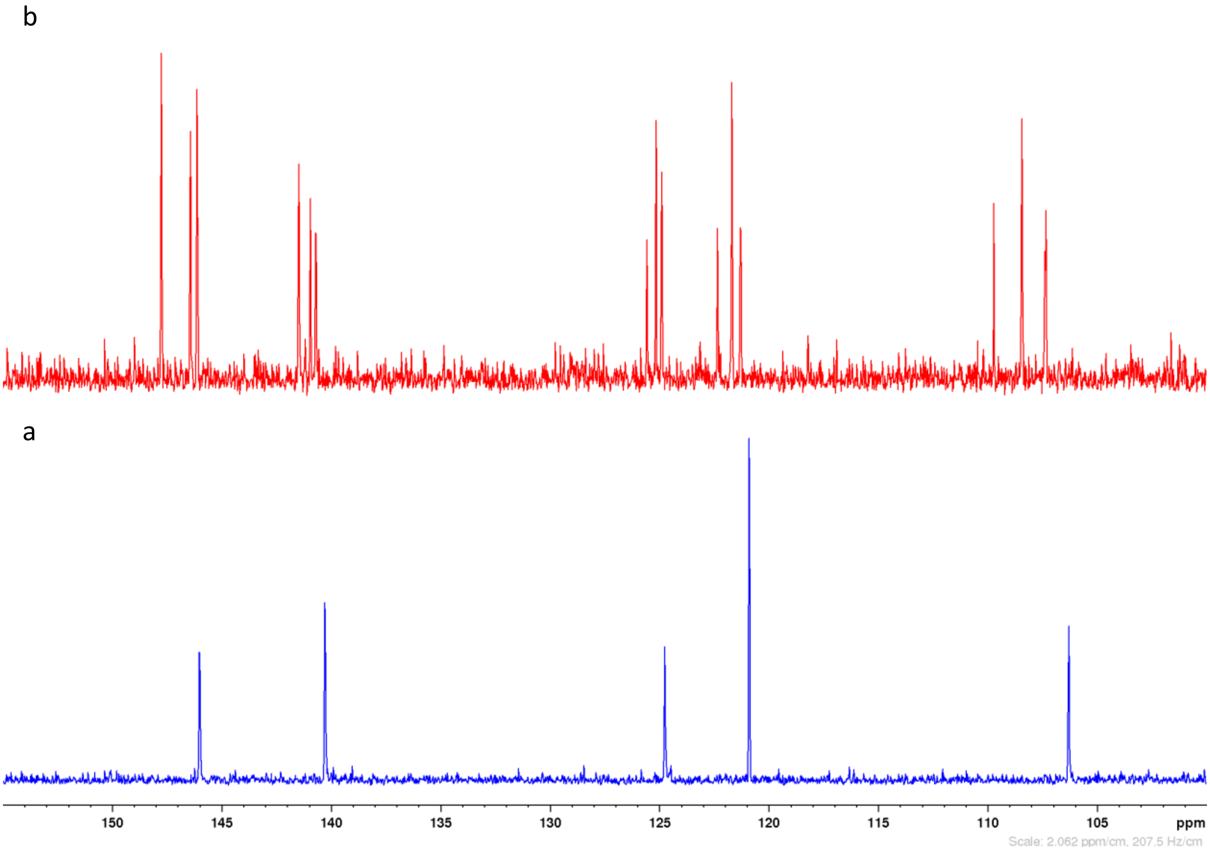

Figure S1.14. Aromatic regions in the ^{13}C NMR spectra (CD_3OD/CD_3CN 1:2) of (a) **L^1** plus $Zn(OTf)_2$ and 0.5 equivalents of $Bu_4NH_2PO_4$and (b) **L^1** plus $Zn(OTf)_2$. The number of carbon signals indicate one species is present for $[(\mathbf{L^1})_2Zn_2(H_2PO_4)]^{3+}$ whereas at least three species are present for $[(\mathbf{L^1})_2Zn_2]^{4+}$.

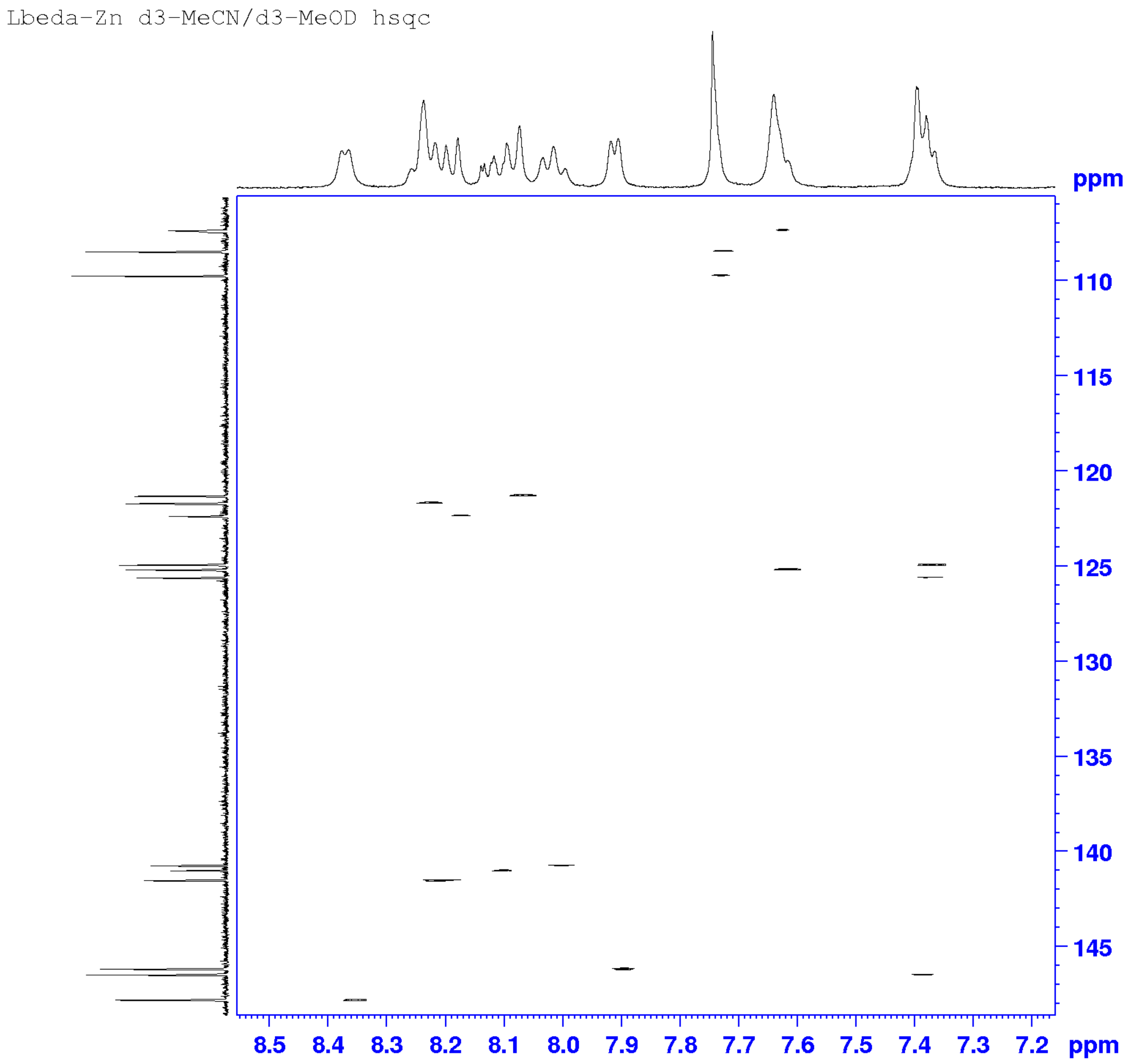

Figure S1.15. Aromatic regions in the HSQC spectra (CD_3OD/CD_3CN 1:2) of **L**[1] plus $Zn(OTf)_2$ showing the number of ^{13}C signals indicating the formation of three major species.

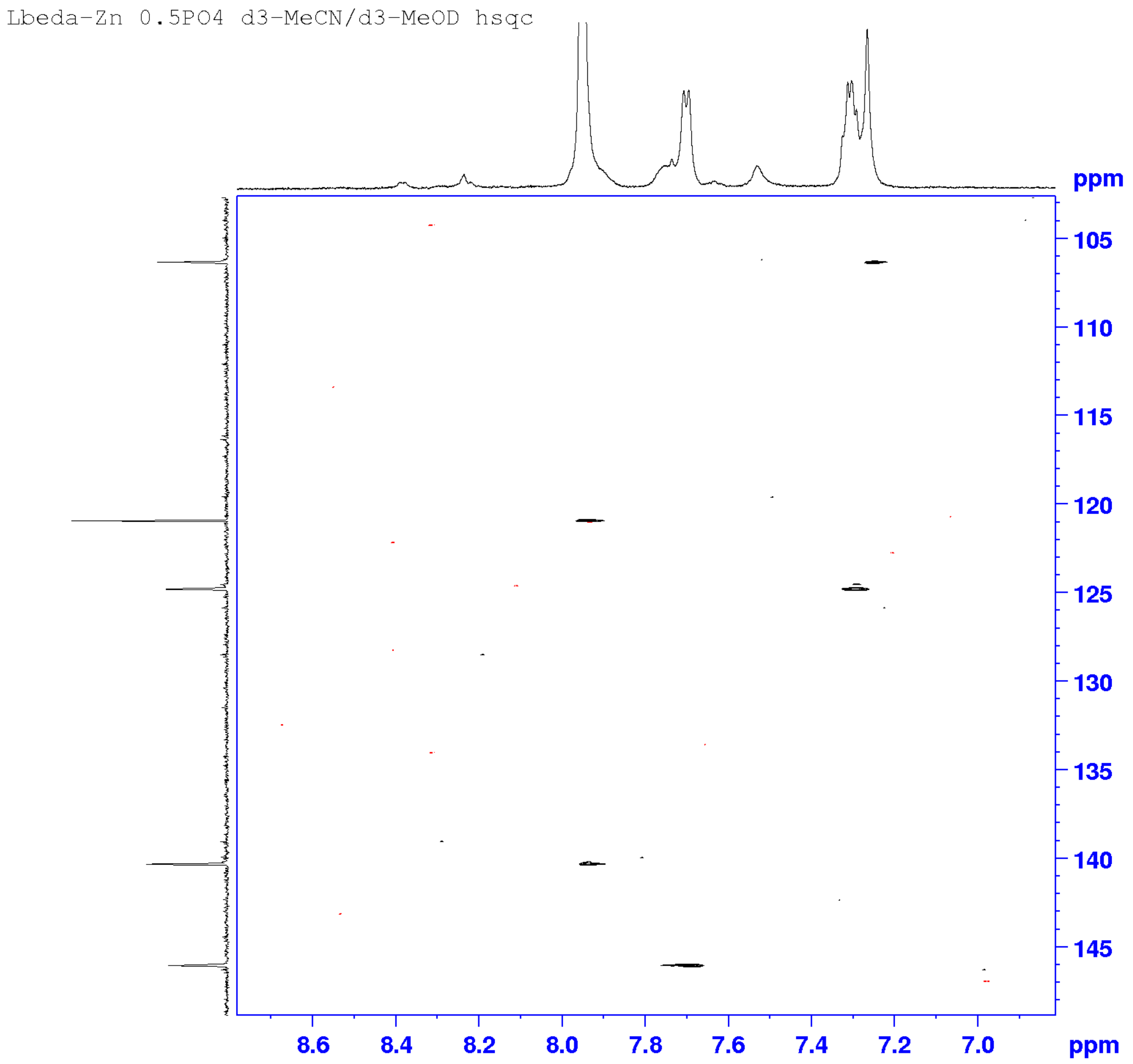

Figure S1.16. Aromatic regions in the HSQC spectra (CD_3OD/CD_3CN 1:2) of **L[1]** plus $Zn(OTf)_2$ and 0.5 equivalents of $Bu_4NH_2PO_4$ showing the reduced number of ^{13}C signals indicating the formation of one species.

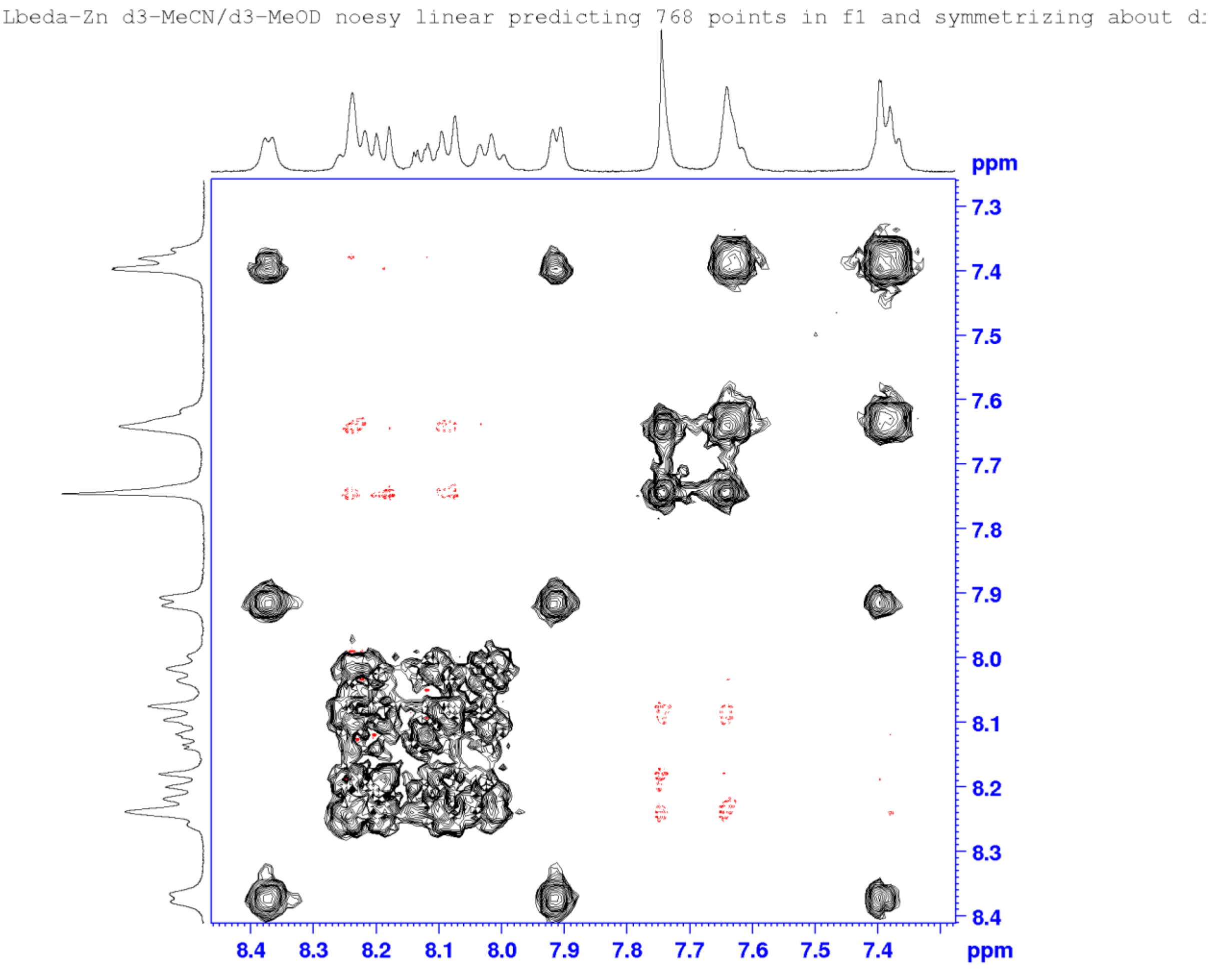

Figure S1.17. Aromatic regions in the ^{1}H–^{1}H NOESY spectrum of **L[1]** plus $Zn(OTf)_2$ (CD_3OD/CD_3CN 1:2) with the in-phase cross peaks indicative of exchange.

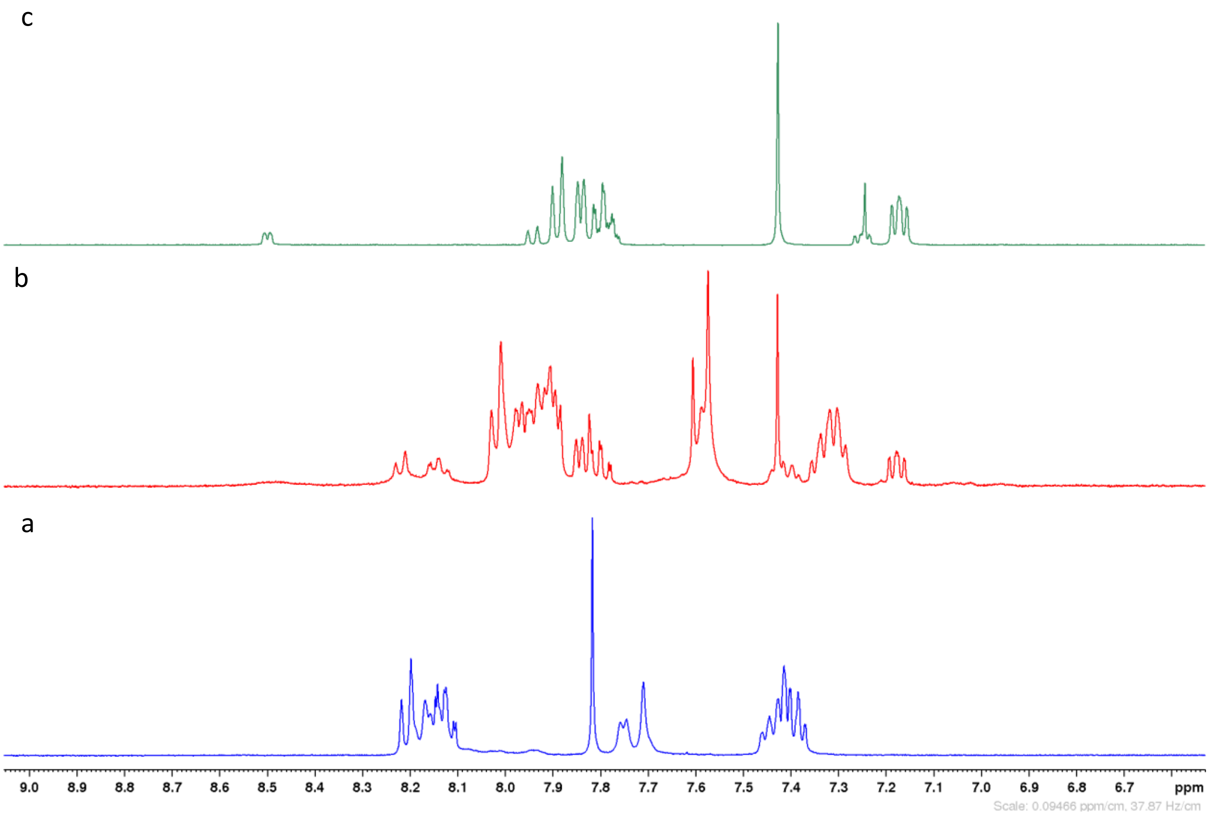

Figure S1.18. Aromatic regions in the ^{1}H NMR spectra (CD_3OD/CD_3CN 1:2) of (a) $\mathbf{L^2}$ plus $Zn(OTf)_2$ (b) $\mathbf{L^2}$ plus $Zn(OTf)_2$ and 0.5 equivalents of $Bu_4NH_2PO_4$ (c) $\mathbf{L^2}$ plus $Zn(OTf)_2$ plus a further 0.5 equivalents of $Bu_4NH_2PO_4$.

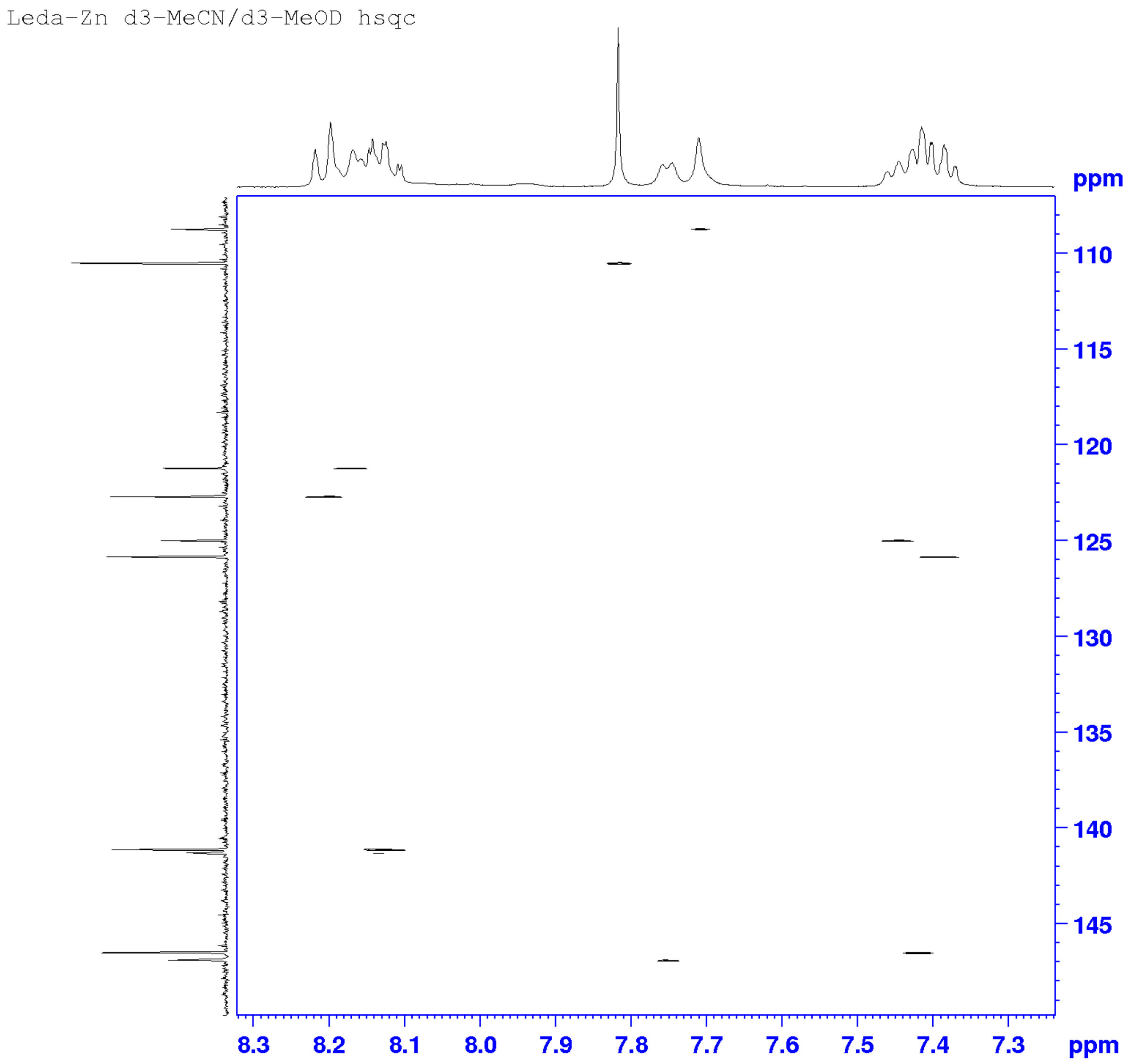

Figure S1.19. Aromatic regions in the HSQC spectra (CD_3OD/CD_3CN 1:2) of **L^2** plus $Zn(OTf)_2$ showing a number of ^{13}C signals indicating the formation of two major species.

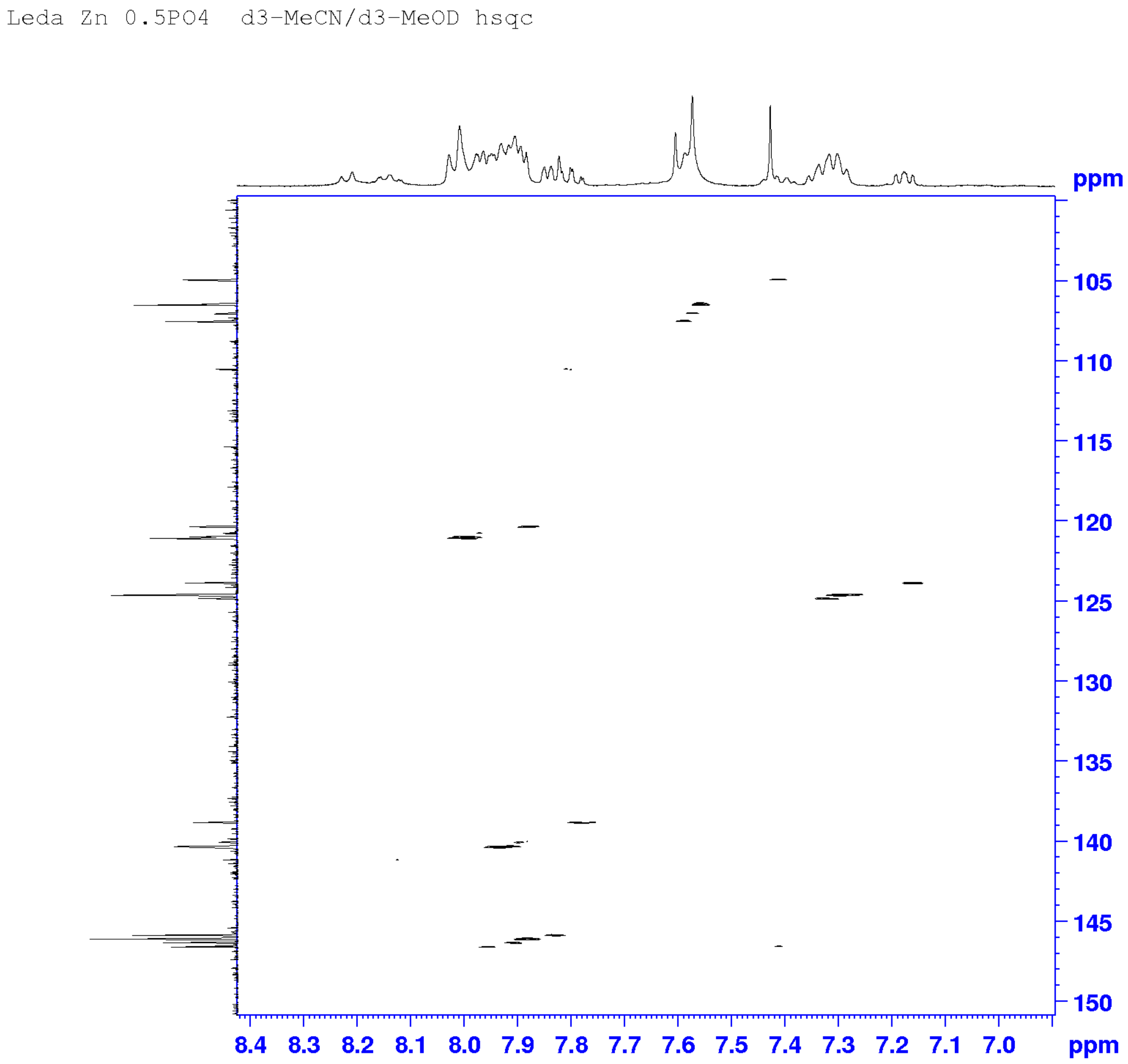

Figure S1.20. Aromatic regions in the HSQC spectra (CD_3OD/CD_3CN 1:2) of **L^2** plus $Zn(OTf)_2$ and 0.5 equivalents of $Bu_4NH_2PO_4$ showing a number of ^{13}C signals indicating the formation of a number of different species.

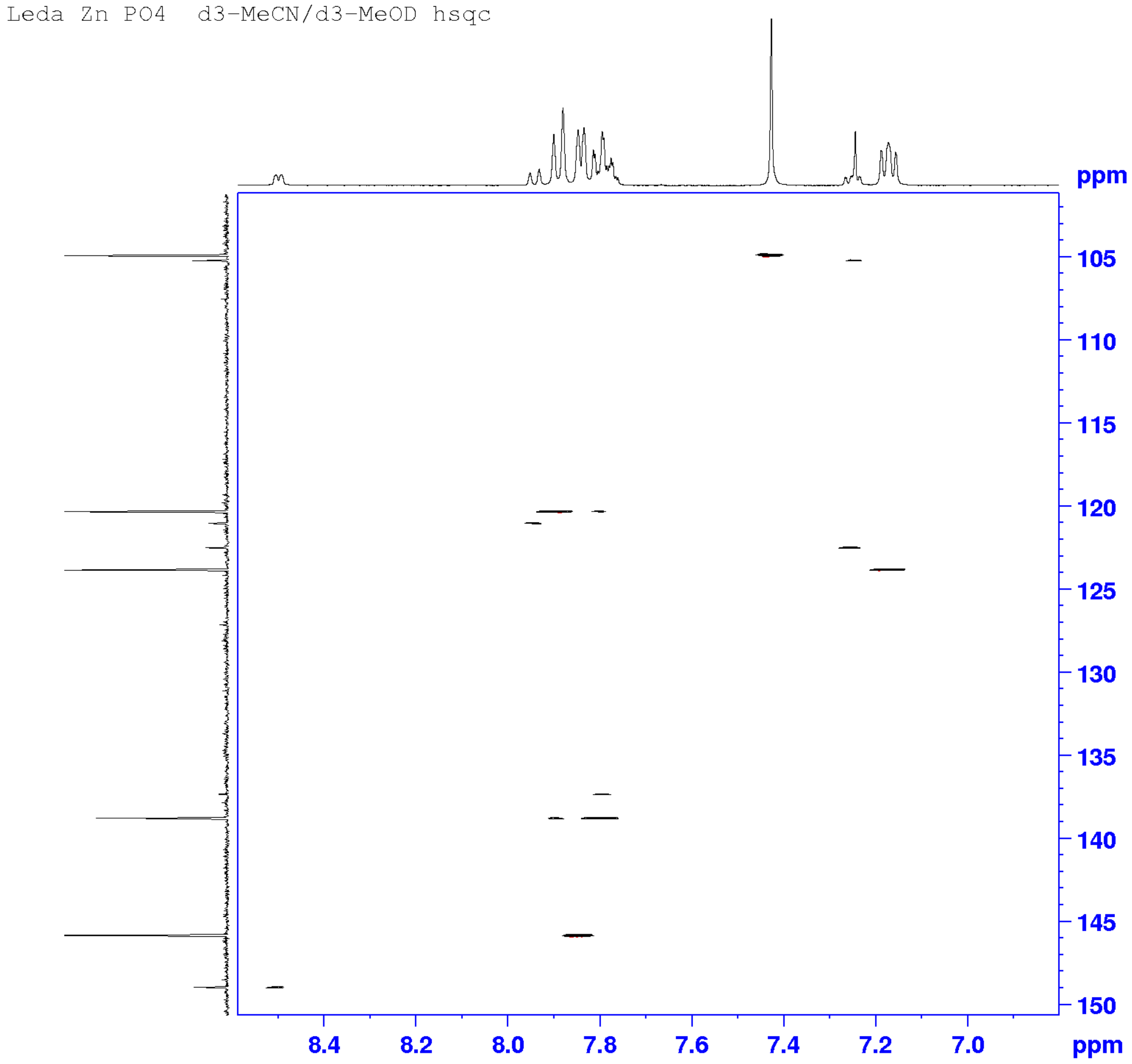

Figure S1.21. Aromatic regions in the HSQC spectra (CD_3OD/CD_3CN 1:2) of $\mathbf{L^1}$ plus $Zn(OTf)_2$ and 1 equivalent of $Bu_4NH_2PO_4$ showing the reduced number of ^{13}C signals indicating the formation of one major species.

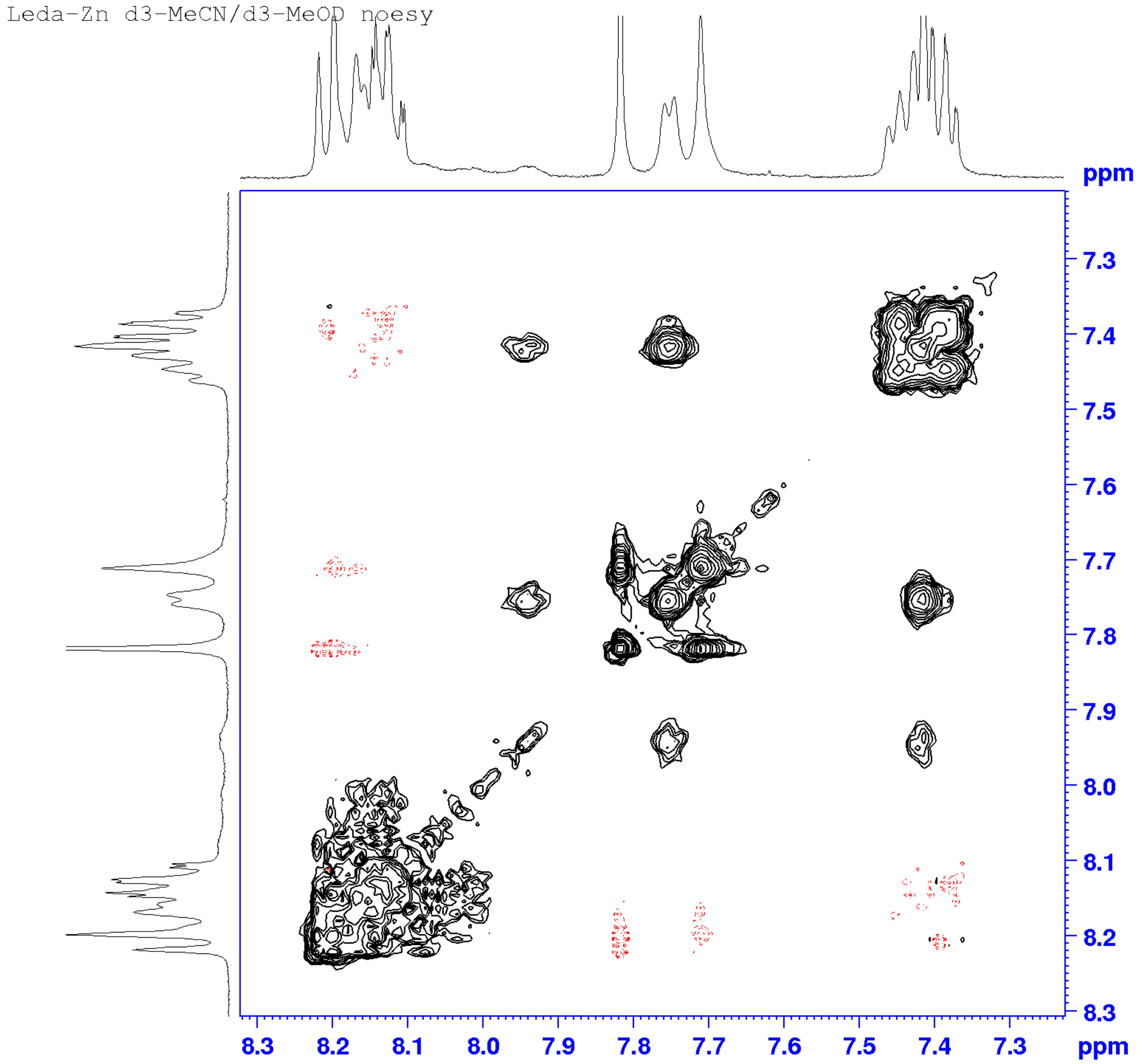

Figure S1.22. Aromatic regions in the ^1H–^1H NOESY spectrum of **L²** plus $Zn(OTf)_2$ (CD_3OD/CD_3CN 1:2) with the in phase cross peaks indicative of exchange.

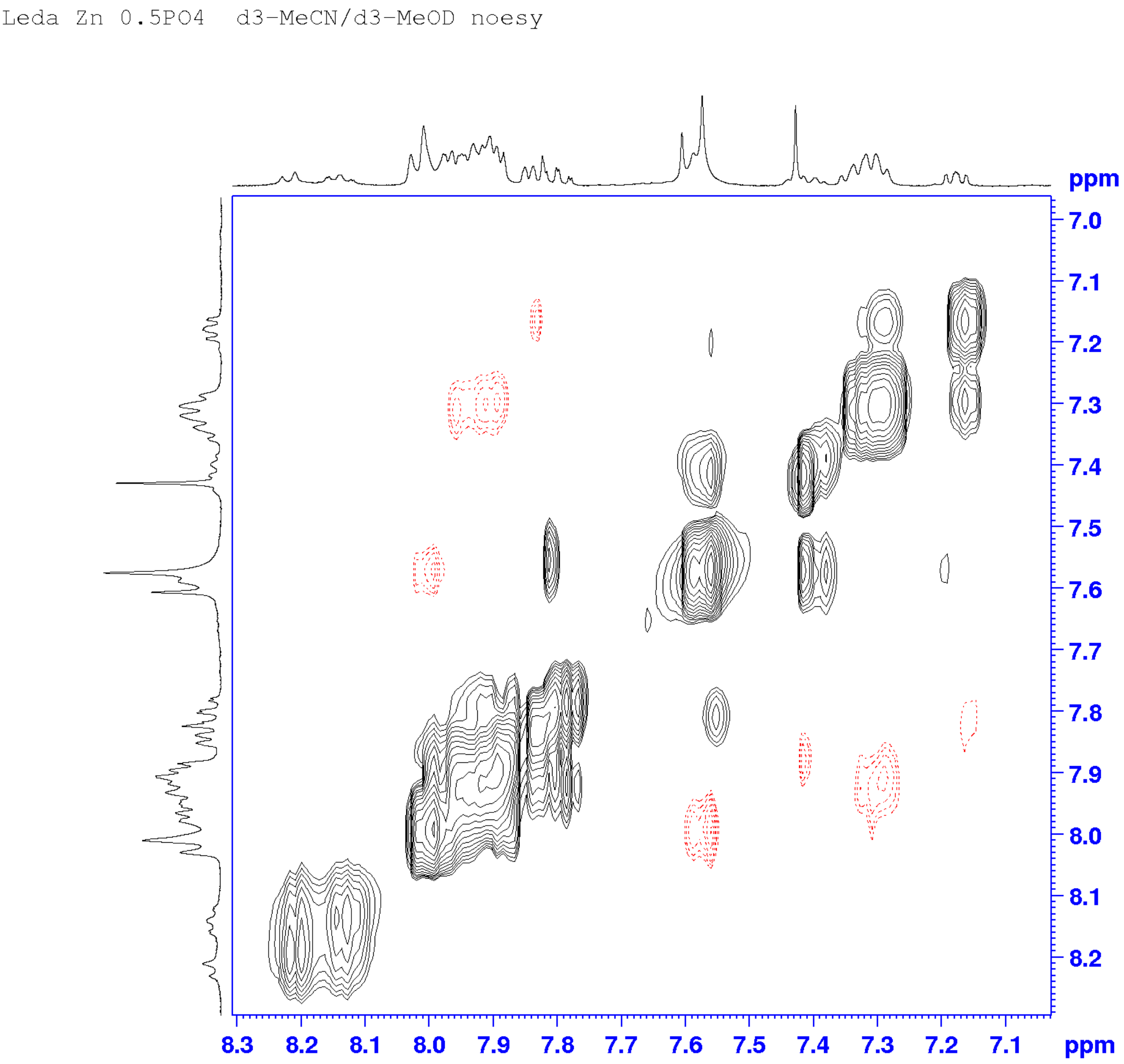

Figure S1.23. Aromatic regions in the ^1H–^1H NOESY spectrum of **L[1]** plus $Zn(OTf)_2$ and 0.5 equivalents of $Bu_4NH_2PO_4$ (CD_3OD/CD_3CN 1:2) with the in-phase cross peaks indicative of exchange.

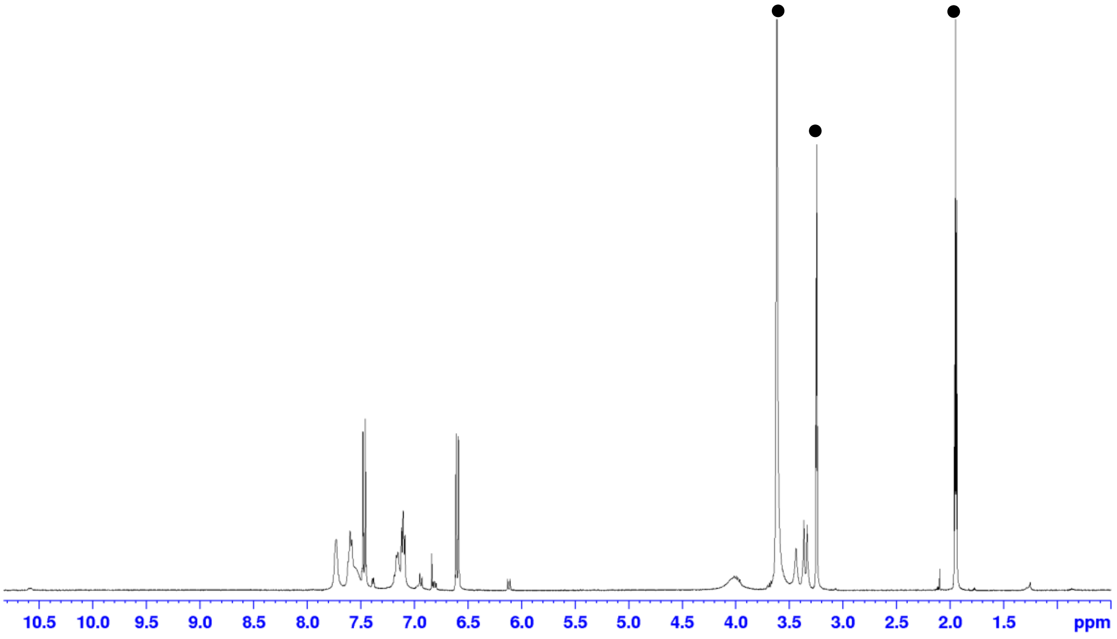

Figure S1.24. ^{1}H NMR spectra (CD_3OD/CD_3CN 1:2) of $\mathbf{L^2}$ plus $Zn(OTf)_2$ and 1 equivalent of Na_2NPP showing one main species. Peaks marked ● are those arising from solvent (CHD_2OD / CHD_2CN / HOD).

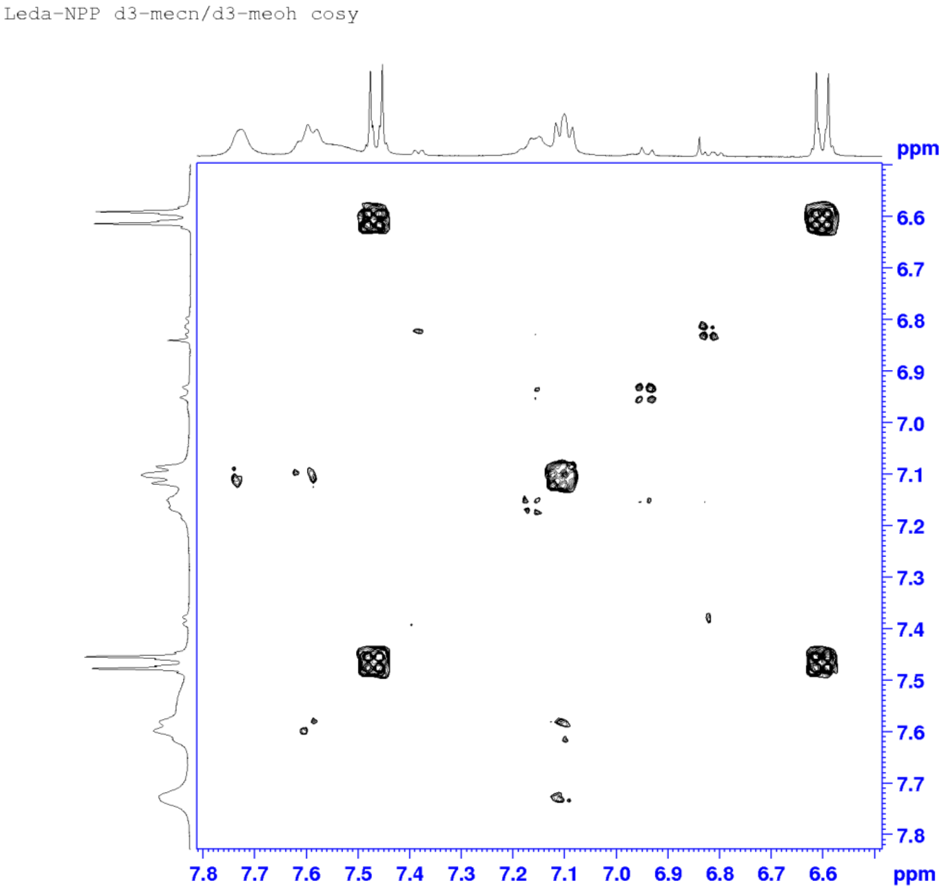

Figure S1.25. Aromatic regions in the ^{1}H–^{1}H COSY NMR spectra (CD_3OD/CD_3CN 1:2) of $\mathbf{L^2}$ plus $Zn(OTf)_2$ and 1 equivalent of Na_2NPP.

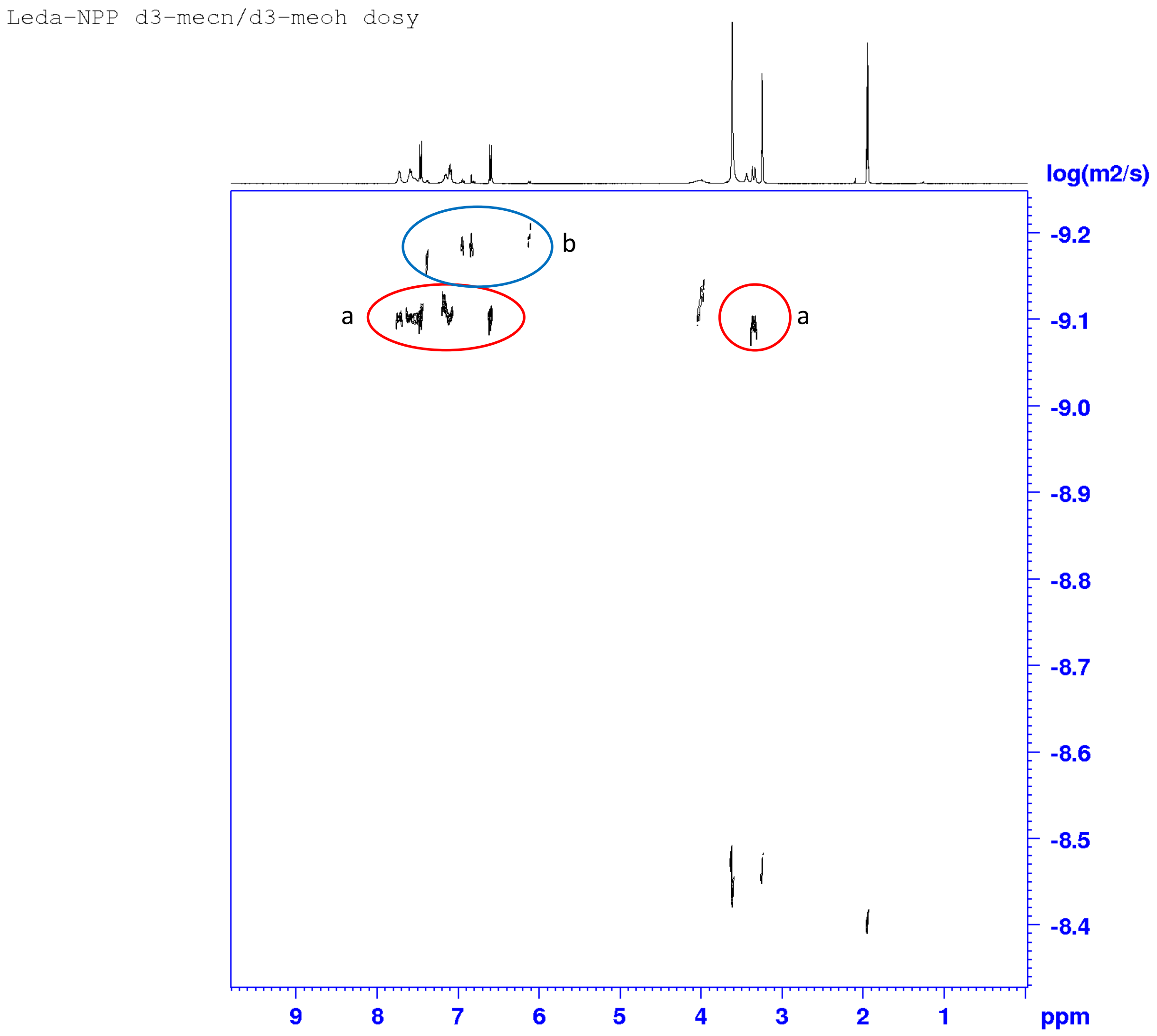

Figure S1.26. DOSY ^{1}H NMR (CD_3OD/CD_3CN 1:2) of $\mathbf{L^2}$ plus $Zn(OTf)_2$ and 1 equivalent of Na_2NPP showing the major (a) and minor (b) set of signals have the same diffusion coefficient.

References

1. R. A. Faulkner, N. J. Patmore, C. R. Rice and C. Slater, Chem. Commun., **2018**, *54*, 9159—9162.
2. SHELXTL Program System, Vers. 5.1, Bruker Analytical X-ray Instruments Inc., Madison, WI, 1998.
3. G. M. Sheldrick, SADABS: A Program for Absorption Correction with the Siemens SMART System, University of Göttingen (Germany), 1996.
4. O. V. Dolomanov, L. J. Bourhis, R. J. Gildea, J. A. K. Howard and H. Puschmann, OLEX2: a complete structure solution, refinement and analysis program. *J. Appl. Cryst.,* **2000**, *42*, 339-341.

B. Biological studies

Methods

Cell lines and culture conditions: All cell lines were purchased from American Type Culture Collection apart from the $p53^{+/+}$ and $p53^{-/-}$ isogenic clones of HCT116 human colorectal adenocarcinoma cells which were a kind gift from Professor Bert Vogelstein (Bunz et al., 1998). The cell lines were maintained as monolayers, at low passage and in antibiotic free media. HT29, DLD-1, HCT116 $p53^{+/+}$ and HCT116 $p53^{-/-}$ are all human colorectal adenocarcinoma cell lines; PSN-1, BxPC-3 and MiaPaCa2 are human pancreatic carcinoma cell lines and A549 and H460 are human non-small cell lung carcinoma cell lines. HT29, DLD-1, PSN-1, BxPC-3, A549 and H460 cell lines were cultured in RPMI-1640 growth media (Sigma) containing 2 mM L-glutamine, 1 mM sodium pyruvate and 10% foetal bovine serum (FBS). HCT116 ($p53^{+/+}$ and $p53^{-/-}$) and MiaPaCa2 cell lines were cultured in Dulbecco's Modified Eagle's Medium (Sigma), 2 mM L-glutamine and 10% FBS. The ARPE-19 human retinal epithelial non-cancer cell line was cultured in DMEM/F12 media (Gibco), 2 mM L-glutamine, 1 mM sodium pyruvate and 10% FBS.

Chemosensitivity studies: The response of cells following a continuous 96 h exposure to test compounds was determined using the MTT assay. $[(\mathbf{L^1})_2M_2]^{4+}$ and $[(\mathbf{L^2})_2M_2]^{4+}$ complexes (M = metal ion, Zn^{2+} or Cu^{2+}) were freshly formed by adding DMSO to the pre-weighed individual components and then mixing by pipetting. The compounds were then further diluted in cell culture media with the final DMSO concentration that cells were exposed to being 0.1% (vehicle control). All cell lines were seeded into 96 well plates at 2×10^3 cells per well and incubated overnight at 37 °C. Following incubation, media was removed and replaced with fresh media containing the test compounds at a range of concentrations. Cells were incubated with these compounds for 96 h after which the media was removed and replaced with fresh media (200μl/well) containing MTT solution at a final concentration of 0.5 mg/mL and cells were then incubated for a further 4h. Media and MTT were then removed, and formazan crystals that had formed were dissolved in 150 μl of DMSO and well absorbance measured at 540 nm. The concentration of test compound required to reduce cell growth by 50% (IC_{50}) was determined from dose response curves. Potency was recorded as the IC_{50} ± standard deviation for three independent experiments. The selectivity index (SI) was defined as the ratio of the mean IC_{50} values for non-cancer to cancer cell lines with values >1 representing selectivity for cancer cells as opposed to non-cancer cells. As the mean IC_{50} values were used to determine SI, experimental error was not determined.

Cell cycle studies: For cell cycle studies, HCT116 and PSN-1 cancer cells and ARPE-19 non-cancer cells were seeded in 25 cm^2 cell culture flasks and 24h post seeding, media was replaced with fresh complete media containing 0.015% DMSO (solvent control), 15 μM $[(\mathbf{L^1})_2Zn_2]^{4+}$ or 15 μM $[(\mathbf{L^2})_2Zn_2]^{4+}$. At 72 h, media containing any non-adhered cells was collected from treated flasks and pooled with adhered cells collected by standard trypsinization. Following centrifugation, cell pellets were washed

in phosphate buffered saline, gently permeabilized (solution 10, Chemometec), and incubated with 10µg/ml DAPI for 5 min at 37°C before analyzing cell DNA content (emission detection at 450 nm for double stranded DNA) by image cytometry (NC3000 image cytometer, Chemometec).

Time lapse microscopy: Real time monitoring and microscopic imaging of cells was performed using Axion BioSystems Lux FL microscopes placed directly inside a cell culture incubator. This enabled the effects of $[(\mathbf{L^2})_2Zn_2]^{4+}$ on the growth and survival of HCT116 cancer cells and ARPE-19 non-cancer cells to be directly monitored over time within the same magnification field of view. Cells were seeded in 25 cm^2 cell culture flasks and 24h post seeding, fresh complete media containing solvent control or 15µM $[(\mathbf{L^2})_2Zn_2]^{4+}$ was added. 3h following the addition of 15 µM $[(\mathbf{L^2})_2Zn_2]^{4+}$ or DMSO solvent control, cell impermeable DNA binding dye propidium iodide (PI) was added to the media at a final concentration of 4µg/ml for the positive staining of dead or dying cells. Lux FL microscopes were focused on a particular field of view and programmed to acquire images every 30 minutes for 48 h in brightfield (to monitor cell proliferation and changes in cell confluency) and in the red fluorescent channel (to detect PI positive staining and monitor cell death induction).

***In ovo* efficacy studies:** *In ovo* experiments using the chick embryo model were performed in accordance with UK legislation and ethical guidelines with all embryos being humanely terminated on day 14 of chick embryonic development (E14) as stipulated by the UK Animals Scientific Procedures Act 1986 (amended 2012). E14 represents two-thirds of the chick embryo gestation period and up to, and including, day E14 the chick embryo is classified as a non-protected, 3Rs compliant model with no animal license or home office approval being required.

Fertilised Shaver Brown hen eggs were purchased from Medeggs Ltd and stored at 14^0C for up to a week. To commence embryonic development (day E0), eggs were incubated on their side in egg trays at 37.8 °C and 45% humidity in a specialized poultry egg incubator (Brinsea OvaEasy 380) for 3 days (with the upwards side of the egg labelled in pencil to orientate for E3 windowing). Incubator shelves were set to alternately tilt 45º from a horizontal position every 45 minutes. On day 3 (E3), the wide base of each egg was gently pierced and 7 mL of albumen were removed to lower the chorioallantoic membrane (CAM) from the eggshell before windowing. Eggs were windowed by piercing a small hole on the labelled side of the egg, applying a 3 cm piece of invisible tape over the eggshell area to be windowed, and cutting using fine scissors a small three sided window (~2cm by ~1 cm by ~2 cm) in the eggshell (Barnett et al., 2022). Windowed eggs (E3) were further incubated without tilting at 37.8°C and 45% humidity until day E7.

On day E7, eggs containing viable embryos were implanted with tumour cells. PSN-1 pancreatic cancer cells growing as monolayers and in logarithmic phase growth were harvested from cell culture flasks by standard trypsinization, counted, pelleted at 200 g for 5 minutes and resuspended in a small volume of sterile PBS to generate a concentrated cell slurry. For efficient engraftment of tumour cells, a small area of the highly vascularized chorioallantoic membrane (CAM) was dried with sterile gauze onto which a 4 mm silicone ring was carefully placed. 4 $x10^6$ PSN-1 cells were pipetted inside the ring

and onto the CAM and eggs were further incubated at 37.8^{0}C and 45% humidity for 3 days. On day E10, viable eggs were treated with 300 µM $[(\mathbf{L^1})_2Zn_2]^{4+}$ or 0.3% DMSO solvent control by carefully pipetting 25 uL into the silicone ring and eggs were then returned to 37.8^{0}C and 45% humidity until embryo termination on day E14.

On day E14, treated embryos were scored as alive or dead and tumours were excised, with PSN-1 tumours typically developing underneath the CAM. Excised tumours were placed in a petri dish in sterile PBS and imaged using a Zeiss SteREO Discovery.V12 stereomicroscope equipped with an Axiocam 305 camera (Zeiss). Any excess CAM tissue attached to the excised tumour was cut or teased away with dissection scissors and tweezers. Excess PBS was removed and tumours were weighed using a precision balance on small cut pieces of parafilm as described (Burns et al., 2021). Tumour volumes, as an additional endpoint measurement to tumour weight determinations, were calculated from microscopy images of excised tumors. Tumour volumes were estimated assuming an ellipsoidal shape using the formula, volume = 4/3π × length/2 × width/2 × height/2, where height is estimated as 2/3 × length, as previously described (Sápi et al., 2015, Rovithi et al., 2017; Sarogni, et al., 2022), with measurements determined from acquired images using Image J.

Histological analysis: Weighed tumours were fixed in 10% neutral buffered formalin for 48h at room temperature and were then transferred to, and stored in, 70% ethanol at 4°C. Samples were tissue processed using an automated Leica ASP200S tissue processor, embedded in paraffin wax (Leica 11150 wax embedding station) and sectioned at 5µm using a rotary microtome (Leica RM2255). Sections were transferred to a water bath at 50^{0}C and mounted on slides. Microscope slides of mounted sections were then deparaffinized using xylene (2 × 5 min), rehydrated through graded ethanol incubations before a 2-minute wash in water and stained with haematoxylin (Thermo Scientific 6765009) for 3 minutes. After differentiation with 10% glacial acetic acid and blueing with tap water by standard histological procedures, sections were then stained with eosin (0.5% solution in water, Carl Roth). Haematoxylin and eosin-stained sections were then viewed using a Keyence VHX-6000 digital microscope.

Supplementary chemosensitivity results

IC_{50} values of the L^1 and L^2 copper complexes and the L^1 and L^2 zinc complexes are presented in Figure S2.1. The order of cancer cell lines is the same as is presented in Figure 7, i.e. arranged from left to right in order of increasing selectivity index for the copper and zinc L^2 complexes. IC_{50} values of the complexes against the ARPE-19 non-cancer cells are also presented. As shown in Figure S2.1, for most of the cancer cell lines, the L^1 metal complexes either showed similar potency or were more potent than the equivalent L^2 metal complex. However, the L^2 zinc complex was much less active towards the ARPE-19 non-cancer cells than the other complexes (Figures S2.1 and S2.2) resulting in its superior cancer cell selectivity compared to the L^2 copper complex across the cancer cell line panel (Figures 7 and S2.2c). This was also true when comparing cancer selectivity against the L^1

complexes, the sole exception being against the H460 lung cancer cell line for which the L^1 copper complex showed superior cancer selectivity (Figure S2.2).

For the zinc L^1 and L^2 complexes, the effects of their pre-incubation with $H_2PO_4^-$, $PhOPO_3^{2-}$ or $O_2NC_6H_4OPO_3^{2-}$ anions before cell exposure, on their potency and selectivity towards selective cell lines was evaluated (Figure S2.3, Figure 7 inset for $H_2PO_4^-$). Activity of zinc L^2 complex against the ARPE-19 non-cancer cells was modestly increased by all three phosphate anions (decreased IC_{50} relative to $[(L^2)_2Zn_2]^{4+}$ alone, Figure S2.3) whereas activity towards the tested cancer cell lines decreased or did not change. This resulted in a small decrease in cancer cell selectivity of the zinc L^2 complex through pre-incubation with phosphate anions. In contrast, pre-incubation of $H_2PO_4^-$ and $PhOPO_3^{2-}$ with zinc L^1 complex decreased its activity against the ARPE-19 non-cancer cells and resulted in a modest increase in the cancer cell selectivity of $[(L^1)_2Zn_2]^{4+}$ (Figure S2.3d, Figure 7 inset). Whilst the differential effects of the anions on the zinc L^1 and L^2 complexes requires further investigation, this indicates how potency and cancer cell selectivity which varies depending on choice of both ligand and metal can be further differentially modulated by anion pre-incubation.

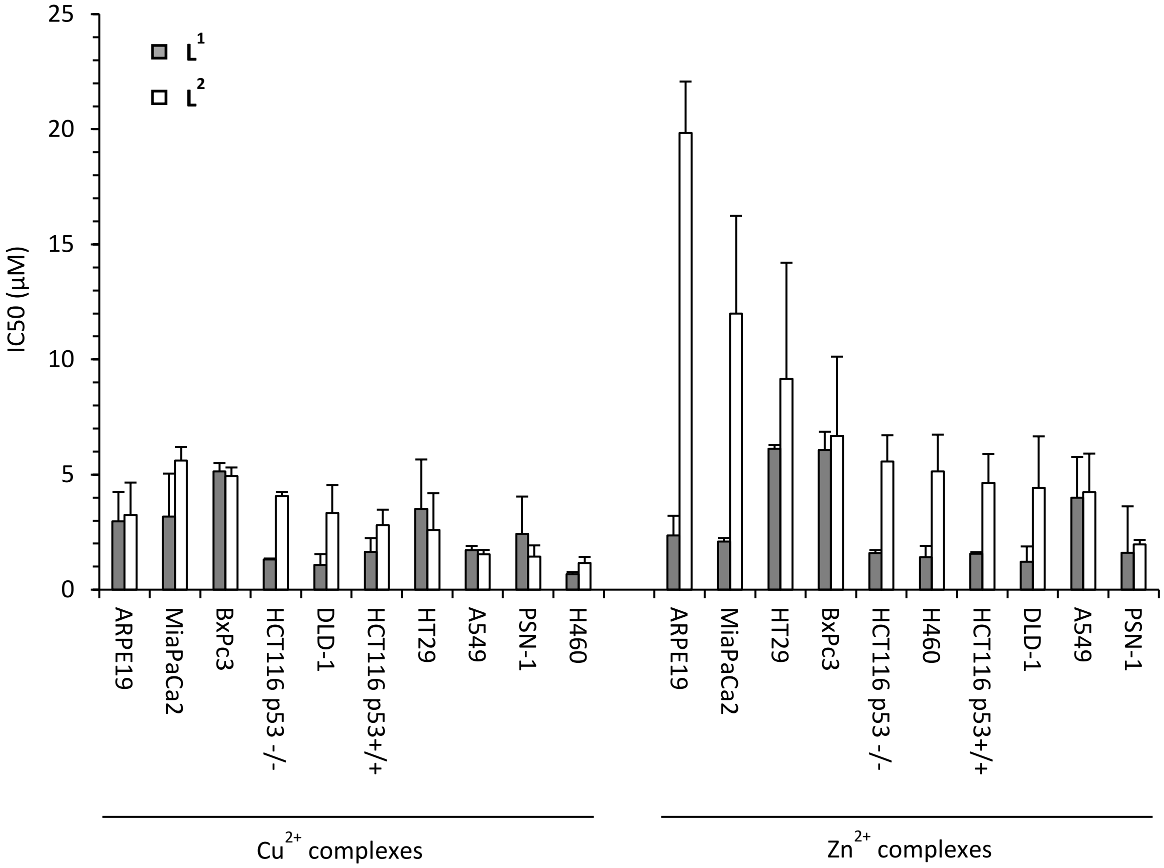

Figure S2.1. Chemosensitivity response of a panel of human cancer cell lines and ARPE-19 non-cancer cells following a 96 hour exposure to $[(L^1)_2Cu_2]^{4+}$, $[(L^2)_2Cu_2]^{4+}$, $[(L^1)_2Zn_2]^{4+}$ and $[(L^2)_2Zn_2]^{4+}$. IC_{50} ± standard deviation from a minimum of three independent experiments. Order of cancer cell lines from left to right is the same as shown in Figure 7.

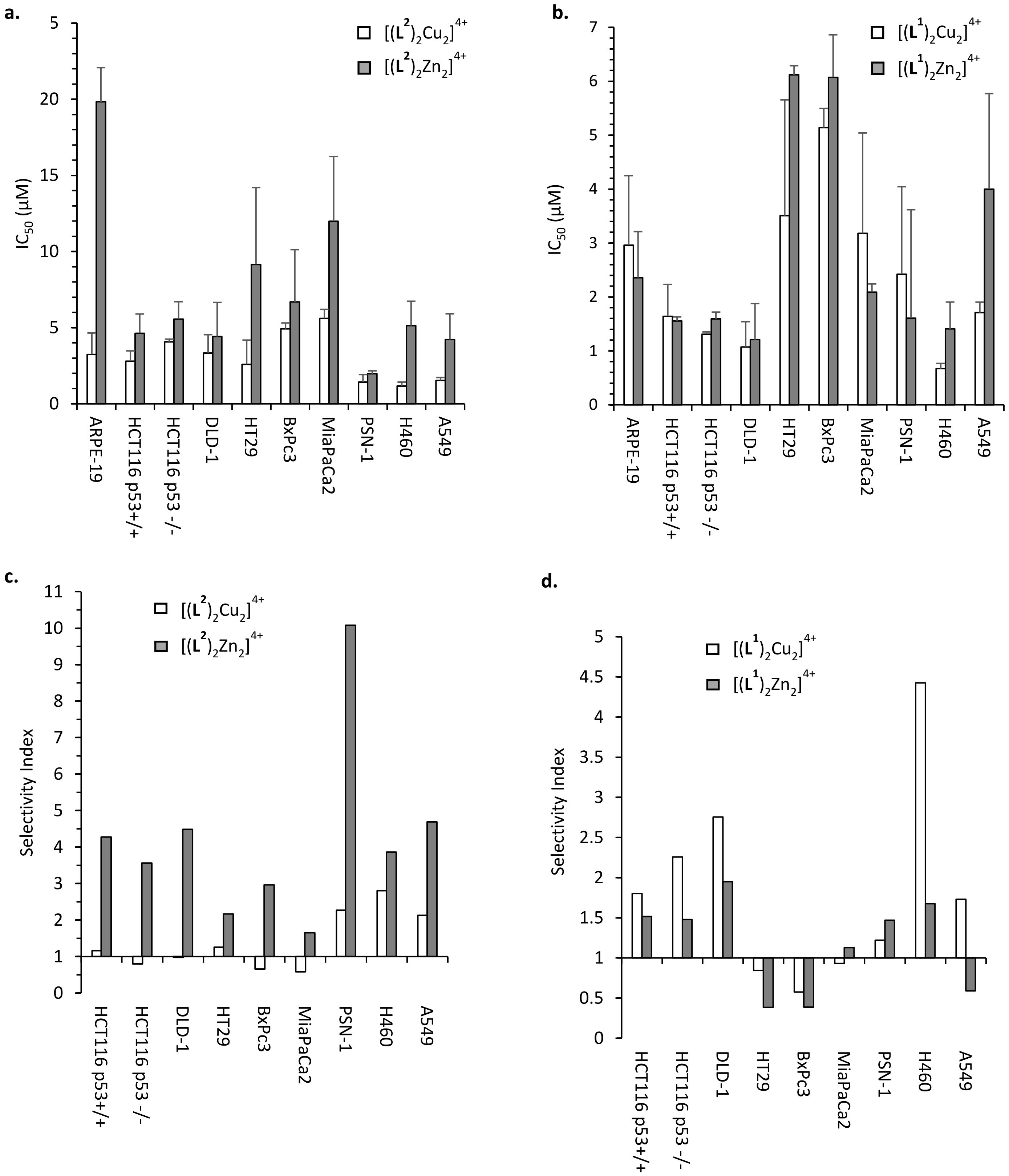

Figure S2.2. Chemosensitivity and cancer cell selectivity of $[(L^2)_2M_2]^{4+}$ and $[(L^1)_2M_2]^{4+}$ complexes comparing the differential influence of Cu^{2+} and Zn^{2+} for each ligand. Panels A and B represent the IC_{50} values ± standard deviation for three independent experiments. Panels C and D represent selectivity indices (defined as the mean IC_{50} value for non-cancer ARPE19 cells divided by IC_{50} values for each cancer cell line) for these compounds.

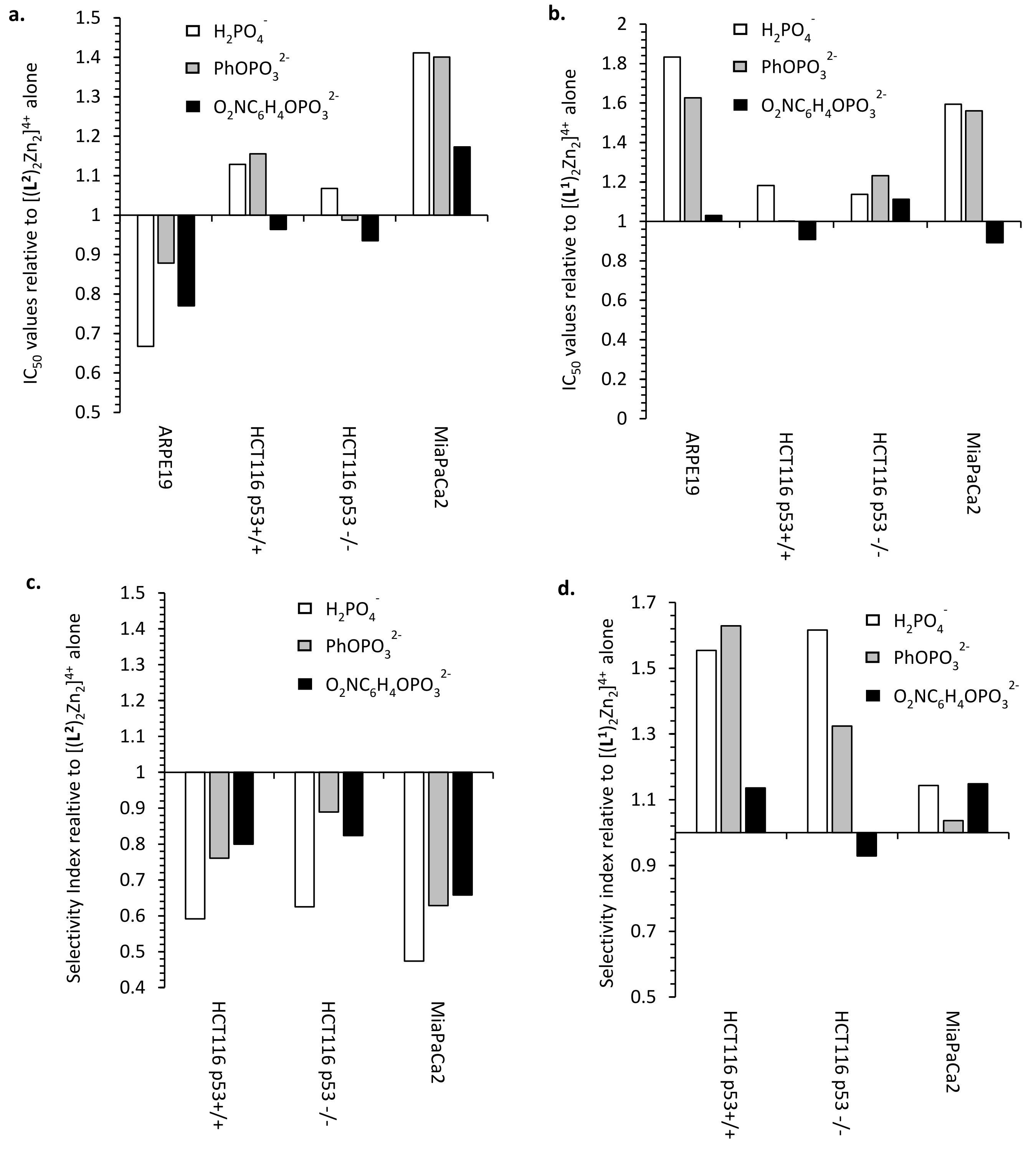

Figure S2.3. Influence of anions on the potency and selectivity *in vitro* of $[(L^1)_2Zn_2]^{4+}$ and $[(L^2)_2Zn_2]^{4+}$. The response of non-cancer (ARPE19) and cancer cells (HCT116 $p53^{+/+}$, HCT116 $p53^{-/-}$ and MiaPaCa2), was determined using the MTT assay following a 96-hour continuous exposure to the naked $[(L^1)_2Zn_2]^{4+}$ and $[(L^2)_2Zn_2]^{4+}$ complexes alone and in the presence of various anions. The influence of anions on both potency (panels A and B) and selectivity (panels C and D) are expressed relative to the naked complex; ratios greater than 1 indicating increased potency or selectivity of the complex plus anion combination relative to the naked complex alone. Each experiment is performed in triplicate but as the results are expressed as a ratio of mean values, error bars are not included on these figures.

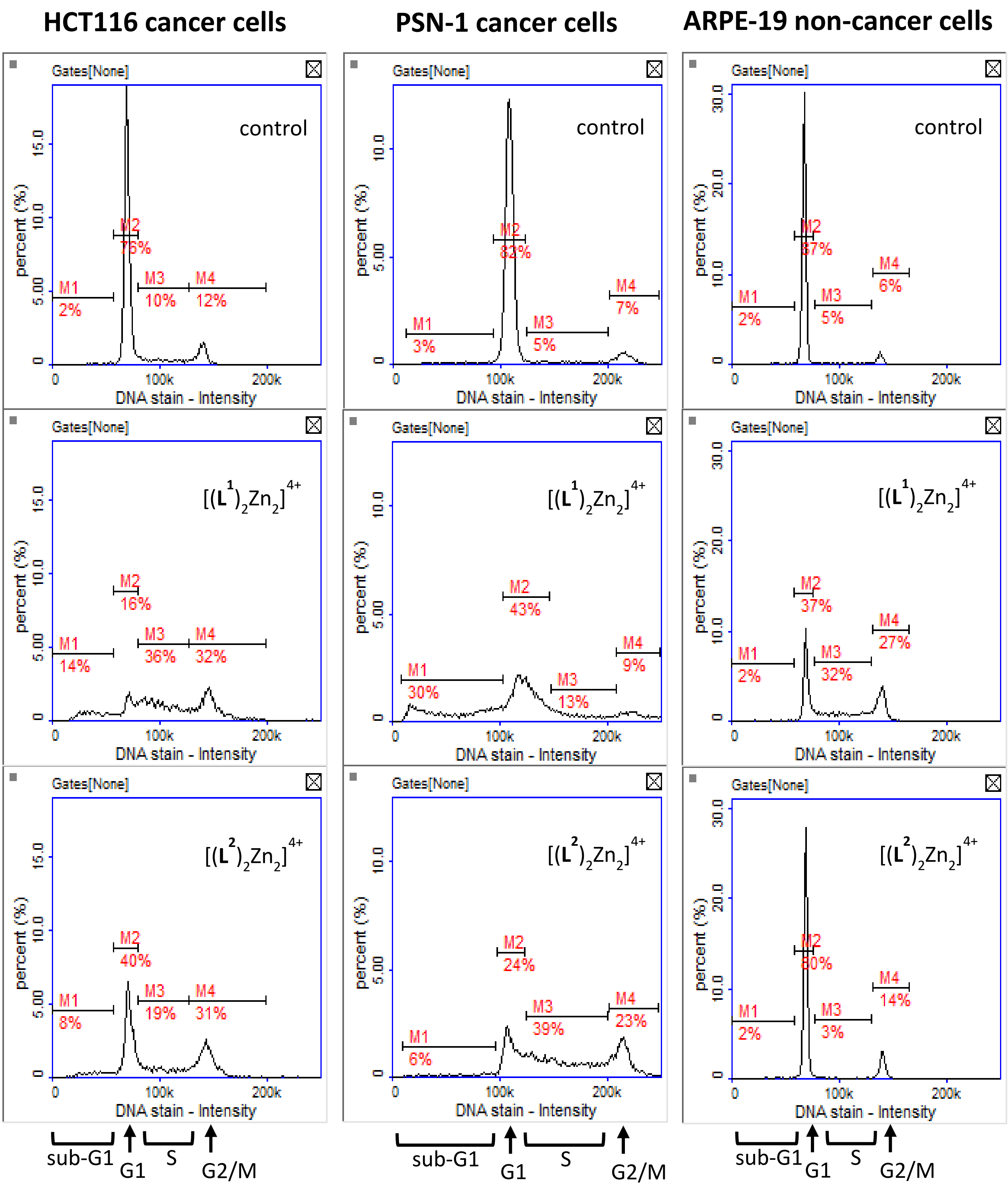

Figure S2.4. Representative cell cycle histograms of HCT116 and PSN-1 cancer cells and ARPE-19 non-cancer cells after 72h treatment with 15µM $[(\mathbf{L^1})_2Zn_2]^{4+}$ or $[(\mathbf{L^2})_2Zn_2]^{4+}$. Percentage of the total cell population in the different phases of the cell cycle are indicated by markers, where M1 represents cells with a sub-G1 phase DNA content (dying cells), M2 represents G1 phase cells, M3 represents S phase cells and M4 represents G2/M phase cells.

HCT116

% cells	control	$[(\mathbf{L^1})_2Zn_2]^{4+}$	$[(\mathbf{L^2})_2Zn_2]^{4+}$
subG1	1.7% ± 0.6	11.7% ± 2.1	8.3% ± 3.5
G1	74.0% ± 1.7	24.0% ± 7.0	37.3% ± 8.3
S	10.7% ± 0.6	24.7% ± 11	16.3% ± 2.5
G2/M	12.7% ± 2.1	35.7% ± 3.2	33.0% ± 5.3

PSN-1

% cells	control	$[(\mathbf{L^1})_2Zn_2]^{4+}$	$[(\mathbf{L^2})_2Zn_2]^{4+}$
subG1	3.6% ± 0.5	17.6% ± 9.7	6.0% ± 0.6
G1	75.4% ± 5.8	54.2% ± 6.1	32.3% ± 6.8
S	9.4% ± 3.3	16.2% ± 4.7	37.0% ± 2.9
G2/M	9.2% ± 2.0	7.8% ± 1.9	18.8% ± 3.8

ARPE-19

% cells	control	$[(\mathbf{L^1})_2Zn_2]^{4+}$	$[(\mathbf{L^2})_2Zn_2]^{4+}$
subG1	2.0% ± 0.0	1.7% ± 0.6	1.7% ± 0.6
G1	88.0% ± 0.6	42.7% ± 12.5	82.7% ± 3.1
S	5.0% ± 0.6	26.0% ± 7.9	3.7% ± 1.2
G2/M	7.0% ± 1.2	28.0% ± 5.6	11.3% ± 3.8

Table S2.1. Quantification of the proportion of HCT116 and PSN-1 cancer cells and ARPE-19 non-cancer cells in different phases of the cell cycle following 72h treatment with 15µM $[(\mathbf{L^1})_2Zn_2]^{4+}$ or $[(\mathbf{L^2})_2Zn_2]^{4+}$. Mean percentage ± standard deviation from a minimum of three independent biological replicates.

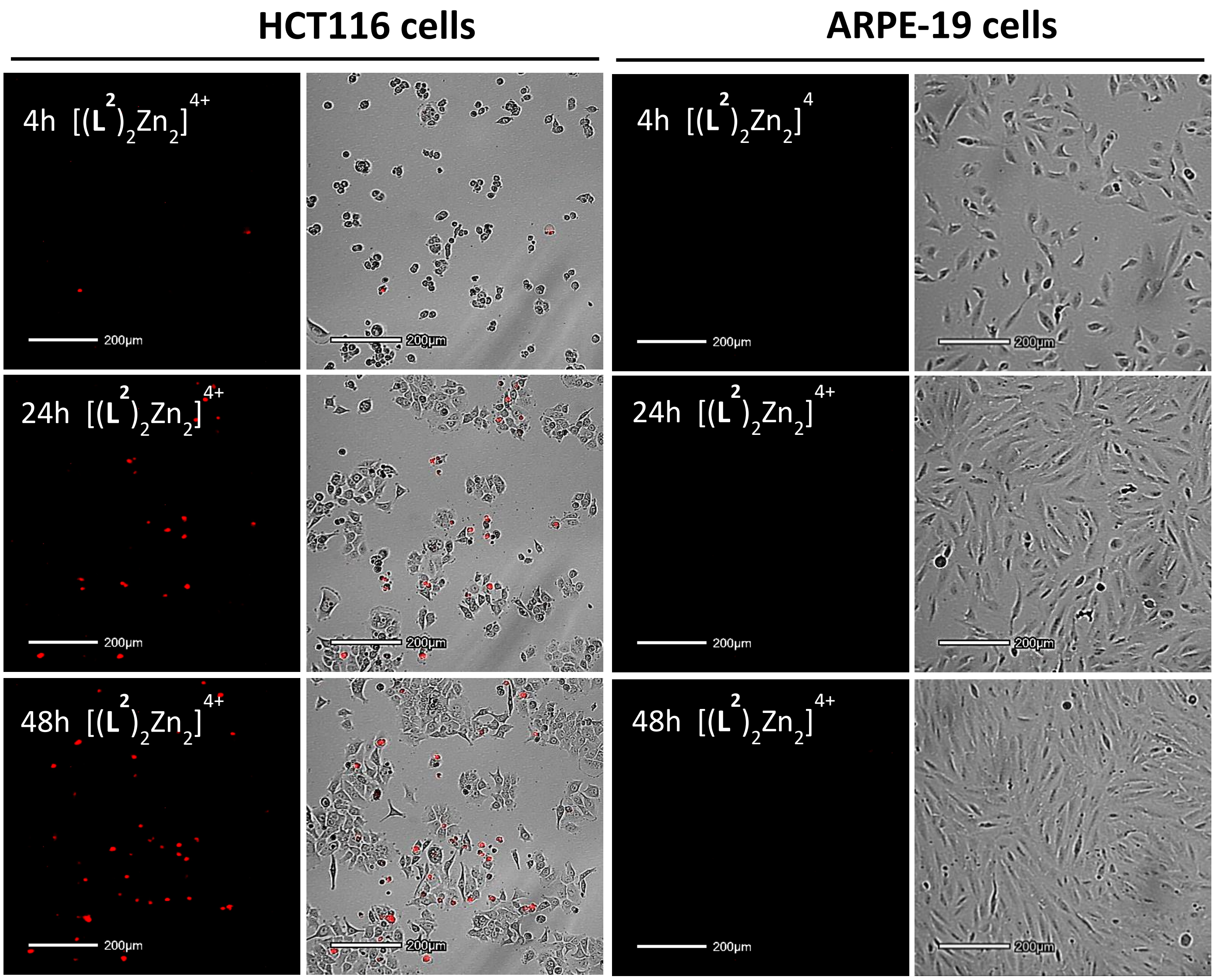

Figure S2.5. Time lapse microscopy of the phenotypic response to $[(\mathbf{L}^2)_2Zn_2]^{4+}$ treatment in HCT116 cancer cells and ARPE-19 non-cancer cells. 3h following the addition of 15µM $[(\mathbf{L}^2)_2Zn_2]^{4+}$, propidium iodide was added to culture media to enable detection of any dead cells, as indicated by red fluorescence. Fluorescent images and fluorescence overlay with brightfield images are shown (Axion BioSystems Lux FL microscope).

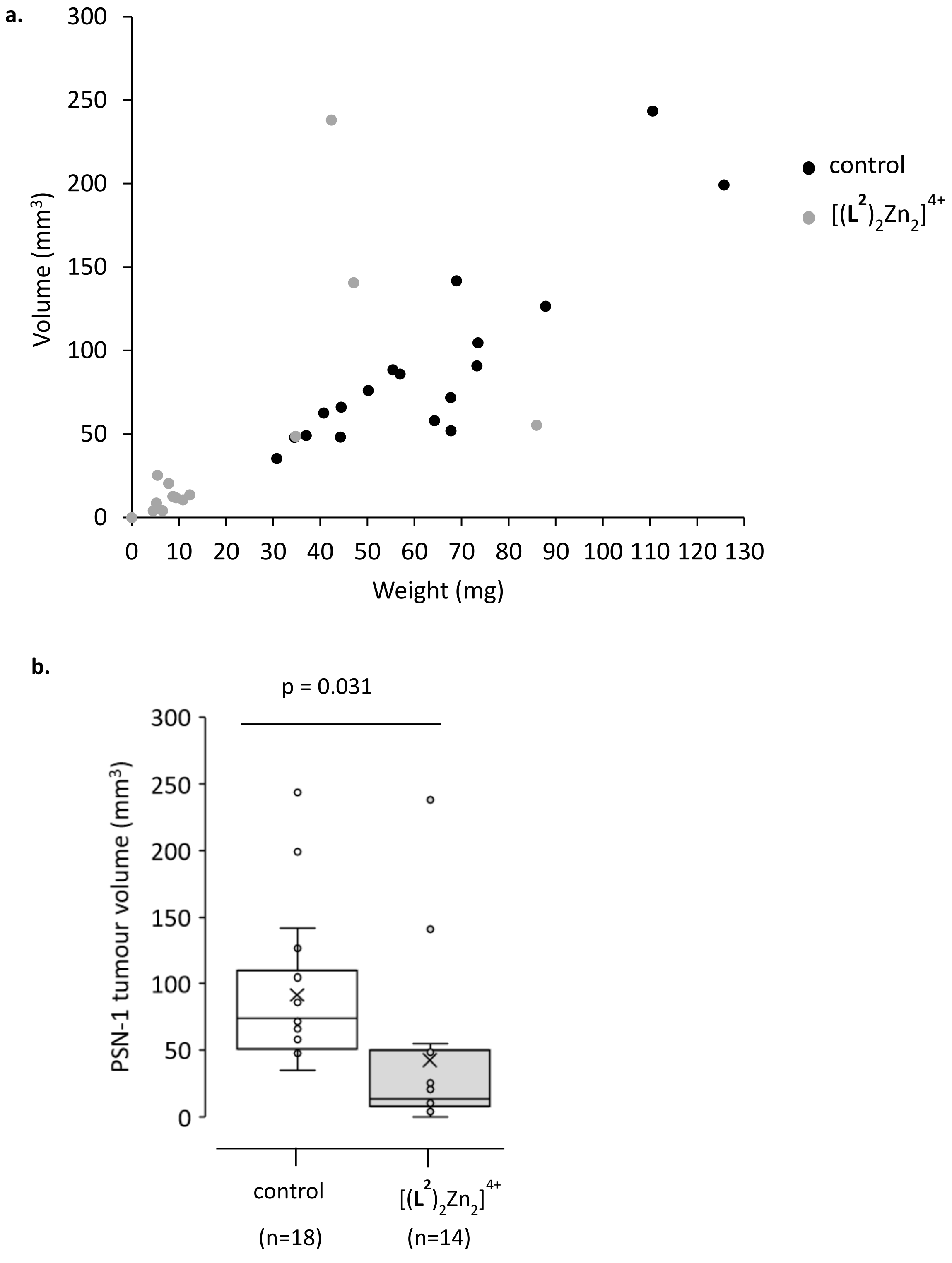

Figure S2.6. Effect of $[(\mathbf{L}^2)_2Zn_2]^{4+}$ treatment on tumour volumes of PSN-1 pancreatic tumours that form *in ovo*. Tumours were topically treated with 300µM $[(\mathbf{L}^2)_2Zn_2]^{4+}$ on embryonic day E10 with tumour weight and volume measurements taken on day E14. **a)** The correlation between tumour weight and tumour volume. **b)** Box plot of the volume of PSN-1 tumours formed *in ovo* and following 4 days treatment with $[(\mathbf{L}^2)_2Zn_2]^{4+}$ or control. Mean (x); number of tumours per treatment group (n). Statistical significance of p=0.031, student's t-test.

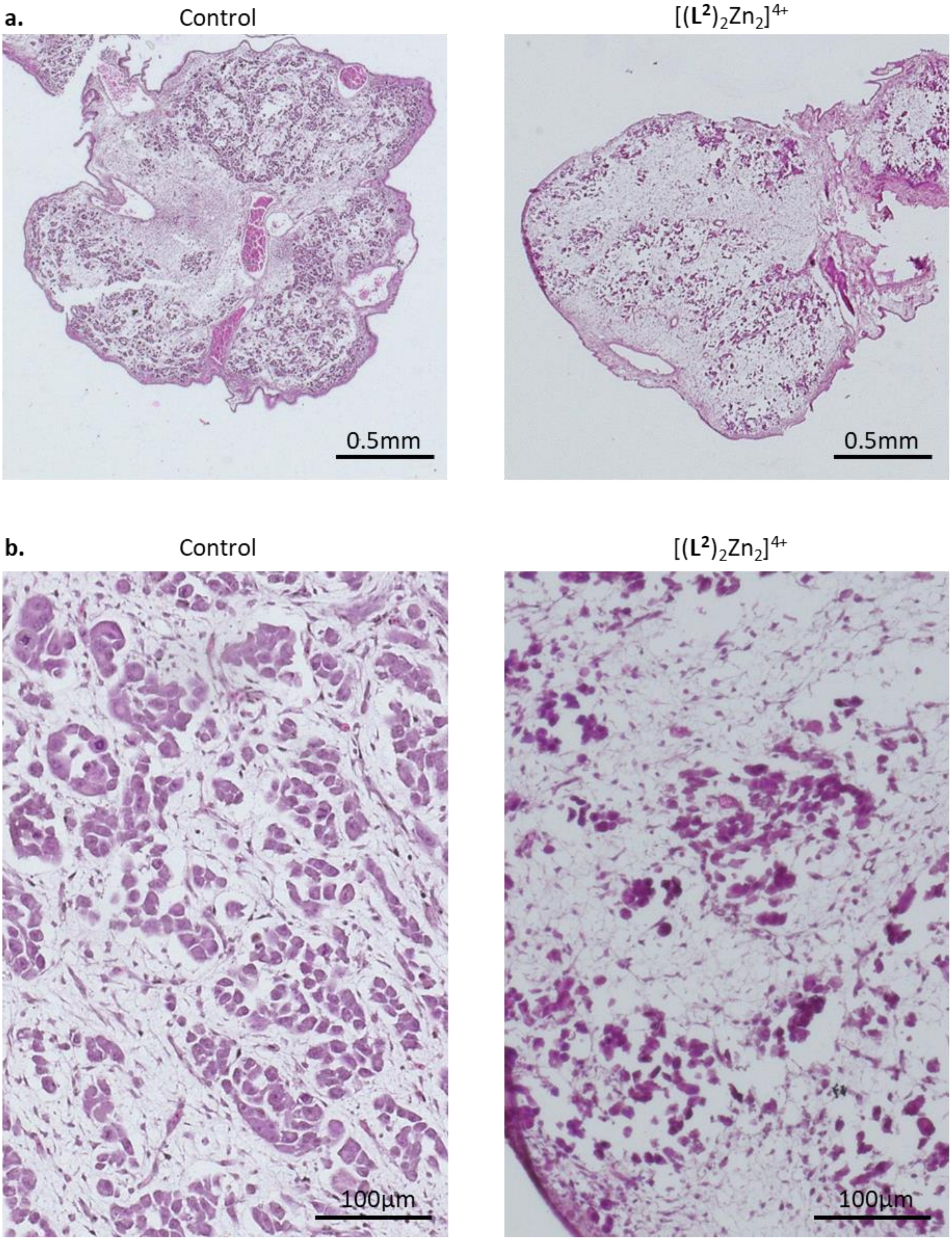

Figure S2.7. Histology of control and $[(\mathbf{L^2})_2Zn_2]^{4+}$ treated PSN-1 pancreatic tumours formed *in ovo* following xenografting of PSN-1 tumour cells on the chorioallantoic membrane (CAM) of the developing chick embryo. Tumours were topically treated with 300µM $[(\mathbf{L^2})_2Zn_2]^{4+}$ or 0.3% DMSO control on embryonic day E10 and on day E14 tumours were excised and fixed. Representative images of control and $[(\mathbf{L^2})_2Zn_2]^{4+}$ treated PSN-1 tumours following H & E staining at x50 magnification (**a**) or x300 magnification (**b**).

Supplementary references

Barnett SE, Herrmann A, Shaw L, Gash EN, Poptani H, Sacco JJ, Coulson JM. The Chick Embryo Xenograft Model for Malignant Pleural Mesothelioma: A Cost and Time Efficient 3Rs Model for Drug Target Evaluation. *Cancers*. 2022; 14(23):5836. doi.org/10.3390/cancers14235836

Bunz F, Dutriaux A, Lengauer C, Waldman T, Zhou S, Brown JP, Sedivy JM, Kinzler KW, Vogelstein B. Requirement for p53 and p21 to sustain G2 arrest after DNA damage. *Science*. 1998; 282(5393):1497-501. doi.org/10.1126/science.282.5393.1497.

Burns JE, Hurst CD, Knowles MA, Phillips RM, Allison SJ. The Warburg effect as a therapeutic target for bladder cancers and intratumoral heterogeneity in associated molecular targets. *Cancer Sci*. 2021; 112(9):3822-3834. doi.org/10.1111/cas.15047

Rovithi M, Avan A, Funel N, Leon LG, Gomez VE, Wurdinger T, Griffioen AW, Verheul HM, Giovannetti E. Development of bioluminescent chick chorioallantoic membrane (CAM) models for primary pancreatic cancer cells: a platform for drug testing. *Sci Rep.* 2017; 7:44686. doi.org/10.1038/srep44686.

Sápi J, Kovács L, Drexler DA, Kocsis P, Gajári D, Sápi Z. Tumor Volume Estimation and Quasi-Continuous Administration for Most Effective Bevacizumab Therapy. *PLoS One*. 2015; 10(11):e0142190. doi.org/10.1371/journal.pone.0142190.

Sarogni P, Mapanao AK, Gonnelli A, Ermini ML, Marchetti S, Kusmic C, Paiar F, Voliani V. Chorioallantoic membrane tumor models highlight the effects of cisplatin compounds in oral carcinoma treatment. *iScience*. 2022; 25(3):103980. do.org/10.1016/j.isci.2022.103980.
